# Abyssomicins from the South China Sea Deep-Sea Sediment *Verrucosispora* sp.: Natural Thioether Michael Addition Adducts as Antitubercular Prodrugs**

**DOI:** 10.1002/anie.201208801

**Published:** 2012-12-06

**Authors:** Qian Wang, Fuhang Song, Xue Xiao, Pei Huang, Li Li, Aaron Monte, Wael M Abdel-Mageed, Jian Wang, Hui Guo, Wenni He, Feng Xie, Huanqin Dai, Miaomiao Liu, Caixia Chen, Hao Xu, Mei Liu, Andrew M Piggott, Xueting Liu, Robert J Capon, Lixin Zhang

**Affiliations:** a Key Laboratory of Pathogenic Microbiology and Immunology, Institute of Microbiology, Chinese Academy of SciencesBeijing, 100190 (China); b Institute for Molecular Bioscience, The University of QueenslandSt. Lucia, QLD 4072 (Australia); c Graduate University of Chinese Academy of SciencesBeijing, 100049 (China); d Department of Medicinal Chemistry, Institute of Materia Medica, Chinese Academy of Medical Sciences & Peking Union Medical CollegeBeijing, 100050 (China); e Department of Chemistry and Biochemistry, University of Wisconsin-La CrosseLa Crosse, WI 54601 (USA); f Department of Pharmacognosy, Faculty of Pharmacy, Assiut UniversityAssiut 71526 (Egypt); g Department of Chemistry and Center for Diagnostics and Therapeutics, Georgia State UniversityAtlanta, Georgia 30303 (USA)

**Keywords:** natural products, prodrugs, polyketides, structure elucidation, tuberculosis

Tuberculosis (TB) is a leading cause of death in the world today, and is exacerbated by the prevalence of multi- (MDR-TB), extensively (XDR-TB), and totally (TDR-TB) drug resistant strains. Despite the threat to human health, existing frontline TB therapeutics remain constrained to a handful of vintage antibiotics prescribed in a combinatorial format to achieve efficacy. The current shortfall in antitubercular drugs demands urgent attention, to develop new antibiotics effective against all strains of tuberculosis.

In responding to this challenge, we screened a library of marine-derived bacteria (4024) and fungi (533) for growth inhibitory activity against Bacille Calmette Guerin (BCG), an attenuated strain of the bovine tuberculosis bacillus *Mycobacterium bovis*.^1^ BCG serves as a nonpathogenic but nevertheless valuable screening surrogate for the far more hazardous and pathogenic *M. tuberculosis*. Our screening detected 27 (0.6 %) extracts with anti-BCG activity, including a South China Sea deep-sea (−2733 m), sediment-derived actinomycete, *Verrucosispora* sp. (MS100128). Bioassay-directed fractionation of a large scale (21 L) culture of MS100128 yielded three new members of the rare class of abyssomicin polyketides, abyssomicins J (**1**), K (**2**), and L (**3**), and the four known^2^ abyssomicins B (**4**), C (**5**), D (**6**), and H (**10**) (Figure 1). All structures were assigned by detailed spectroscopic analysis, with the known abyssomicins **4**–**6** and **10** documented in the Supporting Information, and the new abyssomicins **1**–**3** discussed below.

**Figure 1:**
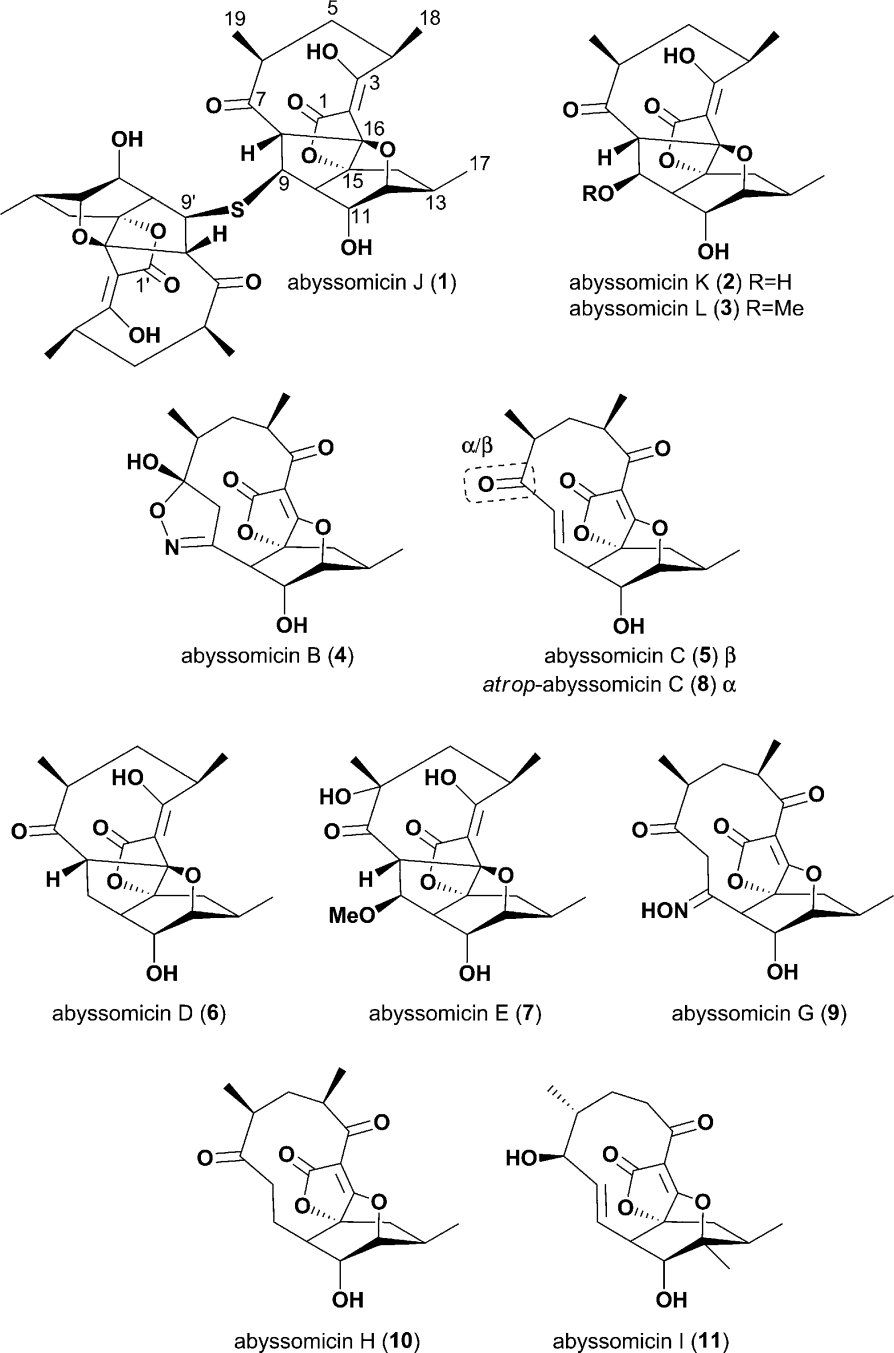
Structure of the abyssomicins 1–11.

The abyssomicins B–D (**4**–**6**) were first reported in 20042a,b from the deep-sea (abyssal) *Verrucosispora* sp. (AB-18-032), since proposed to be the new taxon *Verrucosispora maris* sp. nov.^3^ A subsequent 2007 reinvestigation2c of AB-18-032 led to three additional co-metabolites in the form of abyssomicins G (**9**) and H (**10**), and *atrop*-abyssomicin C (**8**). The deep-sea status of the abyssomicin chemotype was challenged by a 2007 report^4^ of abyssomicin E (**7**) from a Senegalese soil *Streptomyces* sp. (HK10381), a 2010 report^5^ of abyssomicin I (**11**) from a Mexican soil *Streptomyces* sp. (CHI39), and a 2011 report^6^ of *ent*-homoabyssomicins A and B from a German soil *Streptomyces* sp. (Ank 210). Ongoing interest in the synthesis, biosynthesis, and pharmacology of the abyssomicins has been fuelled by the observation that abyssomicin C (**5**), an inhibitor of *p*-aminobenzoic acid (*p*-ABA) biosynthesis (a putative molecular target for next-generation antibiotics),^6^ exhibits promising anti-methicillin-resistant *Staphylococcus aureus* (MRSA)2b and antitubercular^7^ activities.

HRESI-MS measurements on abyssomicin J (**1**) revealed an adduct ion ([*M*+Na]^+^) consistent with a molecular formula of C_38_H_46_O_12_S (Δ*mmu*+0.4). Examination of the ^13^C NMR (CDCl_3_) data (see Table S1 in the Supporting Information) revealed only 19 carbon resonances, thus indicating symmetry. Further analysis of the NMR data revealed a high degree of similarity with those previously reported for abyssomicin D (**6**),^8^ with the most significant difference being the replacement of methylene C9 (*δ*_H_=2.00/1.54 ppm, *δ*_C_=26.1 ppm) in **6** with a thiomethine (*δ*_H_=3.83 ppm, *δ*_C_=41.1 ppm) in **1**. Detailed analysis of 2D COSY, HMBC, and ROESY NMR correlations (see Figure S1 e in the Supporting Information) confirmed a common pentacyclic core between **1** and **6**, with an HMBC correlation from H9 (*δ*_H_=3.83 ppm) to C9 (*δ*_C_=41.1 ppm), thus suggesting dimerization through a C9 to C9′ thioether bridge. A C9 β thioether configuration was assigned by comparing experimental data for H9 (*J*_8,9_=10.8 Hz; *J*_9,10_=3.6 Hz) with calculated values for energy minimized (MM2) *in silico* models of α (*J*_8,9_=6–7 Hz; *J*_9,10_<1 Hz) and β (*J*_8,9_=7–8 Hz; *J*_9,10_=3–4 Hz) thioethers,^4^ and with the literature data for abyssomicin E (**7**; *J*_8,9_=8 Hz; *J*_9,10_=4 Hz). Thus the complete relative stereostructure for **1** could be assigned as shown in Figure 1.

HRESI-MS measurements on abyssomicin s K (**2**) and L (**3**) revealed adduct ions consistent with molecular formulae (**2**: C_19_H_24_O_7_, Δ*mmu*+0.8; **3**: C_20_H_26_O_7_, Δ*mmu*+0.5) attributed to the corresponding H_2_O and MeOH Michael addition adducts of **5**. In support of this hypothesis, the NMR (CDCl_3_) data for **2** and **3** (see Tables S2 and S3 in the Supporting Information) proved to be very similar to those of **1**, with significant differences being limited to replacement of the thiomethine in **1** (*δ*_H_=3.83 ppm; *δ*_C_=41.1 ppm) with a hydroxymethine in **2** (*δ*_H_=4.81 ppm and *δ*_C_ 67.7 ppm), and a methoxymethine in **3** (*δ*_H_=4.43 ppm and *δ*_C_=76.7 ppm; OMe *δ*_H_=3.30 ppm and *δ*_C_=58.2 ppm). The 2D NMR data for **2** and **3** (see Figures S2 e and S3 e in the Supporting Information) also revealed diagnostic correlations supportive of the proposed structures.

Absolute configurations were assigned to **1**–**3** on biogenetic grounds, given that they are co-metabolites of **4**–**6** and **8**, all of which have been assigned to a common antipodal series.^2^ Also supportive of this biosynthetic relationship, we demonstrated that **1**–**3** could be formed as Michael addition adducts of **5**. For example, a sample of **5** exposed to 0.1 m Na_2_S resulted in near quantitative conversion into three products. The major product was identified as **1**, while the minor products were identified as the intermediate thiol **12** and its oxidation product, the sulfonic acid **14** (Figure 2). By contrast, exposure of **5** to 0.05 m NaOH returned only a single product identified as **2**, while exposure to 0.5 m TFA led to a mixture of **2** and the new isomer **15** (Figure 2 and Scheme 1). The structure for **15** was assigned by detailed spectroscopic analysis (see Figure S10 c in the Supporting Information), and its formation rationalized as an acid-mediated H_2_O Michael addition adduct of **5**, but lacking the cascading second intramolecular Michael addition needed to form the caged-carbon skeleton of **2**. Exposure of **15** to 0.05 m NaOH resulted in quantitative conversion into **2**, while exposure of **5** to 0.5 m TFA in MeOH resulted in facile conversion into a single product, which was identified as **3** (see Figures S17 and S18 in the Supporting Information). Significantly, this latter transformation proceeded (albeit at a far slower rate) without exposure to acid, during handling/storage of **5** in MeOH. The observations listed above confirm that **1**–**3** are biosynthetically related to and are likely derived from **5**, and reveal for the first time an acid-mediated strategy capable of accessing a new abyssomicin scaffold (i.e. **15**).

**Figure 2:**
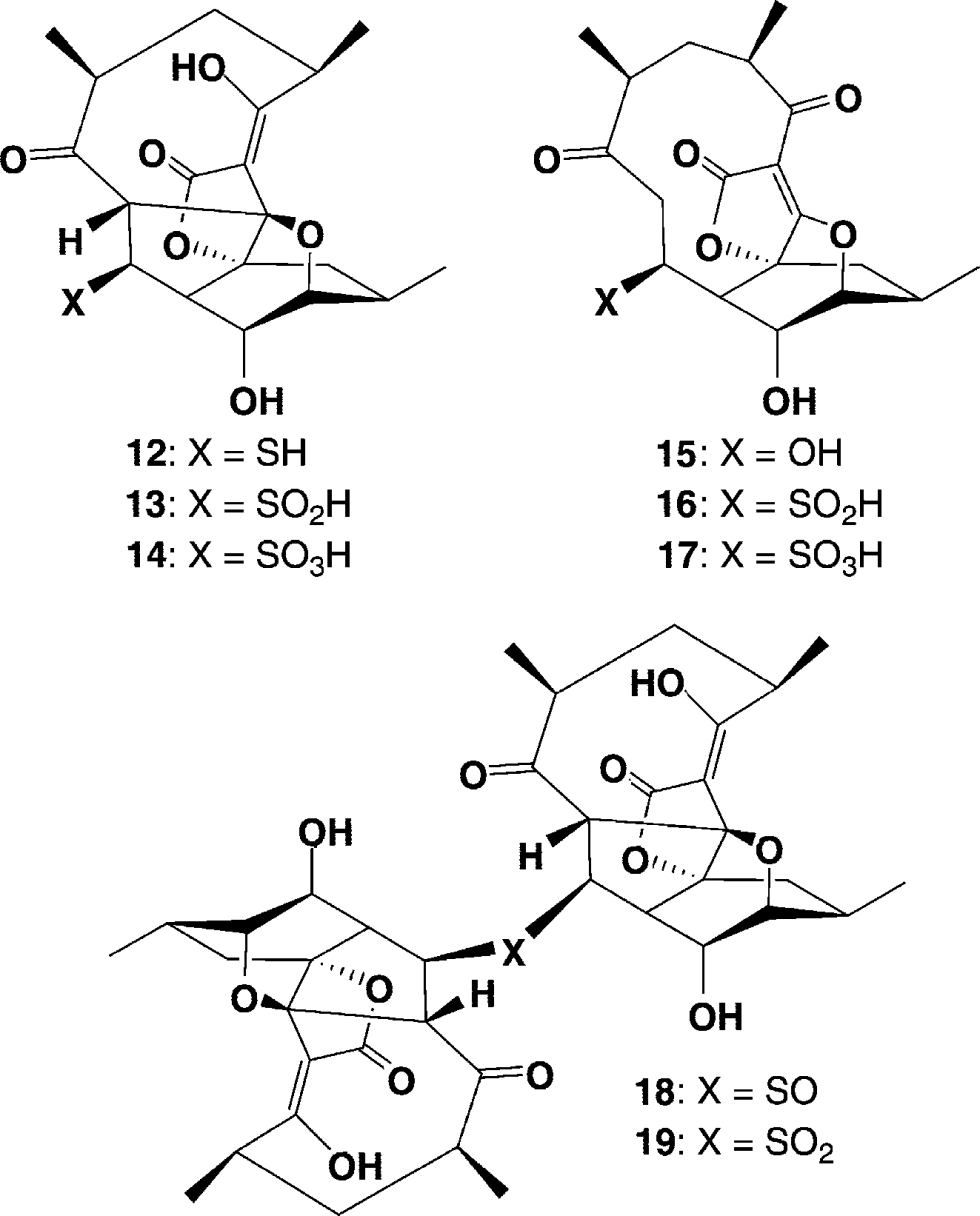
The abyssomicin semisynthetic analogues 12–19.

**scheme 1:**
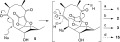
Michael addition on 5 to yield the adducts 1–3 and 15. a) Nu=Na_2_S. b) Nu=NaOH. c) Nu=MeOH. d) Nu=H_2_O/H^+^. See the Supporting Information for details.

To address the possibility that one or more of the compounds **1**–**3** were handling artifacts, a fresh EtOAc extract of a small-scale culture of *Verrucosispora* sp. (MS100128) was prepared and subjected to HPLC-DAD-MS analysis using MeCN/H_2_O, thus avoiding exposure to acid, base, and alcoholic solvents. This analysis detected all the compounds **1**–**3**, as well as the metabolites **4**–**6** and **10**, thus confirming their natural product status (see Figure S13 in the Supporting Information).

Among the known abyssomicins, only the atropisomers **5** and **8** have been attributed anti-TB properties—against the fast growing nonpathogenic *M. smegmatis*, the TB surrogate BCG, and *M. tuberculosis* (H37Rv)^7^—and thus emphasized the critical structure–activity importance of the Michael acceptor enone moiety.2a Given this history, we were initially surprised to discover that, along with **5**, the thioether **1** was the principle anti-TB agent in *Verrucosispora* sp. (MS100128). Indeed, the anti-BCG activities for **1** (MIC 3.125 μg mL^−1^) compared favorably with those of **5** (MIC 6.25 μg mL^−1^; see Table S11 in the Supporting Information). To explain this apparent departure from the established Michael acceptor pharmacophore paradigm, we hypothesized that **1** was a natural prodrug undergoing in situ reverse Michael addition to deliver an abyssomicin anti-TB antibiotic (presumably **5** and/or **8**). As **1** was stable during isolation and handling, we speculated that the reverse Michael addition process required activation by in situ enzymatic oxidation (i.e. P450). This view was based in part on a review of the literature, which confirmed that P450 enzymes can transform thioethers by way of sulfoxides into sulfones, and that sulfones can undergo a reverse Michael addition. For example, the synthetic vasodilator thioether flosequinan sulfide is transformed by rat and human liver P450 enzymes into its sulfoxide and sulfone,^9^ while cancer cell enzymatic oxidation of the synthetic thioether prodrugs of brefeldin yield sulfones, which in turn undergo rapid reverse Michael addition to deliver brefeldin.^10^ In yet another example of sulfone-mediated reverse Michael addition, the semisynthetic sulfone antibiotic dalfopristin undergoes metabolism in human plasma to give the natural product Michael acceptor pristinamycin IIA.^11^ These examples notwithstanding, based on our hypothesis, **1** would represent the first example of a natural thioether adduct (dimer or otherwise) which serves as a prodrug for its associated Michael acceptor.

To test this hypothesis in vitro, a MeCN/H_2_O solution of **1** was treated with the oxidizing reagent Oxone (as a chemical P450 surrogate)^12^ to yield four products identified by spectroscopic analysis as the sulfoxide **18**, sulfone **19**, sulfonic acid **14**, and *atrop*-abyssomicin C (**8**). The sulfoxide **18**, identified by HPLC-DAD-HRESI-MS, proved unstable to handling as it undergoes rapid air oxidation to the sulfone **19**. Likewise, although the sulfone **19** was sufficiently stable for ^1^H NMR analysis, when it was handled in MeCN at 40 °C (1 h) it underwent a reverse Michael addition to yield **8**, together with four minor intermediates. The latter products were identified by HPLC-DAD-ESI-MS as the sulfinic acids **13** and **16**, and the sulfonic acids **14** and **17**. Although a sample of **14** could be purified and characterized by ^1^H NMR spectroscopy, even short (10 min) exposure to MeCN at room temperature led to equilibration of a **14**/**17** mixture, and heating to 70 °C (12 h) transformed this mixture into **8**. Notably, after oxidative activation by Oxone to form the sulfoxide **18**, all subsequent transformations leading to **8** could be accommodated by air oxidation and inherent chemical reactivity. Based on these observations, a plausible mechanism for the transformation of **1** into **8**, inclusive of the intermediates **13** and **14** and **16**–**19**, is illustrated in Scheme 2 (see Figures S19–21 in the Supporting Information). In this mechanism, the formation of the single atropisomer **8** (i.e. no trace of **5**) was particularly interesting and prompted closer examination.

**scheme 2:**
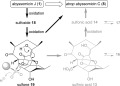
Oxidative activation of the prodrug abyssomicin J (1), thus leading to *atrop*-abyssomicin C (8).

To better understand the chemical and biological significance of atropisomer selectivity in the reverse Michael addition transformation of **1** into **8**, we carried out analytical studies on the Michael acceptors **5** and **8**. Nicolaou and Harrison demonstrated,^13^ and we have independently confirmed (see Figures S12 a and S12 b in the Supporting Information), that **5** and **8** equilibrate under anhydrous acid-mediated conditions (e.g. CDCl_3_). Importantly, as this equilibration was not evident under nonanhydrous in vivo conditions, we reasoned that **5** and **8** acted independently as anti-TB agents, with an antibiotic potency correlated to their respective strengths as Michael acceptors. Building on this hypothesis, and having established **8** as the sole atropisomer arising from a reverse Michael addition, we reasoned that **8** was optimally configured as a superior Michael acceptor (compared to its atropisomer **5**). To test this hypothesis, separate MeCN/H_2_O solutions of **5** and **8** were exposed to 0.1 m TFA to initiate an acid-mediated Michael addition leading to **15**. A time course (18 h) analysis clearly established **8** as a far more potent Michael acceptor (Figure 3), and is consistent with its prior history as a superior antimicrobial agent.2c, 13 The high Michael acceptor potency of **8** also suggested a low in vivo half-life. Consistent with all of the above, we detected low levels of **8** in BCG cells exposed to **1** (see Figure S25 in the Supporting Information).

**Figure 3:**
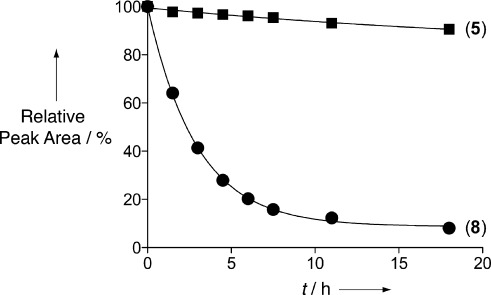
HPLC (*λ*=254 nm) analysis of 0.1 mg mL^−1^ solutions of abyssomicin C (5) and *atrop*-abyssomicin C (8) exposed to 0.1 m TFA in 90 % H_2_O/MeCN at 40 °C.

In summary, our investigations into the anti-TB properties of the South China Sea deep-sea *Verrucosispora* sp. (MS100128) led to the isolation, identification, and anti-TB evaluation of new (**1**–**3**) and known (**4**–**6, 10**) abyssomicins. Structures were assigned to **1**–**3** on the basis of detailed spectroscopic analysis, biosynthetic considerations, mechanistic studies, and semisynthesis from the co-metabolite **5**. Detailed analytical studies into abyssomicin Michael addition chemistry informed our understanding of the chemical reactivity, stability, and anti-TB properties of this rare structure class. We established **8** as a far more potent Michael acceptor than **5**, and used this to rationalize its superior antibacterial properties. We transformed **5** into the Michael adduct **1** and used both in vitro and cell-based analytical studies to demonstrate that **1** can act as a prodrug, thus responding to oxidative activation to selectively deliver the anti-TB antibiotic **8**.

Our studies make a contribution beyond the specifics of the abyssomicin pharmacophore by drawing attention to the possible utility of thioether Michael addition adducts as a means to stabilize highly reactive Michael acceptors, thereby enhancing bioavailability and improving therapeutic potential. The thioether Michael adduct prodrug concept, inspired by abyssomicins from the South China Sea, offers a promising new approach to “chemically package” bioactive Michael acceptors, thus improving their chances of being developed into clinically useful drugs.

## Notes

Detailed facts of importance to specialist readers are published as ”Supporting Information”. Such documents are peer-reviewed, but not copy-edited or typeset. They are made available as submitted by the authors.

## References

[1a] Zhang L, Yan K, Zhang Y, Huang R, Bian J, Zheng C, Sun H, Chen Z, Sun N, An R, Min F, Zhao W, Zhuo Y, You J, Song Y, Yu Z, Liu Z, Yang K, Gao H, Dai H, Zhang X, Wang J, Fu C, Pei G, Liu J, Si Z, Goodfellow M, Jiang Y, Kuai J, Zhou G, Chen X (2007). Proc. Natl. Acad. Sci. USA.

[1b] Ashforth EJ, Fu C, Liu X, Dai H, Song F, Guo H, Zhang L (2010). Nat. Prod. Rep.

[2a] Bister B, Bischoff D, Ströbele M, Riedlinger J, Reicke A, Wolter F, Bull AT, Zähner H, Fiedler H-P, Süssmuth RD Angew. Chem.

[2b] Riedlinger J, Reicke A, Zähner H, Krismer B, Bull AT, Maldonado LA, Ward AC, Goodfellow M, Bister B, Bischoff D, Süssmuth RD, Fiedler H-P (2004). J. Antibiot.

[2c] Keller S, Nicholson G, Drahl C, Sorensen E, Fiedler H-P, Süssmuth RD (2007). J. Antibiot.

[3] Goodfellow M, Stach JEM, Brown R, Bonda ANV, Jones AL, Mexson J, Fiedler H-P, Zucchi TD, Bull AT (2012). Antonie van Leeuwenhoek.

[4] Niu X-M, Li S-H, Görls H, Schollmeyer D, Hilliger M, Grabley S, Sattler I (2007). Org. Lett.

[5] Igarashi Y, Yu L, Miyanaga S, Fukuda T, Saitoh N, Sakurai H, Saiki I, Alonso-Vega P, Trujillo ME (2010). J. Nat. Prod.

[6] Abdalla MA, Yadav PP, Dittrich B, Schüffler A, Laatsch H (2011). Org. Lett.

[7] Freundlich JS, Lalgondar M, Wei J-R, Swanson S, Sorensen EJ, Rubin EJ, Sacchettini JC (2010). Tuberculosis.

[8] Fiedler H-P, Süssmuth R, Zähner H, Bull A, Tübingen Universität

[9] Kashiyama E, Yokoi T, Odomi M, Funae Y, Inoue K, Kamataki T (1997). Drug Metab. Dispos.

[10] Argade AB, Devraj R, Vroman JA, Haugwitz RD, Hollingshead M, Cushman M (1998). J. Med. Chem.

[11] Le LA, Pasquier O, Montay G (1998). J. Chromatogr. B.

[12] Trost BM, Curran DP (1981). Tetrahedron Lett.

[13] Nicolaou KC, Harrison ST Angew. Chem.

