# Supplementary material for: Abyssomicins from the South China Sea Deep-Sea Sediment Verrucosispora sp.: Natural Thioether Michael Addition Adducts as Antitubercular Prodrugs
Source: Angew Chem Int Ed Engl. 2012 Dec 6;52(4):1231–4. doi: 10.1002/anie.201208801 (PMC3563217; doi:10.1002/anie.201208801)
Supplement: Supplementary file 1 [file anie0052-1231-sd1.pdf]

Angewandte Chemie
Eine Zeitschrift der Gesellschaft Deutscher Chemiker

Supporting Information

© Wiley-VCH 2013

69451 Weinheim, Germany

Abyssomicins from the South China Sea Deep-Sea Sediment *Verrucosispora* sp.: Natural Thioether Michael Addition Adducts as Antitubercular Prodrugs**

Qian Wang, Fuhang Song, Xue Xiao, Pei Huang, Li Li, Aaron Monte, Wael M. Abdel-Mageed, Jian Wang, Hui Guo, Wenni He, Feng Xie, Huanqin Dai, Miaomiao Liu, Caixia Chen, Hao Xu, Mei Liu, Andrew M. Piggott, Xueting Liu, Robert J. Capon,* and Lixin Zhang**

anie_201208801_sm_miscellaneous_information.pdf

Table of Contents

General experimental details 3
Collection, cultivation, extraction, isolation, purification 3
Structure elucidation 4
Characterization of compounds 5
Bioassays 6
Biotransformation of abyssomicin J by BCG 7

Figure S1a ^{1}H NMR spectrum (600 MHz, $CDCl_3$) of abyssomicin J (**1**) 8
Figure S1b ^{13}C NMR spectrum (150 MHz, $CDCl_3$) of abyssomicin J (**1**) 9
Figure S1c UV-vis spectrum of abyssomicin J (**1**) 10
Figure S1d CD spectrum of abyssomicin J (**1**) in MeOH 10
Table S1 NMR data (600 MHz, $CDCl_3$) for abyssomicin J (**1**) 11
Figure S1e Selected 2D NMR correlations for abyssomicin J (**1**) 11

Figure S2a ^{1}H NMR spectrum (600 MHz, $CDCl_3$) of abyssomicin K (**2**) 12
Figure S2b ^{13}C NMR spectrum (150 MHz, $CDCl_3$) of abyssomicin K (**2**) 13
Figure S2c UV-vis spectrum of abyssomicin K (**2**) 14
Figure S2d CD spectrum of abyssomicin K (**2**) in MeOH 14
Table S2 NMR data (600 MHz, $CDCl_3$) for abyssomicin K (**2**) 15
Figure S2e Selected 2D NMR correlations for abyssomicin K (**2**) 15

Figure S3a ^{1}H NMR spectrum (600 MHz, $CDCl_3$) of abyssomicin L (**3**) 16
Figure S3b ^{13}C NMR spectrum (150 MHz, $CDCl_3$) of abyssomicin L (**3**) 17
Figure S3c UV-vis spectrum of abyssomicin L (**3**) 18
Figure S3d CD spectrum of abyssomicin L (**3**) in MeOH 18
Table S3 NMR data (600 MHz, $CDCl_3$) for abyssomicin L (**3**) 19
Figure S3e Selected 2D NMR correlations for abyssomicin L (**3**) 19

Figure S4a ^{1}H NMR spectrum (600 MHz, methanol-d_4) of abyssomicin B (**4**) 20
Figure S4b ^{13}C NMR spectrum (150 MHz, methanol-d_4) of abyssomicin B (**4**) 21
Figure S4c UV-vis spectrum of abyssomicin B (**4**) 22
Table S4 NMR data (600 MHz, methanol-d_4) for abyssomicin B (**4**) 23

Figure S5a ^{1}H NMR spectrum (600 MHz, $CDCl_3$) of abyssomicin C (**5**) 24
Figure S5b ^{13}C NMR spectrum (150 MHz, $CDCl_3$) of abyssomicin C (**5**) 25
Figure S5c UV-vis spectrum of abyssomicin C (**5**) 26
Figure S5d CD spectrum of abyssomicin C (**5**) in MeOH 26
Table S5 NMR data (600 MHz, $CDCl_3$) for abyssomicin C (**5**) 27

Figure S6a ^{1}H NMR spectrum (400 MHz, methanol-d_4) of abyssomicin D (**6**) 28
Figure S6b ^{13}C NMR spectrum (100 MHz, methanol-d_4) of abyssomicin D (**6**) 29
Figure S6c UV-vis spectrum of abyssomicin D (**6**) 30
Figure S6d CD spectrum of abyssomicin D (**6**) in MeOH 30
Table S6 NMR data (400 MHz, methanol-d_4) for abyssomicin D (**6**) 31

Figure S7a ^{1}H NMR spectrum (600 MHz, $CDCl_3$) of abyssomicin H (**10**) 32
Figure S7b ^{13}C NMR spectrum (150 MHz, $CDCl_3$) of abyssomicin H (**10**) 33
Figure S7c UV-vis spectrum of abyssomicin H (**10**) 34
Figure S7d CD spectrum of abyssomicin H (**10**) in MeOH 34
Table S7 NMR data (600 MHz, $CDCl_3$) for abyssomicin H (**10**) 35

Figure S8a ^{1}H NMR spectrum (600 MHz, methanol-d_4) of *atrop*-abyssomicin C (**8**) 36
Figure S8b UV-vis spectrum of *atrop*-abyssomicin C (**8**) 37

Figure S9a ^{1}H NMR spectrum (600 MHz, methanol-d_4) of abyssomicin **14** 38
Figure S9b UV-vis spectrum of abyssomicin **14** 39

Figure S10a ^{1}H NMR spectrum (600 MHz, $CDCl_3$) of abyssomicin **15** 40
Figure S10b UV-vis spectrum of abyssomicin **15** 41
Table S10 NMR data (600 MHz, $CDCl_3$) for abyssomicin **15** 42
Figure S10c. Selected 2D NMR correlations for abyssomicin **15** 42

Figure S11a ^{1}H NMR spectrum (600 MHz, methanol-d_4) of sulfone (**19**) 43
Figure S11b UV-vis spectrum of sulfone (**19**) 44

Figure S12a. Proposed mechanism for acid-catalyzed Michael addition equilibration of atropisomers **5** and **8** 45
Figure S12b. HPLC analysis of abyssomicin (**5**) treated with HCl/Et_2O in $CHCl_3$ 46

Figure S13. HPLC analysis of crude extracts and purified abyssomicins **1**–**6**, **10** 47

Figure S14. HPLC analysis of abyssomicin (**5**) treated with 0.1 M Na_2S in 50% H_2O/MeCN 48
Figure S15. HPLC analysis of abyssomicin C (**5**) treated with 0.05 M NaOH in 50% H_2O/MeCN 48
Figure S16. HPLC analysis of abyssomicin C (**5**) treated with 0.5 M TFA in 50% H_2O/MeCN 49
Figure S17. HPLC analysis of abyssomicin C (**5**) treated with 0.5 M TFA in MeCN and then 0.05 M NaOH 49
Figure S18. HPLC analysis of abyssomicin C (**5**) treated with 0.5 M TFA in MeOH 50

Figure S19. HPLC analysis of abyssomicin J (**1**) treated with 2 eq. Oxone in 50% H_2O/MeCN 51
Figure S20. HPLC analysis of sulfone (**19**) after heating for 1 h at 40 °C in MeCN 51
Figure S21. HPLC analysis of **14** and **17** after heating in MeCN 52

Figure S22. HPLC analysis of **8** treated with 0.1 M TFA in 90% H_2O/MeCN – time-course over 18 h 53
Figure S23. HPLC analysis of **5** treated with 0.1 M TFA in 90% H_2O/MeCN – time-course over 18 h 54

Table S11 Antimicrobial activities of abyssomicins **1**–**6** and **10** 55
Figure S24. Dose-dependent inhibition of BCG by abyssomicin J (**1**) 55

Figure S25. HPLC-DAD-HRESI(+)MS analysis of BCG cells exposed to abyssomicin J (**1**) 56

General experimental details

Optical rotations were obtained on a Perkin-Elmer Model 343 polarimeter. UV-vis spectra were obtained on a Cary 50 spectrophotometer. CD spectra were recorded on a JASCO J-815 Spectropolarimeter. NMR spectra were obtained on a Bruker Avance DRX600 or DRX400 spectrometer. Chemical shifts were calibrated internally against residual solvent signals (DMSO-d_6: δ_C 39.5, δ_H 2.50; $CDCl_3$: δ_H 7.26, δ_C 77.0). ESIMS were recorded using an Agilent 1100 Series separations module equipped with an Agilent 1100 Series LC/MSD mass detector in both positive and negative ion modes. HRESIMS measurements were obtained on a Bruker micrOTOF mass spectrometer. Resin HP-20 (Diaion, Japan), Sephadex LH-20 (GE Healthcare BioSciences AB), and ODS-A (YMC) were used for purification. HPLC was performed using an Agilent 1100 Series HPLC controlled using ChemStation Rev.B.02.01. Energy minimization (MM2) of structures was performed using Chem3D 12.0 (CambridgeSoft).

Collection, cultivation, extraction, isolation and purification

Strain MS100128 was isolated using oatmeal agar from a sediment sample collected in April 2010 from the South China Sea (20° 9.795′ N, 118° 18.124′ E) at 2733 m below sea level and was identified as a *Verrucosispora* sp. using 16S rRNA gene sequence analysis (GenBank accession no. JQ724543). The strain has been preserved at the China General Microbiological Culture Collection Center (accession no. 5847).

Strain MS100128 was cultivated on an ISP2 agar plate (yeast extract 0.4%, malt extract 1%, dextrose 0.4%, agar 2%; pH 7.2) at 28 °C for 7 days. A 250 mL Erlenmeyer flask containing 40 mL of VER01 medium (starch 1%, glucose 1%, glycerol 1%, corn steep powder (Sigma) 0.25%, peptone (Difco) 0.5%, yeast extract 0.2%, NaCl 0.1%, $CaCO_3$ 0.3%; pH 7.0) was inoculated with MS100128 and incubated at 28 °C (220 rpm) for 48 h. Aliquots (9 mL) of the pre-culture were used to inoculate 4 × 1 L Erlenmeyer flasks, each containing 300 mL of VER01 and the flasks were incubated at 28 °C (220 rpm) for 3 days. Aliquots (15 mL) of the seed cultures were aseptically transferred to 70 × 1 L Erlenmeyer flasks, each containing 300 mL of VER01, and the flasks were incubated at 28 °C, 220 rpm for 6 days. The yellow broths were combined and centrifuged to yield a supernatant and a mycelial cake. The mycelial cake was extracted with acetone (3 × 500 mL) and the organic layers were combined with the supernatant and passed through an HP-20 resin column (90 × 7 cm). The column was developed with a 20% stepwise gradient of 0–100% acetone/water, yielding five fractions.

The 40% acetone fraction [MIC (BCG): 50 μg/mL] was evaporated to dryness *in vacuo* and partitioned between H_2O and EtOAc. The EtOAc layer was concentrated *in vacuo* to afford an extract (1.2 g) [MIC (BCG): 6.25 μg/mL], which was fractionated on a Sephadex LH-20 column (50 × 3 cm) in MeOH to give five fractions (Fractions 1–5). Fraction 1 (800 mg) was further fractionated by ODS-MPLC (35 × 2.2 cm) using a gradient elution from 30–80% MeOH/H_2O to provide twelve subfractions (A–L). Fraction H (50 mg) was purified by reversed phase HPLC (Zorbax SB-C_{18} 250 × 9.4 mm, 5 μm column, 2 mL/min, isocratic elution with 45% MeCN/H_2O) to yield abyssomicin B (**4**) (t_R = 9.5 min; 7.4 mg, 0.36%), abyssomicin C (**5**) (t_R = 13.0 min; 8.1 mg, 0.40%), abyssomicin K (**2**) (t_R = 14.8 min; 6.8 mg, 0.33%) and abyssomicin L (**3**) (t_R = 25.3 min; 15.1 mg, 0.74%).[Yields relative to combined mass of 40–80% acetone fraction EtOAc solubles]

The 60% acetone fraction [MIC (BCG): 100 μg/mL] was evaporated to dryness *in vacuo* and then partitioned between H_2O and EtOAc. The EtOAc layer was concentrated *in vacuo* to afford an extract (600 mg) [MIC (BCG): 25 μg/mL], which was fractionated on a Sephadex LH-20 column (50 × 3 cm) in MeOH to give four fractions (Fractions 6–9). Fraction 7 (390 mg) was further fractionated by ODS-MPLC (35 × 2.2 cm) using a gradient elution from 30–80% MeOH/H_2O to provide ten subfractions (M–V). Fraction N contained pure abyssomicin H (**10**) (21.8 mg, 1.1%). Fraction O (28 mg) was purified by reversed phase HPLC (Zorbax SB-C_{18} 250 × 9.4 mm, 5 μm column, 2 mL/min, isocratic elution with 45% MeCN/H_2O) to obtain an additional portion of abyssomicin K (**2**) (t_R = 14.8 min; 10.7 mg, 0.52%). Fraction P (56 mg) was purified by reversed phase HPLC (Zorbax SB-C_{18} 250 × 9.4 mm, 5 μm column, 2 mL/min, isocratic elution with 3:3:4 H_2O/MeCN/MeOH) to obtain abyssomicin D (**6**) (t_R = 6.8 min; 12.8 mg, 0.62%). [Yields relative to combined mass of 40–80% acetone fraction EtOAc solubles]

The 80% acetone fraction [MIC (BCG): 100 μg/mL] was evaporated to dryness *in vacuo* (250 mg) and then successively triturated (5 × 3 mL) with hexane, dichloromethane and MeOH. The dichloromethane-soluble material (98 mg) was then purified by reversed phase HPLC (Zorbax Eclipse XDB-C_8 250 × 9.4 mm, 5 μm column, 3.5 mL/min, gradient elution with 25–100% MeCN/H_2O over 15 min) to yield abyssomicin J (**1**) (t_R = 15.1 min, 23 mg, 1.1%). [Yields relative to combined mass of 40–80% acetone fraction EtOAc solubles]

Structure Elucidation

HRESI(+)MS measurements on abyssomicin J (**1**) revealed a quasi-molecular ion ($[M+Na]^+$) consistent with a molecular formula of $C_{38}H_{46}O_{12}S$ (Δmmu +0.4). Examination of the ^{13}C NMR ($CDCl_3$) data (Table S1) revealed only 19 carbon resonances, requiring a level of symmetry. Further analysis of the 1D NMR data identified resonances for a ketone (δ_C 210.7) and an ester/lactone (δ_C 174.5) carbonyl, two sp^2 hybridized carbons (δ_C 98.2 and 181.3), as well as two oxymethines (δ_H 4.50, δ_C 69.4; δ_H 3.76, δ_C 76.5), one thiomethine (δ_H 3.83, δ_C 41.1), two quaternary oxycarbons (δ_C 85.3 and 87.2) and a chelated OH (δ_H 11.30). These observations require involvement of six O, one shared S, and three double bond equivalents per symmetric unit, necessitating that each unit be pentacyclic and linked via a thioether bridge. COSY correlations from H-4 to H-6 inclusive of H_3-18 and H_3-19, and from H-8 to H_2-14 inclusive of H_3-17, defined structure fragments A and B respectively (Figure S1e). HMBC correlations from H_2-14 to C-16, C-15 and C-10, and from H-10 to C-16, C-15 and C-14, refined fragment B to include a C-10 to C-15 carbocycle extended to C-16, while correlations from H-12 to C-16 inserted the C-12 to C-16 oxy bridge. HMBC correlations from H-4 to C-2 and C-3, and from the 3-OH to C-3 and C-4, extended fragment A to C-2 as indicated, inclusive of a $\Delta^{2,3}$ enol moiety, while correlations from H-6 and H-8 to C-7, and from H-6 to C-8, linked fragments A and B. A stable $\Delta^{2,3}$ enol residue, together with a chelated 3-OH NMR resonance (δ_H 11.30), was indicative of a C-1 lactone and a $\Delta^{2,3}$ *Z* configuration, and mandated closure of the lactone ring to C-15 to generate a spiro-heterocycle, and closure from C-8 to C-16 to generate the carbocyclic framework common to D (**6**) and E (**7**). Finally, an apparent HMBC correlation from H-9 (δ_H 3.83) to C-9 (δ_C 41.1) was evidence of dimerization through a C-9 to C-9′ thioether bridge. ROESY correlations between H-5b, H-8, H-11, H_3-18 and H_3-19 necessitated that all these residues reside on a common (β) face (Figure S1e), while the highly fused and rigid nature of the carbo/hetero cyclic systems in **1** allowed extension of this relative configuration to include C-10, C-15 and C-16. A C-13 2° methyl pseudo-equatorial configuration could be assigned on the basis of NMR ($CDCl_3$) comparisons between **1** (δ_C 18.8, C-17; δ_H 2.62, H-13; δ_H 1.03, H_3-17) and E (**7**) (δ_C 18.6, C-17; δ_H 2.50, H-13; δ_H 1.00, H_3-17),{Niu, 2007 #4} while the C-9 β thioether configuration was assigned by comparing experimental data for H-9 ($J_{8,9}$ 10.8 Hz; $J_{9,10}$ 3.6 Hz) with calculated values for energy minimized (MM2) *in silico* models of α ($J_{8,9}$ 6–7 Hz; $J_{9,10}$ <1 Hz) and β ($J_{8,9}$ 7–8 Hz; $J_{9,10}$ 3–4 Hz) thioethers, and with the literature data for E (**7**) ($J_{8,9}$ 8 Hz; $J_{9,10}$ 4 Hz).{Niu, 2007 #4}

HRESIMS measurements on abyssomicins K (**2**) and L (**3**) revealed quasi-molecular ions consistent with molecular formulae (**2**, $C_{19}H_{24}O_7$, Δmmu +0.8; **3**, $C_{20}H_{26}O_7$, Δmmu +0.5) attributed to H_2O and MeOH Michael addition adducts of abyssomicin C (**5**), respectively. In support of this hypothesis, the NMR ($CDCl_3$) data for **2** and **3** (Tables S2 and S3) proved to be very similar to those of **1**, with significant differences being limited to replacement of the thiomethine in **1** (δ_H 3.83; δ_C 41.1) with a hydroxymethine in **2** (δ_H 4.81 and δ_C 67.7), and a methoxymethine in **3** (δ_H 4.43 and δ_C 76.7; OMe δ_H 3.30 and δ_C 58.2). The 2D NMR data for **2** and **3** also revealed diagnostic correlations supportive of the proposed structures (Figures S2e and S3e).

Characterisation of Compounds

Abyssomicin J (1): white powder; $[\alpha]^{24}_{D}$ +188 (*c* 0.05, MeOH); UV (MeOH) λ_{max} (log ε) 296 (3.83), 267 (4.13), 202 (4.01) nm; NMR data, see Table S1; HRESIMS *m/z* 749.2606 $[M+Na]^+$ (calcd. for $C_{38}H_{46}O_{12}SNa$, 749.2602).

Abyssomicin K (2): pale white powder; $[\alpha]^{24}_{D}$ +48 (*c* 0.04, MeOH); UV (MeOH) λ_{max} (log ε) 296 (3.32), 264 (3.71), 202 (3.60) nm; NMR data, see Table S2; HRESIMS *m/z* 365.1603 $[M+H]^+$ (calcd. for $C_{19}H_{25}O_7$, 365.1595).

Abyssomicin L (3): white powder; $[\alpha]^{24}_{D}$ +101 (*c* 0.05, MeOH); UV (MeOH) λ_{max} (log ε) 296 (3.40), 265 (3.74), 204 (3.54) nm; NMR data, see Table S3; HRESIMS *m/z* 377.1611 $[M-H]^-$ (calcd. for $C_{20}H_{25}O_7$, 377.1606).

Abyssomicin B (4): white powder; $[\alpha]^{24}_{D}$ –68 (*c* 0.05, MeOH); NMR data, see Table S4; ESIMS *m/z* 378.1 $[M+H]^+$.

Abyssomicin C (5): white powder; $[\alpha]^{24}_{D}$ –20 (*c* 0.05, MeOH); NMR data, see Table S5; ESIMS *m/z* 347.0 $[M+H]^+$.

Abyssomicin D (6): white powder; $[\alpha]^{24}_{D}$ +121 (*c* 0.05, MeOH); NMR data, see Table S6; ESIMS *m/z* 349.2 $[M+H]^+$.

***Atrop*-abyssomicin C (8)**: NMR data, see Table S7. HRESIMS *m/z* 369.1305 $[M+Na]^+$ (calcd. for $C_{19}H_{22}O_6Na$, 369.1309).

Abyssomicin H (10): white powder; $[\alpha]^{24}_{D}$ –18 (*c* 0.05, MeOH); NMR data, see Table S8; ESIMS *m/z* 349.2 $[M+H]^+$.

Abyssomicin thiol (12): HRESIMS *m/z* 403.1184 $[M+Na]^+$ (calcd. for $C_{19}H_{24}O_6SNa$, 403.1186).

Abyssomicin sulfinic acid (13): HRESIMS *m/z* 411.1112 $[M-H]^-$ (calcd. for $C_{19}H_{23}O_8S$, 411.1119).

Abyssomicin sulfonic acid (14): NMR data, see Figure S9. HRESIMS *m/z* 427.1074 $[M-H]^-$ (calcd. for $C_{19}H_{23}O_9S$, 427.1068).

Abyssomicin 15: NMR data, see Table S10. HRESIMS *m/z* 365.1598 $[M+H]^+$ (calcd. for $C_{19}H_{25}O_7$, 365.1595).

Abyssomicin sulfinic acid isomer (16): HRESIMS *m/z* 411.1118 $[M-H]^-$ (calcd. for $C_{19}H_{23}O_8S$, 411.1119).

Abyssomicin sulfonic acid isomer (17): HRESIMS *m/z* 427.1071 $[M-H]^-$ (calcd. for $C_{19}H_{23}O_9S$, 427.1068).

Abyssomicin sulfoxide (18): HRESIMS *m/z* 765.2552 $[M+Na]^+$ (calcd. for $C_{38}H_{46}O_{13}SNa$, 765.2551).

Abyssomicin sulfone (19): ^{1}H NMR, see Figure S11a. HRESIMS *m/z* 757.2541 $[M-H]^-$ (calcd. for $C_{38}H_{46}O_{14}S$, 757.2536).

Bioassays

General Antimicrobial Assays: Antimicrobial assays were performed according to the Antimicrobial Susceptibility Testing Standards outlined by the Clinical and Laboratory Standards Institute (CLSI) using the bacteria *Staphylococcus aureus* (ATCC 6538), *Pseudomonas aeruginosa* (PAO1) and *Bacillus subtilis* (ATCC 6633). For each organism, a loopful of glycerol stock was streaked on an LB-agar plate, which was incubated overnight at 37 °C. A single bacterial colony was picked and suspended in Mueller-Hinton Broth to approximately 1×10^4 cfu/mL. A two-fold serial dilution of each compound to be tested (4000 to 31.3 μg/mL in DMSO) was prepared and an aliquot of each dilution (2 μL) was added to a 96-well flat-bottom microtiter plate (Greiner). Vancomycin and ciprofloxacin were used as positive controls and DMSO as the negative control. An aliquot (78 μL) of bacterial suspension was then added to each well (to give final compound concentrations of 100 to 0.78 μg/mL in 2.5% DMSO) and the plate was incubated at 37 °C aerobically for 16 h. Finally, the optical density of each well at 600 nm was measured with an EnVision 2103 Multilabel Plate Reader (Perkin-Elmer Life Sciences). MIC values were defined as the minimum concentration of compound that inhibited visible bacterial growth. All the experiments were performed in triplicate.

Anti-BGC Assay: The Bacille Calmette–Guérin (BCG) antimicrobial assay was performed with a strain of *Mycobacterium bovis* (BCG Pasteur 1173P2) containing a constitutive Green Fluorescent Protein (GFP) expression vector (pUV3583c-GFP) using direct readout of fluorescence as a measure of bacterial growth, as described previously.[1] The BCG strain was pre-cultivated to mid-log phase (7 d) at 37 °C in Middlebrook 7H9 broth (40 mL; Difco) supplemented with 10% OADC enrichment (Becton Dickinson), 0.05% Tween-80 and 0.2% glycerol and then diluted to an OD_{600} of 0.025 with broth. A two-fold serial dilution of each compound to be tested (4000 to 31.3 μg/mL in DMSO) was prepared and an aliquot of each dilution (2 μL) was added to a 96-well flat-bottom microtiter plate (Greiner). Isoniazid was used as the positive control and DMSO as the negative control. An aliquot (78 μL) of the BCG-GFP bacterial suspension was then added to each well (to give final compound concentrations of 100 to 0.78 μg/mL in 2.5% DMSO) and the plate was incubated at 37 °C for 3 d. Mycobacterial growth was determined by measuring GFP-fluorescence using an EnVision 2103 Multilabel Plate Reader (Perkin-Elmer Life Sciences) with excitation at 485 nm and emission at 535 nm. All the experiments were performed in triplicate. MIC was defined as the minimum concentration that inhibited more than 90% of bacteria growth.

Anti-MTB assay: The *in vitro* activity of compounds against *Mycobacterium tuberculosis* H37Rv (ATCC27294) was determined in 96-well plate format as described previously.[2] H37Rv were grown at 37 °C to mid log phase in Middlebrook 7H9 broth supplemented with 10% OADC enrichment (Becton Dickinson), 0.05% Tween-80, and 0.2% glycerol. The culture was then diluted with culture medium to a bacterial suspension with OD_{600} values of 0.6-0.8. The culture was diluted with 7H9 medium to an OD_{600} of 0.01, and 100 μL was added to a microplate containing twofold serial dilutions of the tested compounds for a final volume of 200 μL. Isoniazid was used as the positive control while DMSO as the negative control. The plates were incubated at 37 °C for 10 days. Growth of the bacteria was recorded by spectrophotometer reading at OD_{600}. MIC was defined as the minimum concentration of drug that inhibits more than 90% of bacterial growth. After 10 days, 10 μL of alamarBlue solution (Invitrogen) was added to each well of the microplates and the color of each well was recorded after 48 h. A blue color in the well was interpreted as no bacterial growth, and a pink color was interpreted as growth.

Antifungal Assays: Antifungal and bioassays were performed according to a previously described[3] protocol modified from the Clinical and Laboratory Standards Institute M-27A methods[4] using the fungus *Candida albicans* (SC 5314). A colony of *Candida albicans* was picked from a YPD agar plate and suspended in RPMI 1640 to a concentration of 1×10^4 cfu/mL. A two-fold serial dilution of each compound to be tested (4000 to 31.3 μg/mL in DMSO) was prepared and an aliquot of each dilution (2 μL) was added to a flat-bottom, 96-well microtiter plate (Greiner). Ketoconazole was used as the positive control and DMSO as the negative control. An aliquot (78 μL) of the fungal suspension was added to each well (to give final compound concentrations of 100 to 0.78 μg/mL in 2.5% DMSO) and the plate was incubated at 35 °C for 16 h. Finally, the optical density of each well at 600 nm was measured with a EnVision 2103 Multilabel Plate Reader (Perkin-Elmer Life Sciences) and the antifungal MICs were defined as the minimum concentration of compound that inhibited visible fungal growth.

[1] Wang, J.F., Dai, H.Q., Wei, Y.L., Zhu, H.J., Yan, Y.M., Wang, Y.H., Long, C.L., Zhong, H.M., Zhang, L.X. "Antituberculosis agents and an inhibitor of the para-aminobenzoic acid biosynthetic pathway from *Hydnocarpus anthelminthica* seeds." *Chem. Biodivers*. **2010**, *7*, 2046–2053.

[2] Brown, J.R, North, E.J., Hurdle, J.G., Morisseau, C., Scarborough, J.S., Sun, D., Korduláková, J., Scherman, M.S., Jones, V., Grzegorzewicz, A., Crew, R.M., Jackson, M., McNeil, M.R., Lee, R.E. The structure-activity relationship of urea derivatives as anti-tuberculosis agents. *Bioorg Med Chem.* **2011**, *19*, 5585–95.

[3] Zhang, L. X. *et al.* "High-throughput synergy screening identifies microbial metabolites as combination agents for the treatment of fungal infections." *Proc. Natl. Acad. Sci. USA*. **2007**, *104*, 4606–4611.

[4] National Committee for Clinical Laboratory Standards. Reference method for broth dilution antifungal susceptibility testing of yeasts. Approved Standard-Second Edition M27-A2. Pennsylvania, USA 2002.

Biotransformation of abyssomicin J by BCG

BCG (strain Pasteur 1173P2) was cultivated to mid-log phase (7 d; OD_{600} 0.2) at 37 °C in Middlebrook 7H9 broth (40 mL; Difco) supplemented with 10% OADC enrichment (Becton Dickinson), 0.05% Tween-80 and 0.2% glycerol. An aliquot (20 μL) of abyssomicin J (**1**; 4 mg/mL in DMSO) was added to an aliquot (1 mL) of BCG culture and the suspension was incubated at 37 °C for 1 h. The suspension was then diluted with MeCN (50 μL), centrifuged and the supernatant analyzed by HPLC-DAD-HRESI(+)MS.

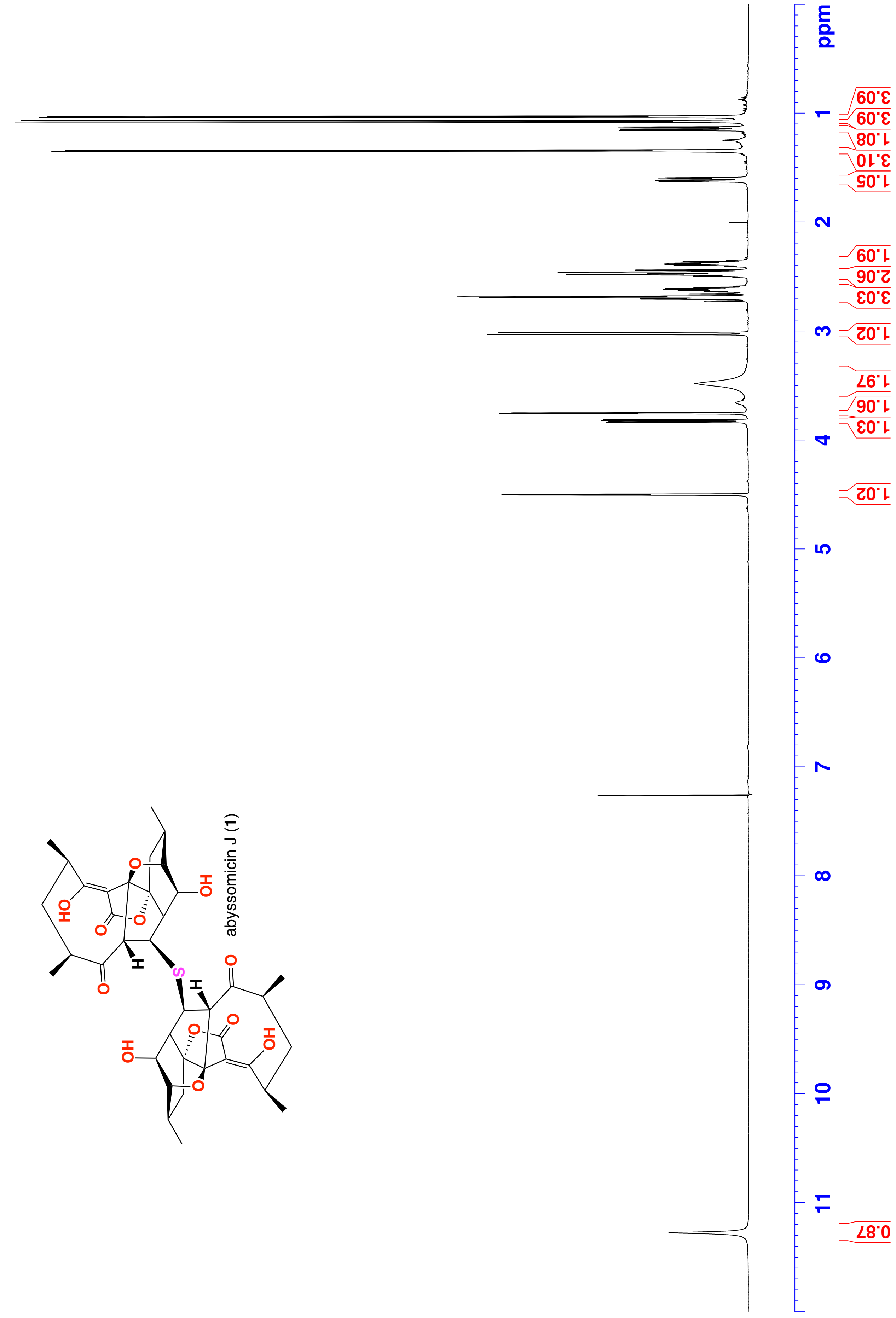

Figure S1a. ^{1}H NMR spectrum (600 MHz, $CDCl_3$) of abyssomicin J (**1**)

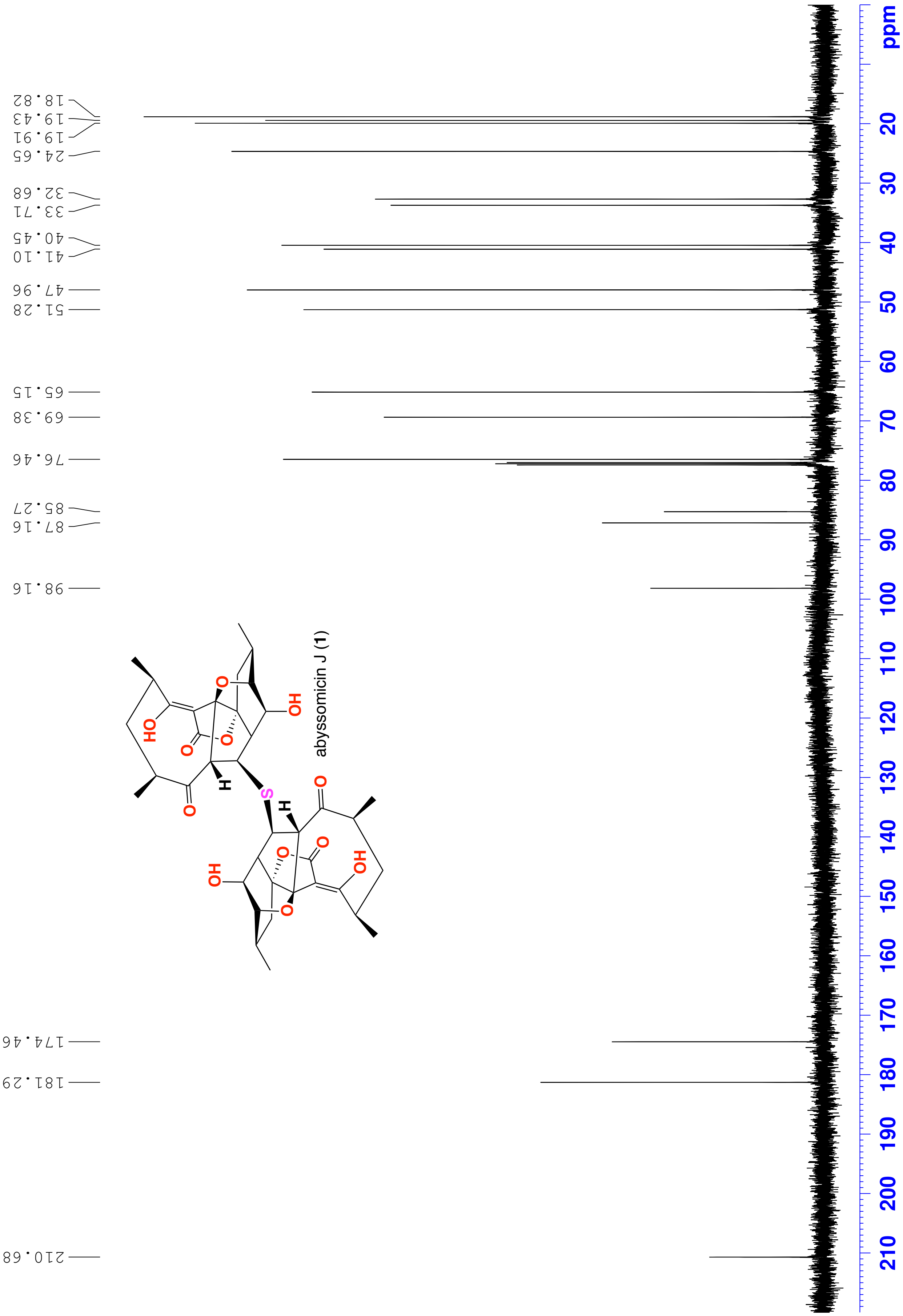

Figure S1b. ^{13}C NMR spectrum (150 MHz, $CDCl_3$) of abyssomicin J (**1**)

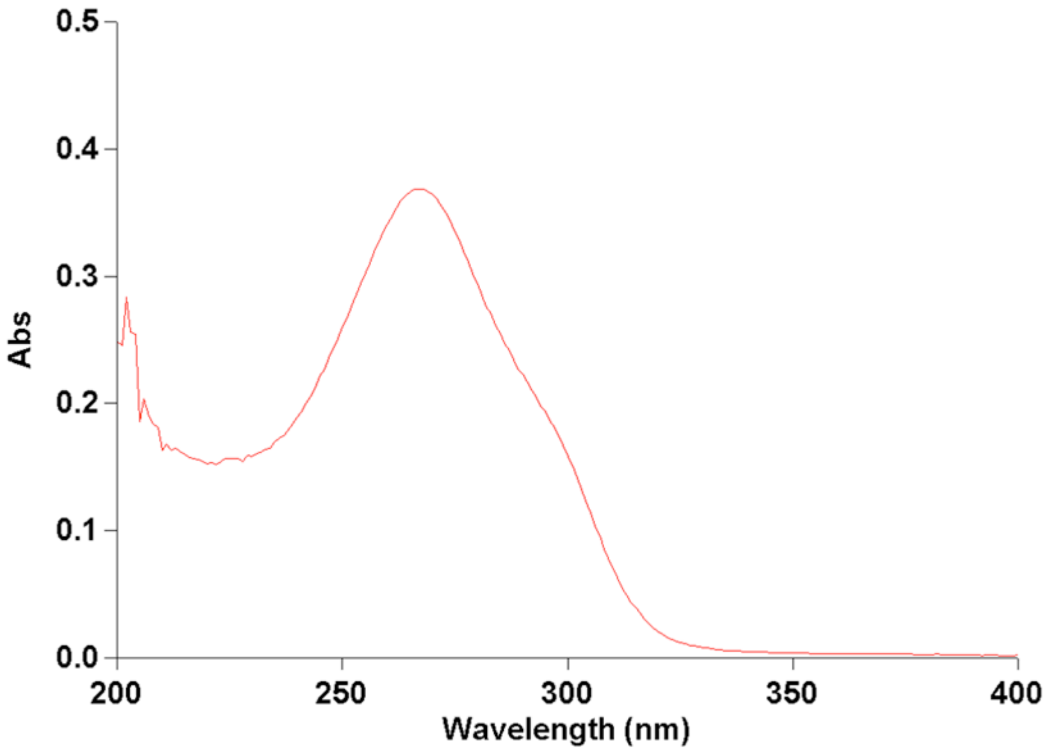

Figure S1c. UV-vis spectrum of abyssomicin J (**1**)

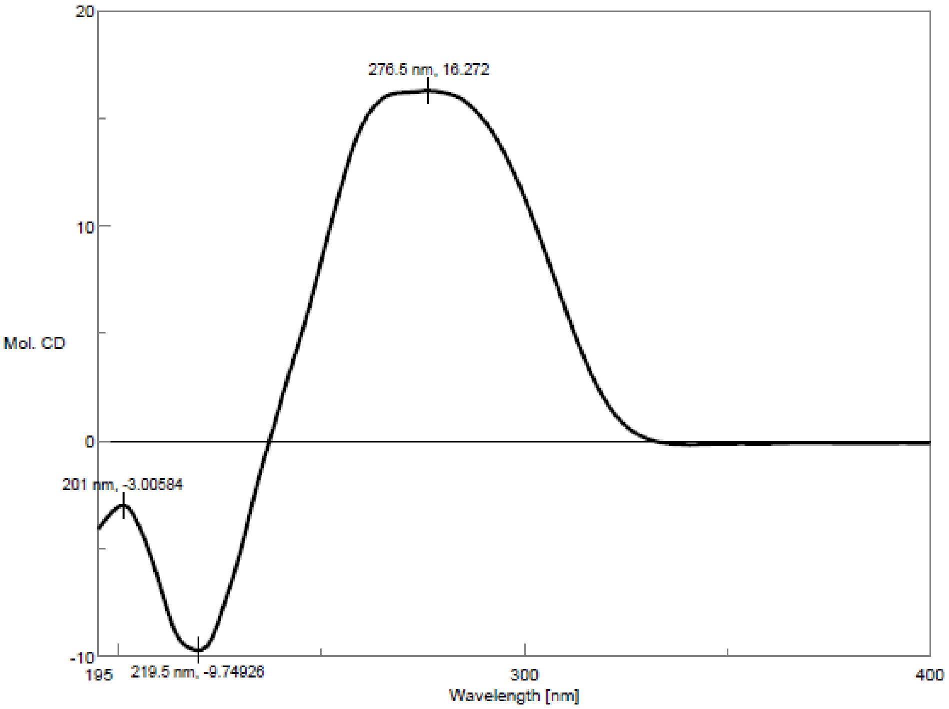

Figure S1d. CD spectrum of abyssomicin J (**1**) in MeOH

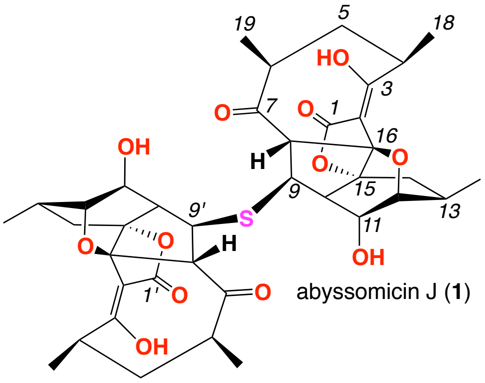

Table S1. NMR data (600 MHz, $CDCl_3$) for abyssomicin J (**1**)

Pos.	δ_H, mult (*J* in Hz)	δ_C	COSY	^{1}H-^{13}C HMBC	ROESY
1		174.5			
2		98.2			
3		181.3			
4	2.49, m	40.5	5a/b, 18	2, 3, 5, 18	
5a	2.70, m	33.7	4, 5b, 6	4, 6, 7, 18	
5b	1.61, dd (*15.6, 6.0*)		5a, 6	3, 4, 6, 7, 19	19
6	2.38, m	48.0	5a/b, 19	4, 5, 7, 8, 19	
7		210.7			
8	3.02, d (*10.8*)	65.1	9	2, 7, 9	11
9	3.83, dd (*10.8, 3.6*)	41.1	8, 10	7, 8, 9′, 10, 11	
10	2.68, m	51.3	9, 11	9, 11, 15, 16	11
11	4.50, d (*4.2*)	69.4	10, 12	9, 12, 13, 15	8, 10
12	3.76, d (*4.2*)	76.5	11, 13	10, 11, 14, 16, 17	13, 17
13	2.62, m	24.6	12, 14a/b, 17	14, 17	12
14a	2.45, m	32.7	13, 14b	10, 12, 13, 15, 16	
14b	1.14, dd (*13.8, 6.0*)		13, 14a	10, 12, 13, 15, 16, 17	
15		87.2			
16		85.3			
17	1.03, d (*7.2*)	18.8	13	12, 13, 14	12
18	1.35, d (*7.2*)	19.9	4	3, 4, 5	
19	1.08, d (*7.2*)	19.4	6	5, 6, 7	8
3-OH	11.28, s			3, 4	

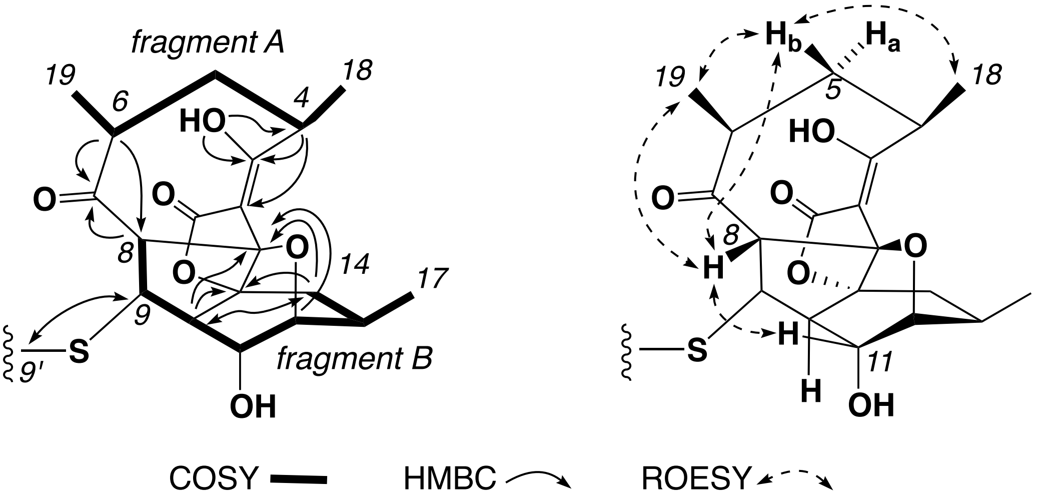

Figure S1e. Selected 2D NMR correlations for abyssomicin J (**1**)

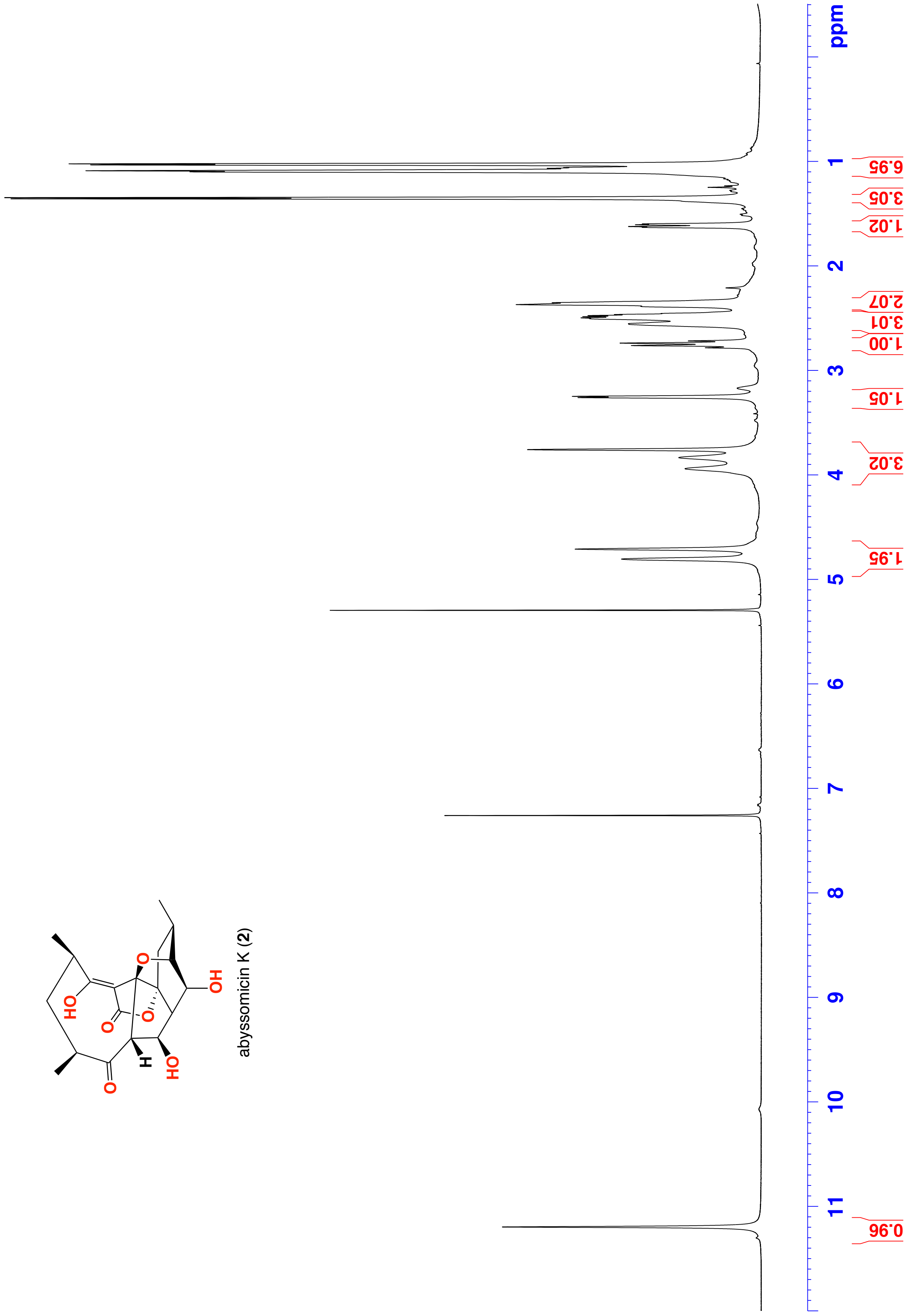

Figure S2a. ^{1}H NMR spectrum (600 MHz, $CDCl_3$) of abyssomicin K (**2**)

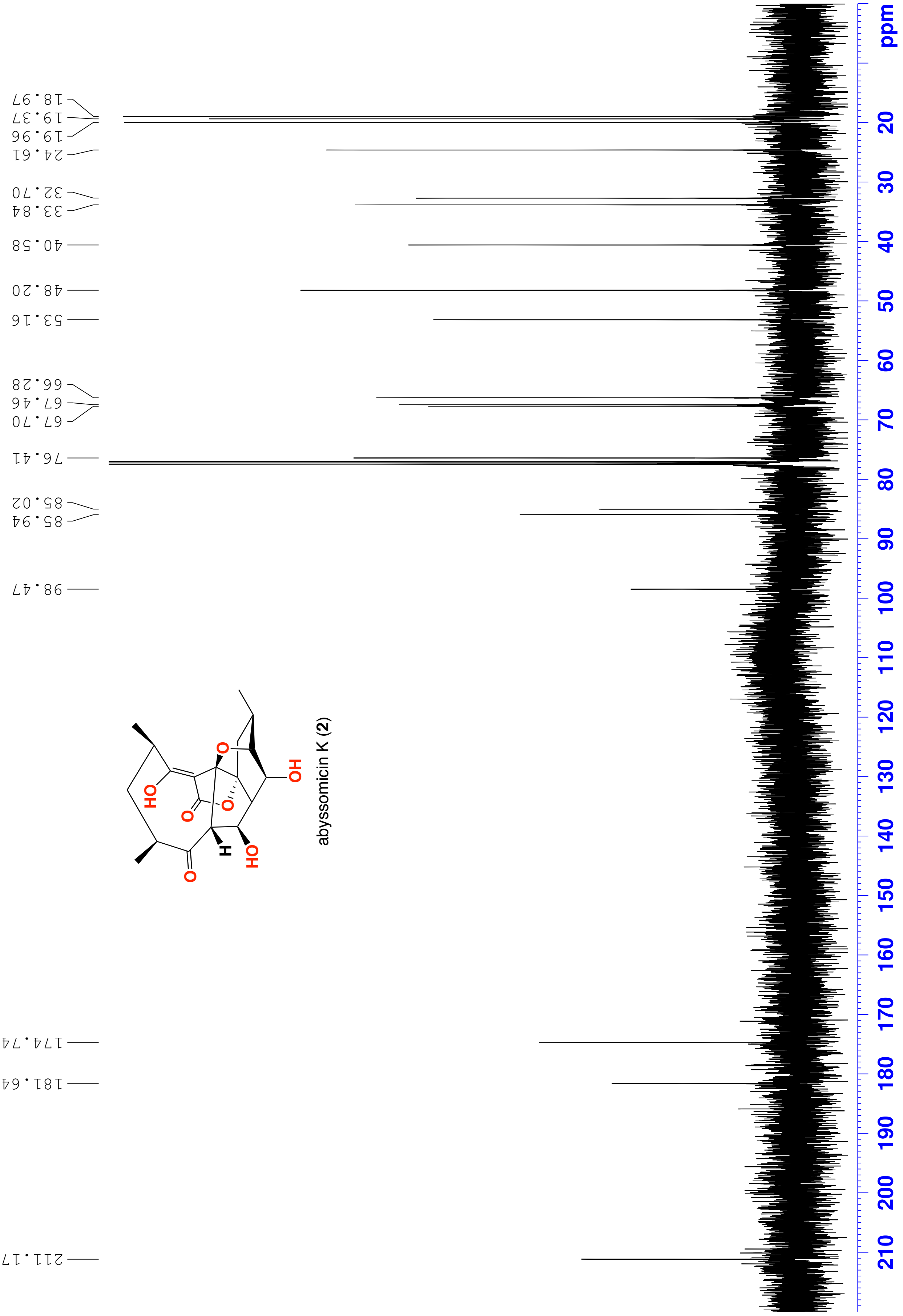

Figure S2b. ^{13}C NMR spectrum (150 MHz, $CDCl_3$) of abyssomicin K (**2**)

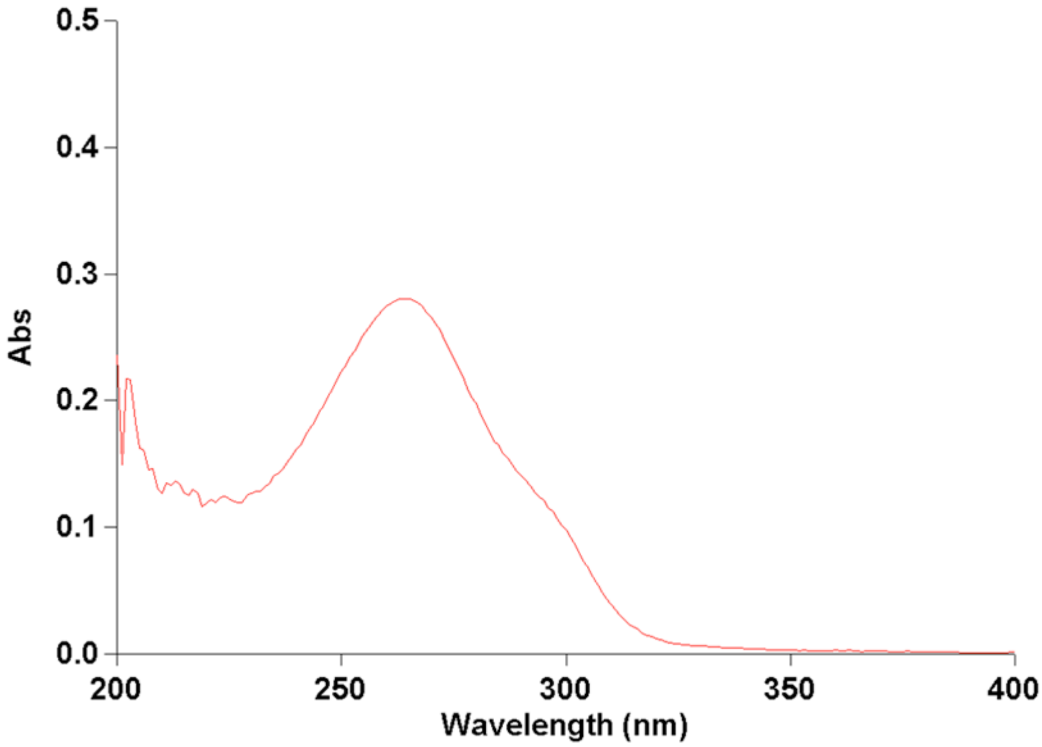

Figure S2c. UV-vis spectrum of abyssomicin K (**2**)

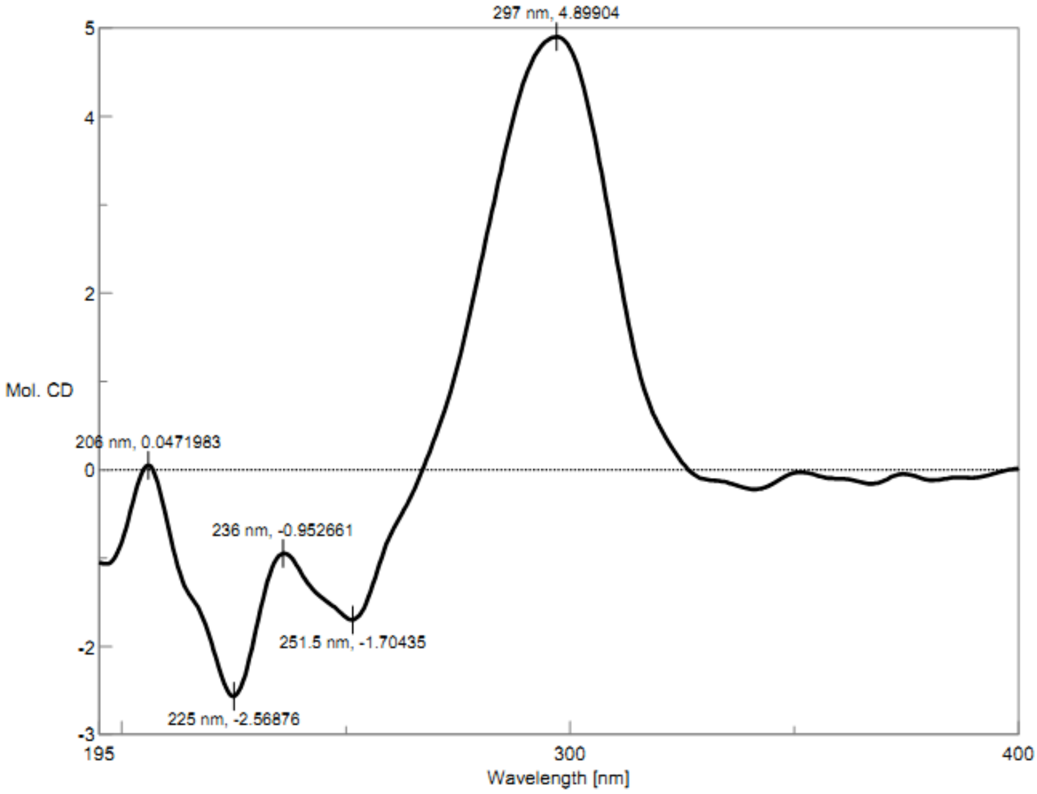

Figure S2d. CD spectrum of abyssomicin K (**2**) in MeOH

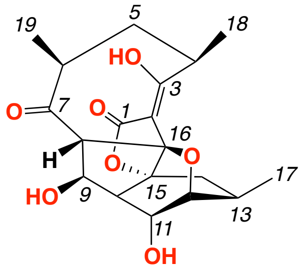

abyssomicin K (**2**)

Table S2. NMR data (600 MHz, $CDCl_3$) for abyssomicin K (**2**)

Pos.	δ_H, mult (*J* in Hz)	δ_C	COSY	^1H-^{13}C HMBC	ROESY
1		174.7			
2		98.5			
3		181.6			
4	2.48, m	40.7	5a/b, 18		3-OH
5a	2.75, m	33.8	4, 5b, 6	4, 6, 7, 18, 19	
5b	1.61, dd (*15.6, 6.0*)		4, 5a	3, 4, 6, 7, 19	6, 19
6	2.36, m	48.2	5a, 19	4, 5, 7, 8, 19	5b
7		211.2			
8	3.25, d (*6.6*)	66.3	9	7, 9, 16	5, 9, 11, 19
9	4.81, br s	67.7	8, 10	8, 11	8
10	2.51, m	53.2	9		
11	4.71, br s	67.6	12	9, 13, 15	8, 12
12	3.76, br s	76.4	11	10, 14, 16	11, 13, 17
13	2.56, m	24.6	14a/b, 17		12
14a	2.37, m	32.7	13, 14b	15, 16	
14b	1.07, m		13, 14a	10, 13, 15, 16	
15		85.9			
16		85.0			
17	1.03, d (*7.2*)	19.0	13	12, 13, 14	12
18	1.35, d (*7.2*)	20.0	4	3, 4, 5	5b
19	1.08, d (*7.2*)	19.4	6	5, 6, 7	5a/b, 8
3-OH	11.20, s			2, 3, 4	4

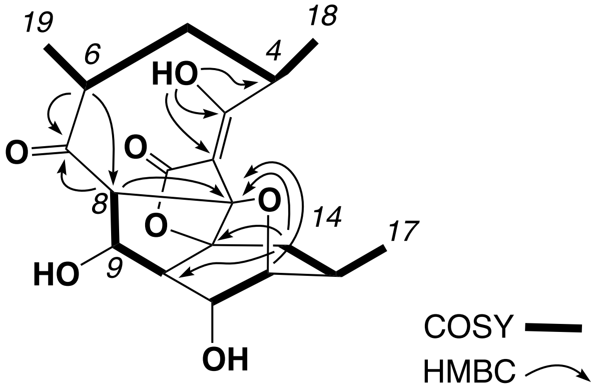

abyssomicin K (**2**)

Figure S2e. Selected 2D NMR correlations for abyssomicin K (**2**)

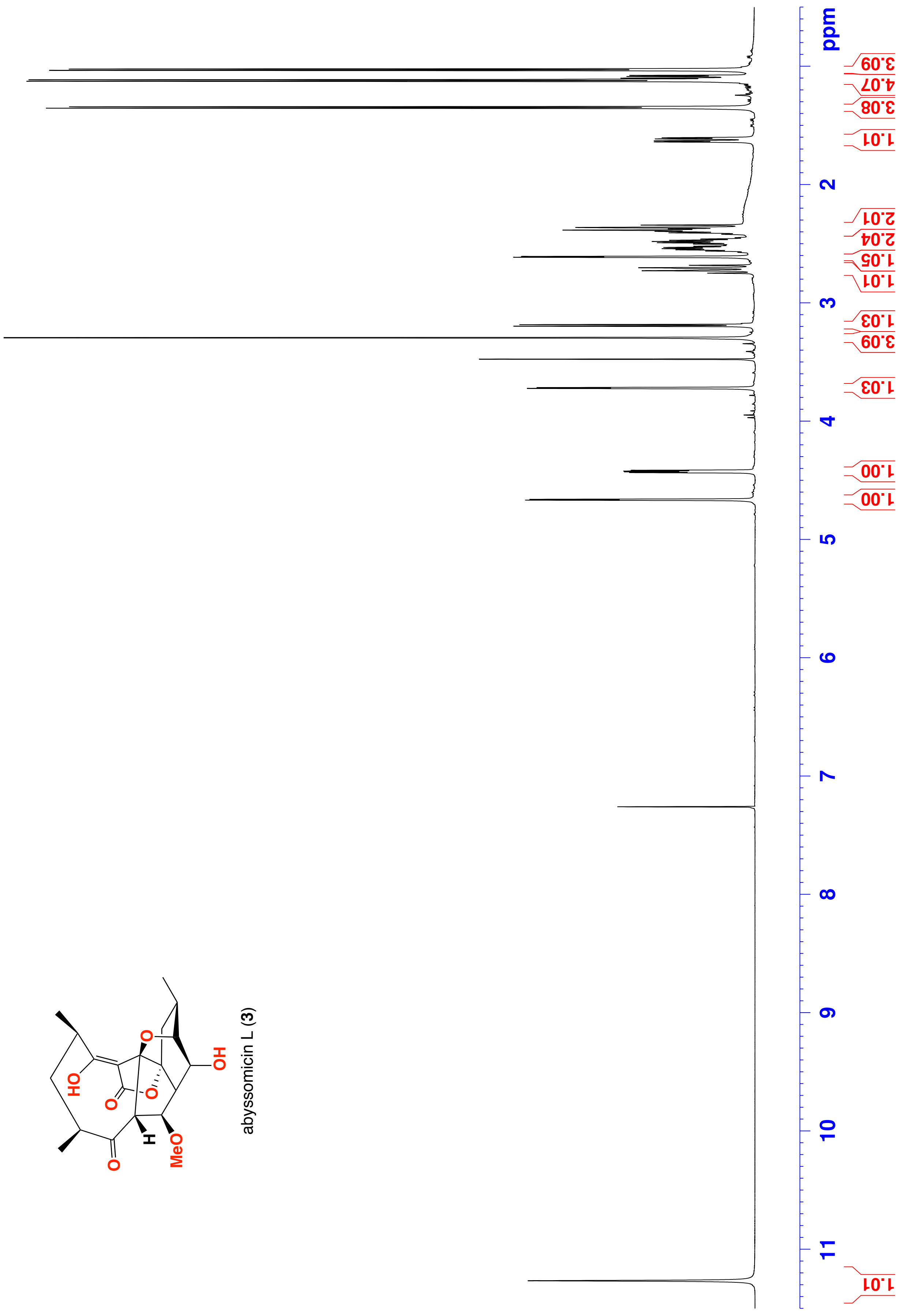

Figure S3a. ^{1}H NMR spectrum (600 MHz, $CDCl_3$) of abyssomicin L (**3**)

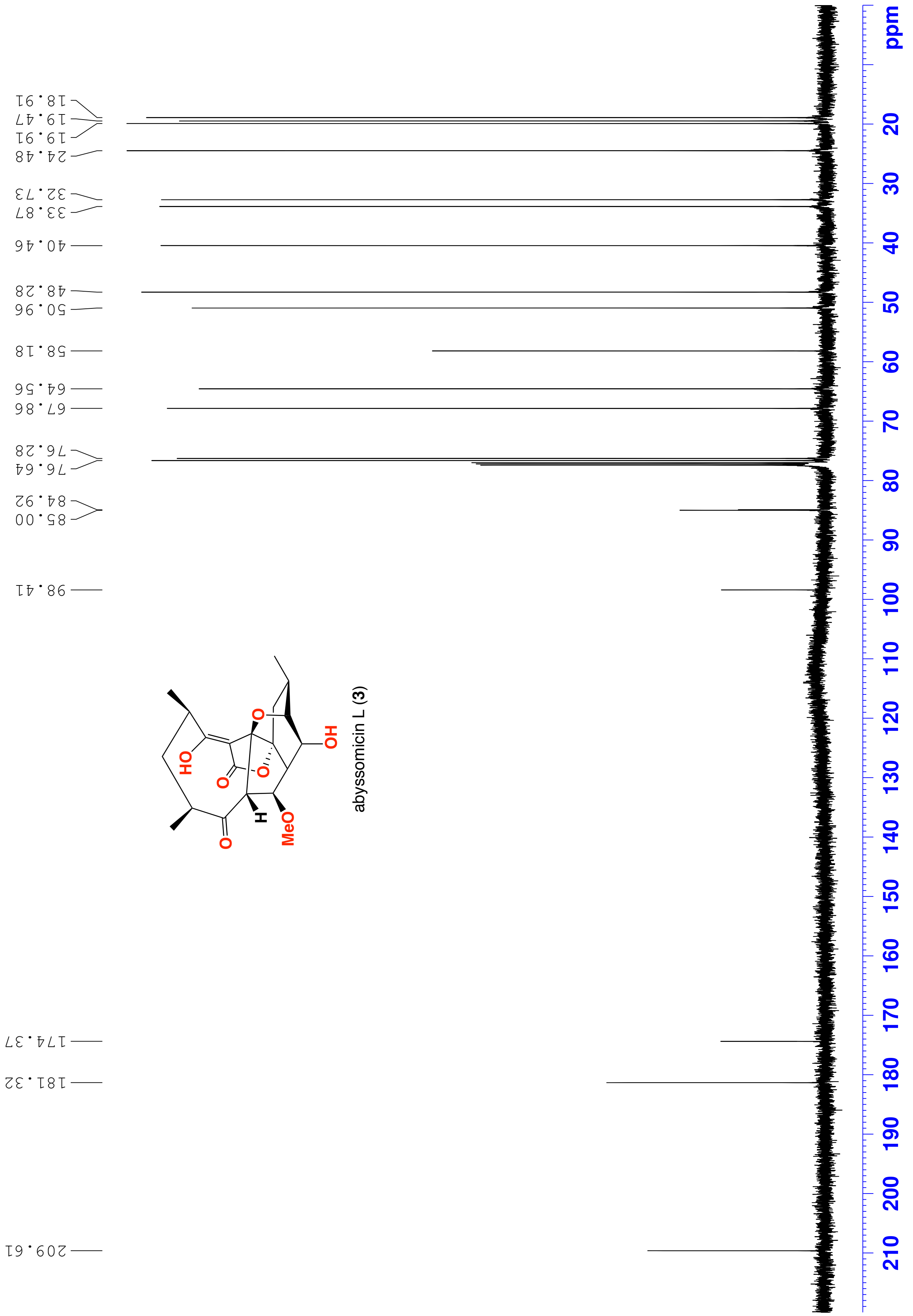

Figure S3b. ^{13}C NMR spectrum (150 MHz, $CDCl_3$) of abyssomicin L (**3**)

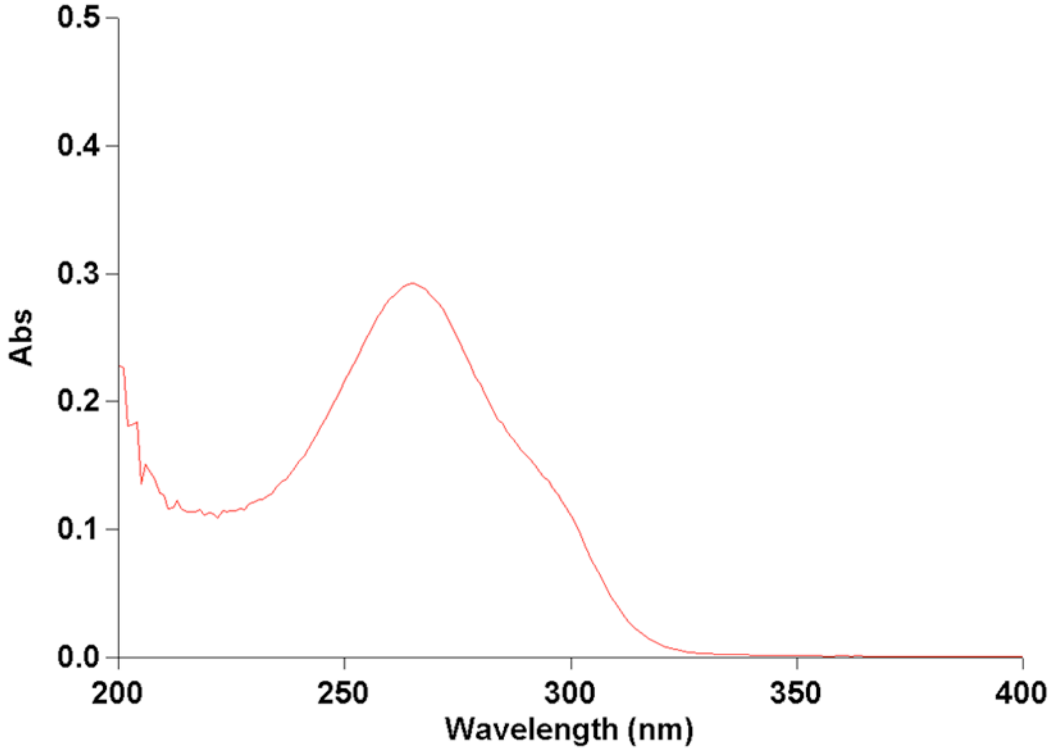

Figure S3c. UV-vis spectrum of abyssomicin L (**3**)

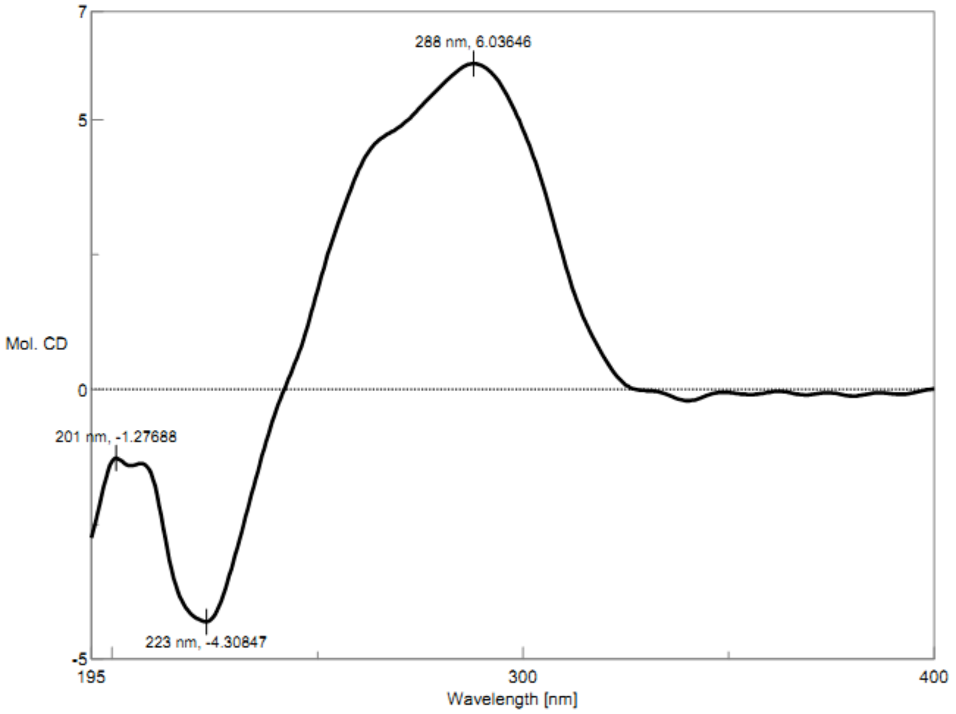

Figure S3d. CD spectrum of abyssomicin L (**3**) in MeOH

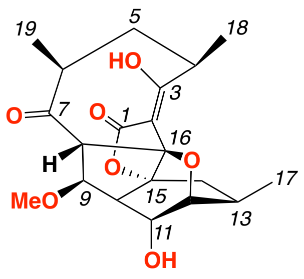

abyssomicin L (**3**)

Table S3. NMR data (600 MHz, $CDCl_3$) for abyssomicin L (**3**)

Pos.	δ_H, mult (*J* in Hz)	δ_C	COSY	^{1}H-^{13}C HMBC	ROESY
1		174.4			
2		98.4			
3		181.3			
4	2.49, m	40.5	5a/b, 18	2, 3, 5, 18	
5a	2.72, m	33.9	4, 5b, 6	4, 6, 7, 18	8, 17, 19
5b	1.62, dd (*14.4, 5.4*)		4, 5a	3, 4, 6, 7, 19	17, 19
6	2.40, m	48.3	5a, 19	4, 5, 7, 8, 19	5b
7		209.6			
8	3.19, d (*7.8*)	64.6	9	7, 9, 15, 16	5a, 11, 12
9	4.43, dd (*7.8, 4.2*)	76.7	8, 10	8, 11, 9-OMe	9-OMe
10	2.61, d (*4.2*)	51.0	9	8, 11, 16	11, 9-OMe
11	4.67, d (*4.2*)	67.9	12	9, 13, 15	8, 10, 12
12	3.72, d (*4.2*)	76.3	11	10, 14, 16, 17	11, 13, 17
13	2.54, m	24.5	14a/b, 17	17	12
14a	2.38, m	32.7	13, 14b	10, 12, 16	
14b	1.09, dd (*13.8, 5.4*)		13, 14a	10, 13, 15, 16, 17	
15		85.0			
16		84.9			
17	1.02, d (*7.2*)	18.9	13	12, 13, 14	12
18	1.35, d (*7.2*)	19.9	4	3, 4, 5	5a
19	1.12, d (*7.2*)	19.5	6	5, 6, 7	5a/b, 8, 9-OMe
3-OH	11.27, s			2, 3, 4	
9-OMe	3.30, s	58.2		9	9, 10, 11, 19

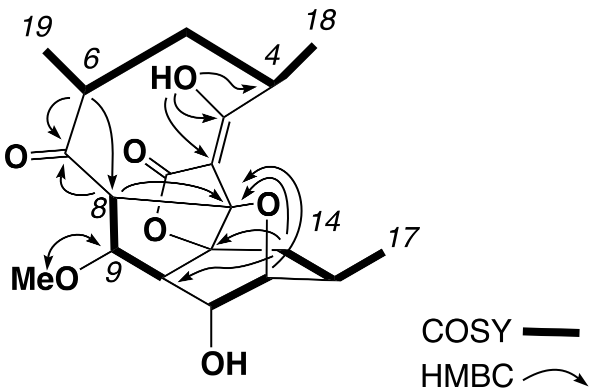

abyssomicin L (**3**)

Figure S3e. Selected 2D NMR correlations for abyssomicin L (**3**)

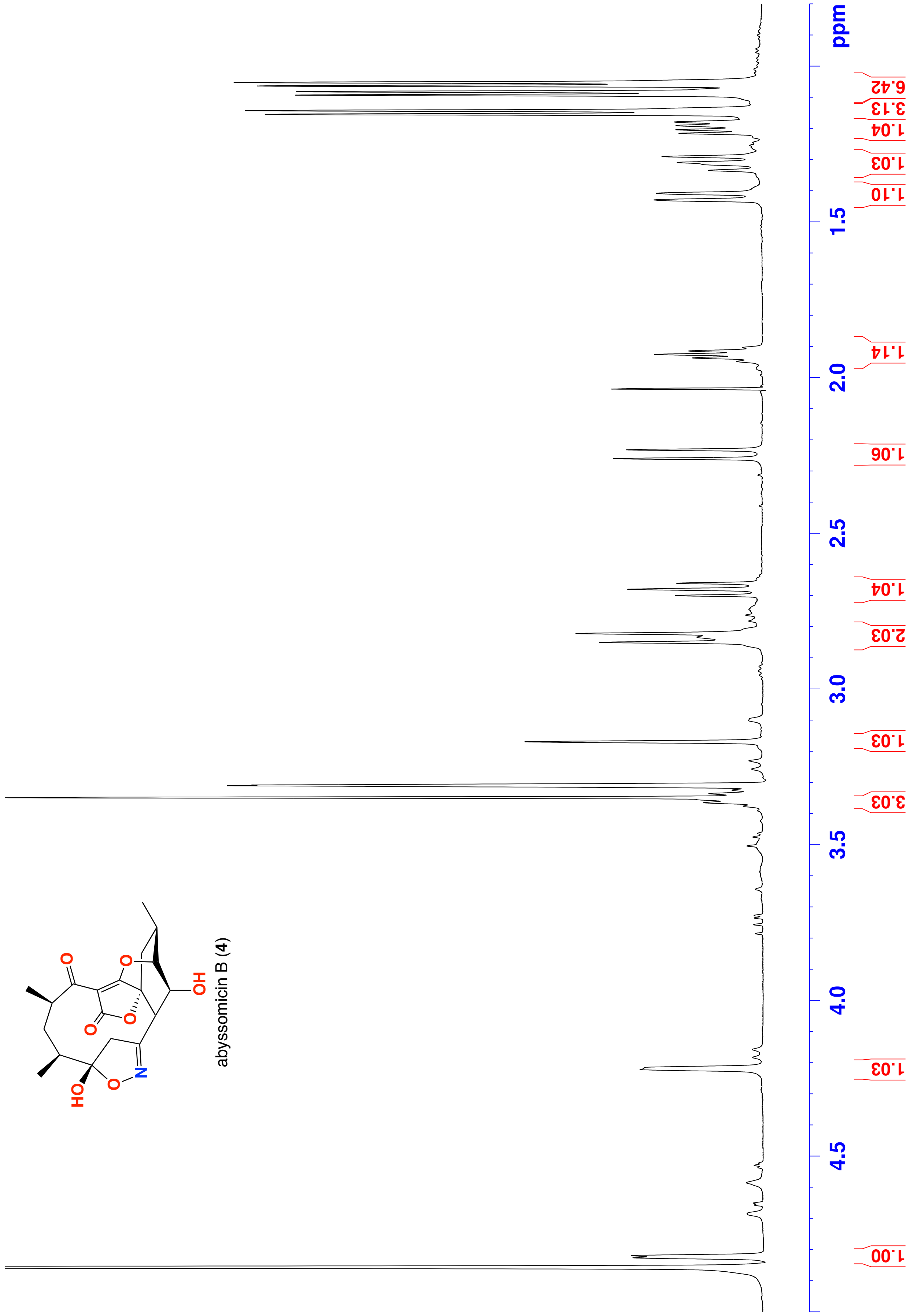

Figure S4a. ^{1}H NMR spectrum (600 MHz, methanol-d_4) of abyssomicin B (**4**)

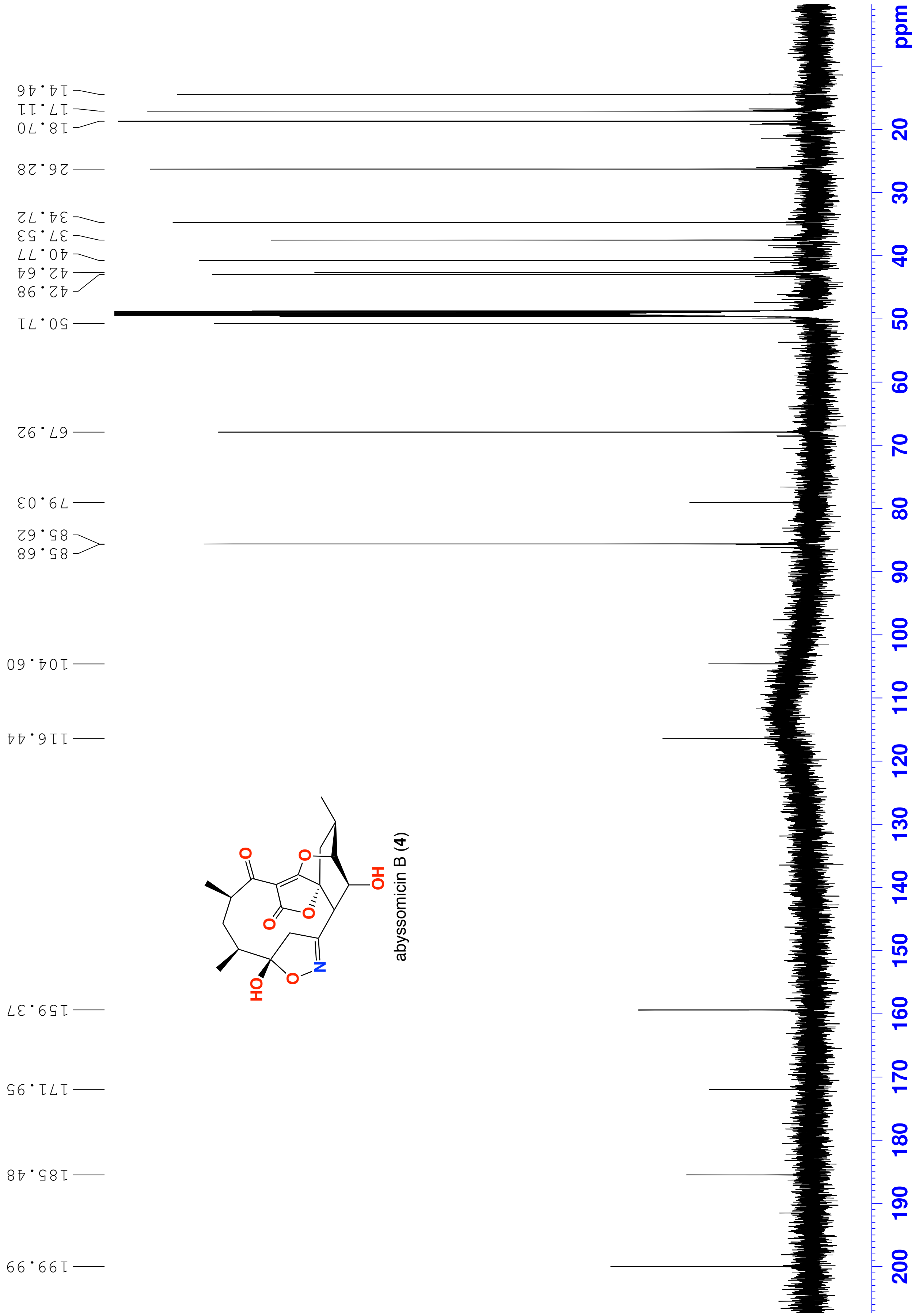

Figure S4b. ^{13}C NMR spectrum (150 MHz, methanol-d_4) of abyssomicin B (**4**)

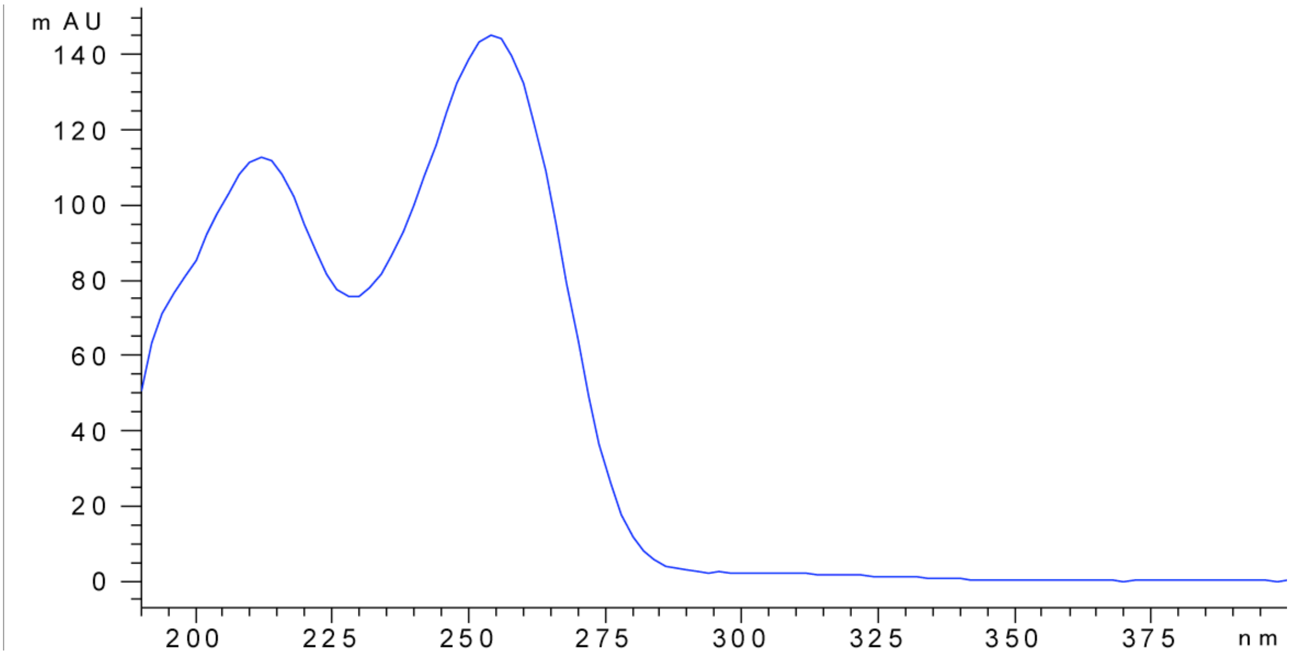

Figure S4c. UV-vis spectrum of abyssomicin B (**4**)

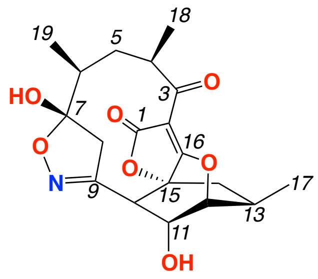

abyssomicin B (**4**)

Table S4. NMR data (600 MHz, methanol-d_4) for abyssomicin B (**4**)

Pos.	δ_H, mult (*J* in Hz)	δ_C	COSY	^{1}H-^{13}C HMBC	ROESY
1		172.0			
2		104.6			
3		200.0			
4	3.35, m	43.0	5a, 18	3, 5	6, 18
5a	1.31, m	40.8	4	3, 4, 7, 19	5b, 6, 8b
5b	1.20, dd (*15.0, 6.8*)		6	3, 4, 6, 18	5a, 6, 19
6	1.93, m	37.5	5b, 19	7, 8, 19	4, 5a/b, 19
7		116.4			
8a	2.85, m	42.6	8b	6, 7, 9	8b, 11
8b	2.25, d (*17.0*)		8a	6, 9	5a, 8a, 11
9		159.4			
10	3.17, s	50.7	11	8, 9, 11, 14, 15, 16	11, 14a
11	4.22, s	67.9	10, 12	9, 12, 13, 15	8a/b, 10, 12, 13
12	4.82, d (*3.7*)	85.6	11	10, 14, 16	11, 13, 17
13	2.83, m	26.3	14a, 17		11, 12, 14a/b, 17
14a	2.68, dd (*12.7, 11.8*)	33.7	13, 14b	10, 15, 16, 17	10, 13, 14b
14b	1.42, d (*12.7*)		14a	10, 13, 15, 17	13, 14a
15		79.0			
16		185.5			
17	1.15, d (*7.1*)	18.7	13	12, 13, 14	12, 13
18	1.06, d (*6.6*)	17.1	4	3, 4, 5	4
19	1.09, d (*6.5*)	14.5	6	5, 6, 7	5b, 6

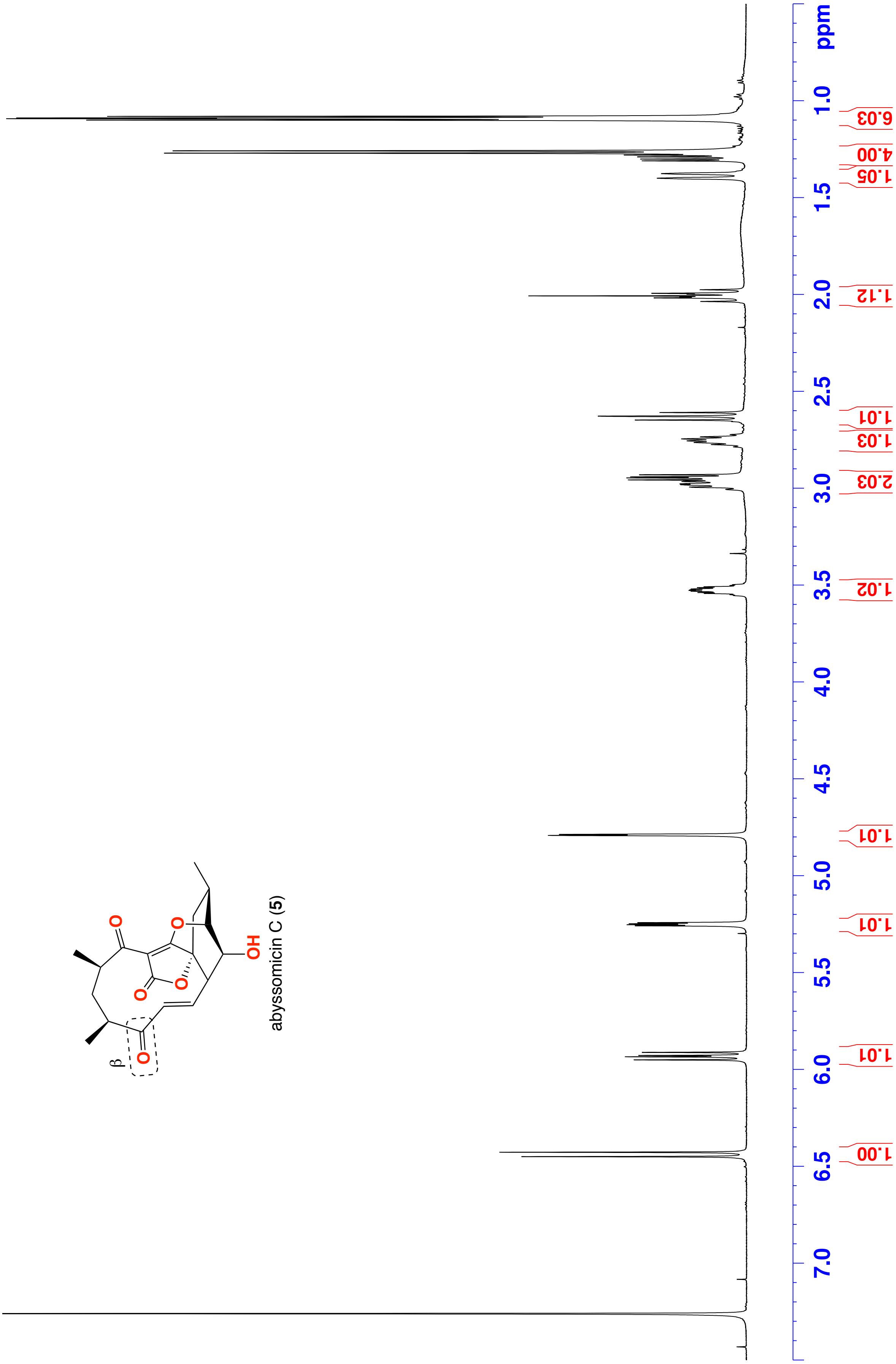

Figure S5a. ^{1}H NMR spectrum (600 MHz, $CDCl_3$) of abyssomicin C (**5**)

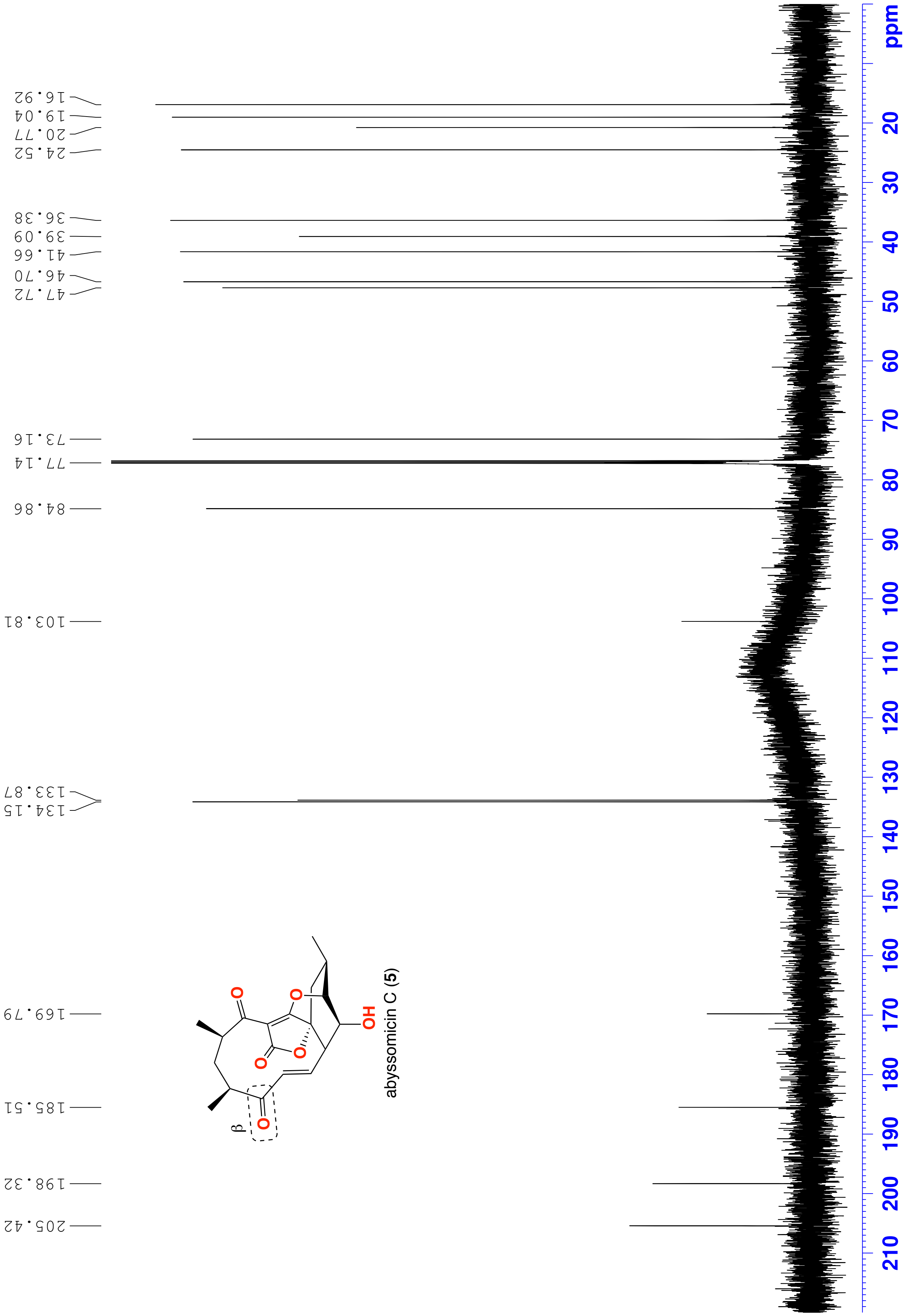

Figure S5b. ^{13}C NMR spectrum (150 MHz, $CDCl_3$) of abyssomicin C (**5**)

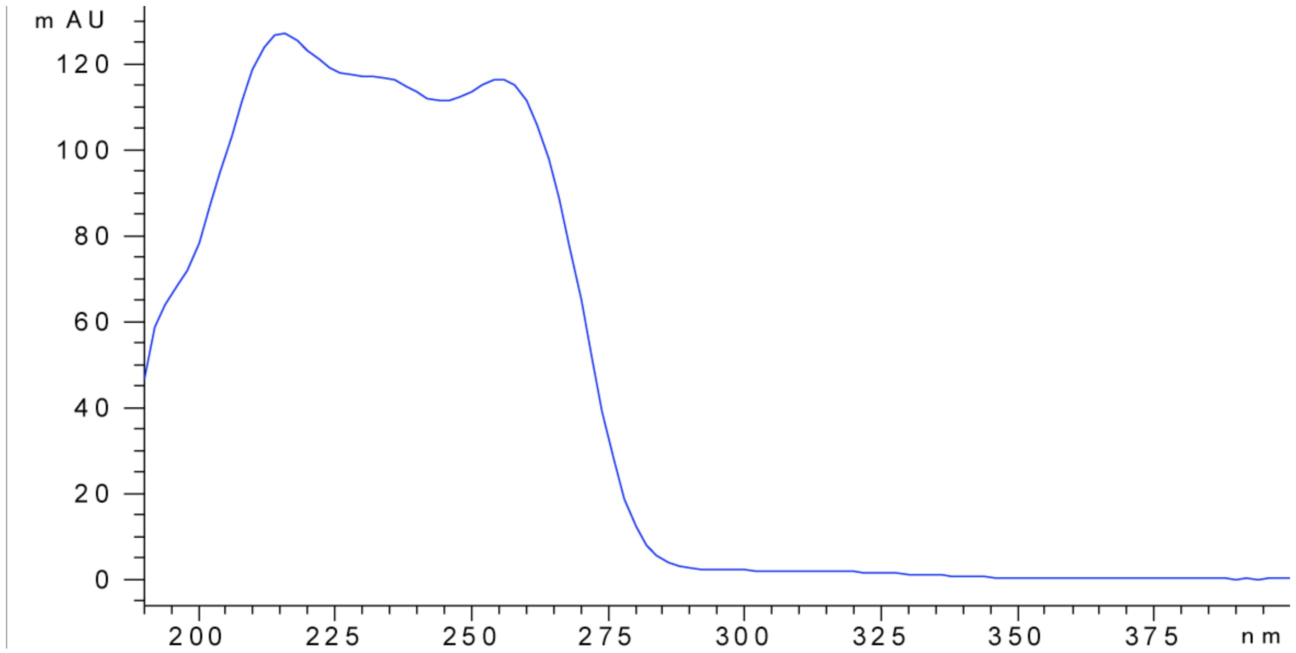

Figure S5c. UV-vis spectrum of abyssomicin C (**5**)

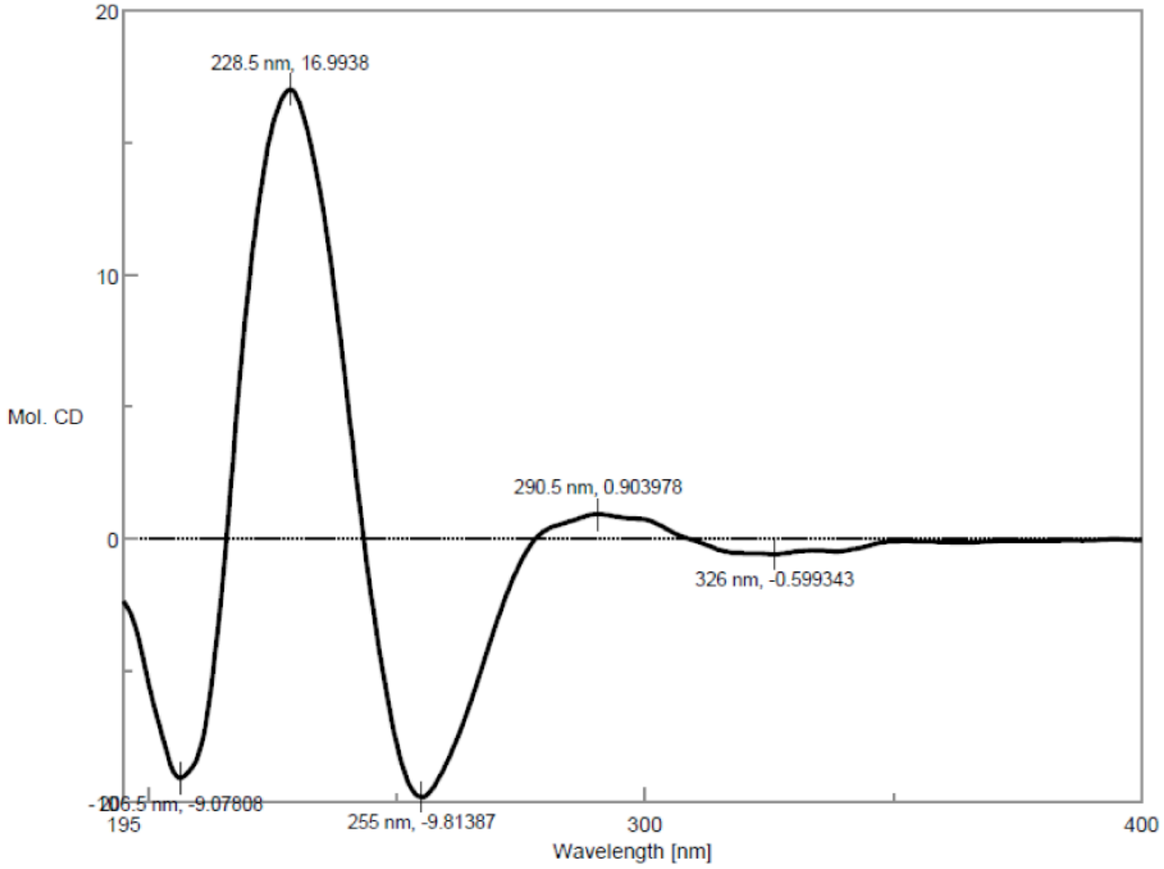

Figure S5d. CD spectrum of abyssomicin C (**5**) in MeOH

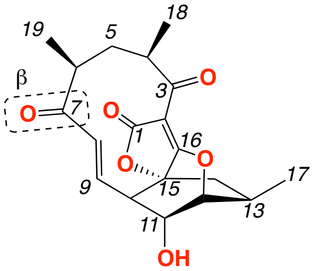

abyssomicin C (**5**)

Table S5. NMR data (600 MHz, $CDCl_3$) for abyssomicin C (**5**)

Pos.	δ_H, mult (*J* in Hz)	δ_C	COSY	^{1}H-^{13}C HMBC
1		169.8		
2		103.8		
3		198.3		
4	3.53, dqd (*13.2, 6.6, 2.7)*	41.7	5a, 18	3, 5, 6,18
5a	2.00, ddd (*14.4, 14.2, 13.2*)	39.1	4, 5b, 6	3, 4, 6, 7
5b	1.39, ddd (*14.4, 2.7, 1.2*)		5a	3, 4, 6, 7, 18, 19
6	2.98, dqd (*14.2, 7.2, 1.2*)	46.7	5a, 19	4, 5, 7, 19
7		205.4		
8	6.43, d (*13.6*)	134.1	9	7, 9, 10, 11, 15
9	5.93, dd (*13.6, 9.6*)	133.9	8, 10	7, 8, 10, 11, 15
10	2.94, dd (*9.6, 6.3*)	47.7	9, 11	9, 11, 14, 15, 16
11	5.25, dd (*6.3, 3.2*)	73.1	10, 12	9, 12, 10, 13
12	4.79, d (*3.2*)	84.9	11	10, 11, 14, 16, 17
13	2.75, m	24.5	14a, 14b, 17	14, 17
14a	2.63, dd (*12.4, 11.4*)	36.4	13, 14b	10, 13, 15, 16, 17
14b	1.29, dd (*12.4, 5.6*)		13, 14a	9, 15, 16, 17
15		77.1		
16		185.5		
17	1.26, d (*7.2*)	19.0	13	12, 13, 14
18	1.09, d (*6.6*)	16.9	4	3, 4
19	1.09, d (*7.2*)	20.8	6	5, 6, 7

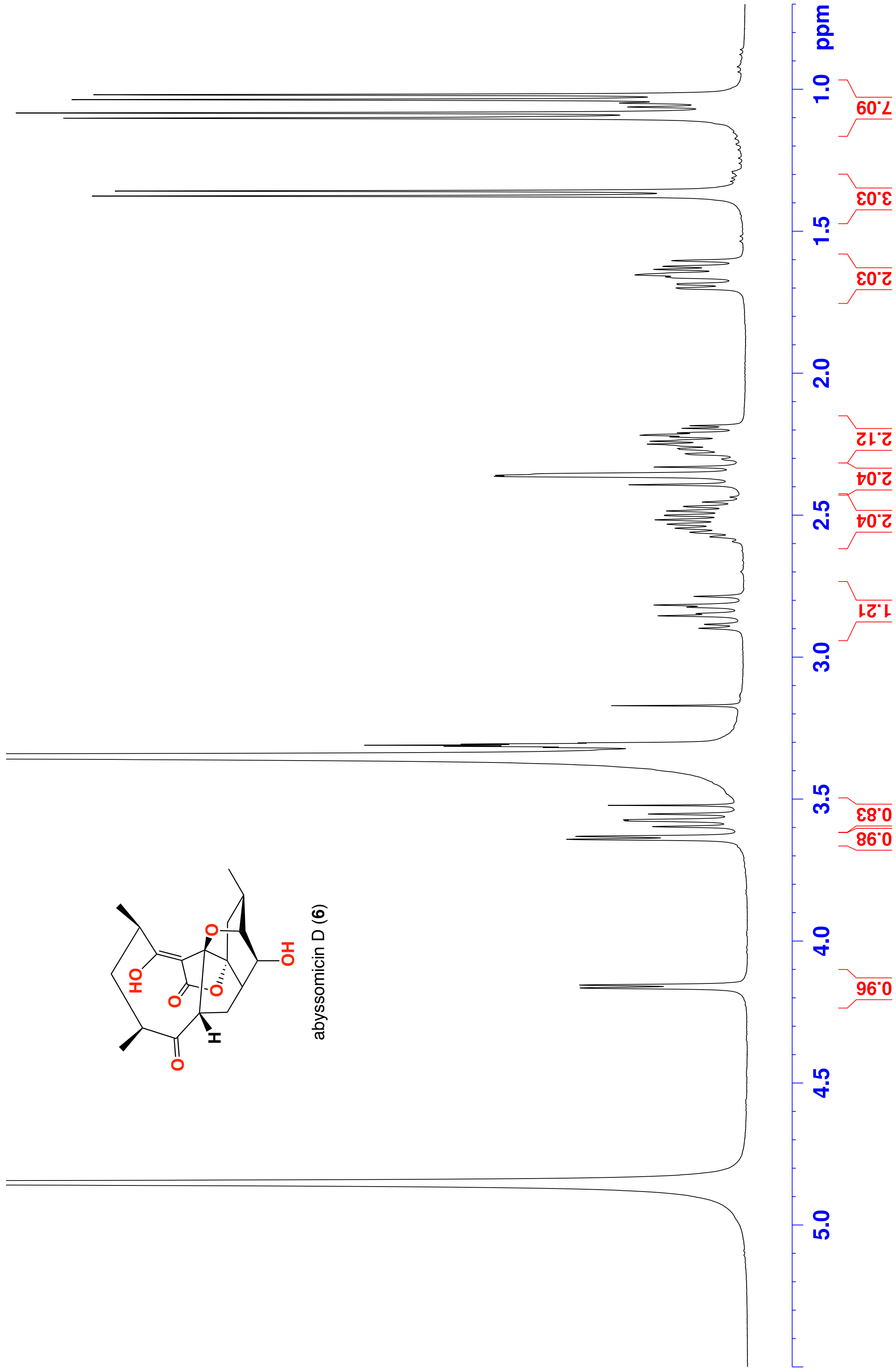

Figure S6a. ^{1}H NMR spectrum (400 MHz, methanol-d_4) of abyssomicin D (**6**)

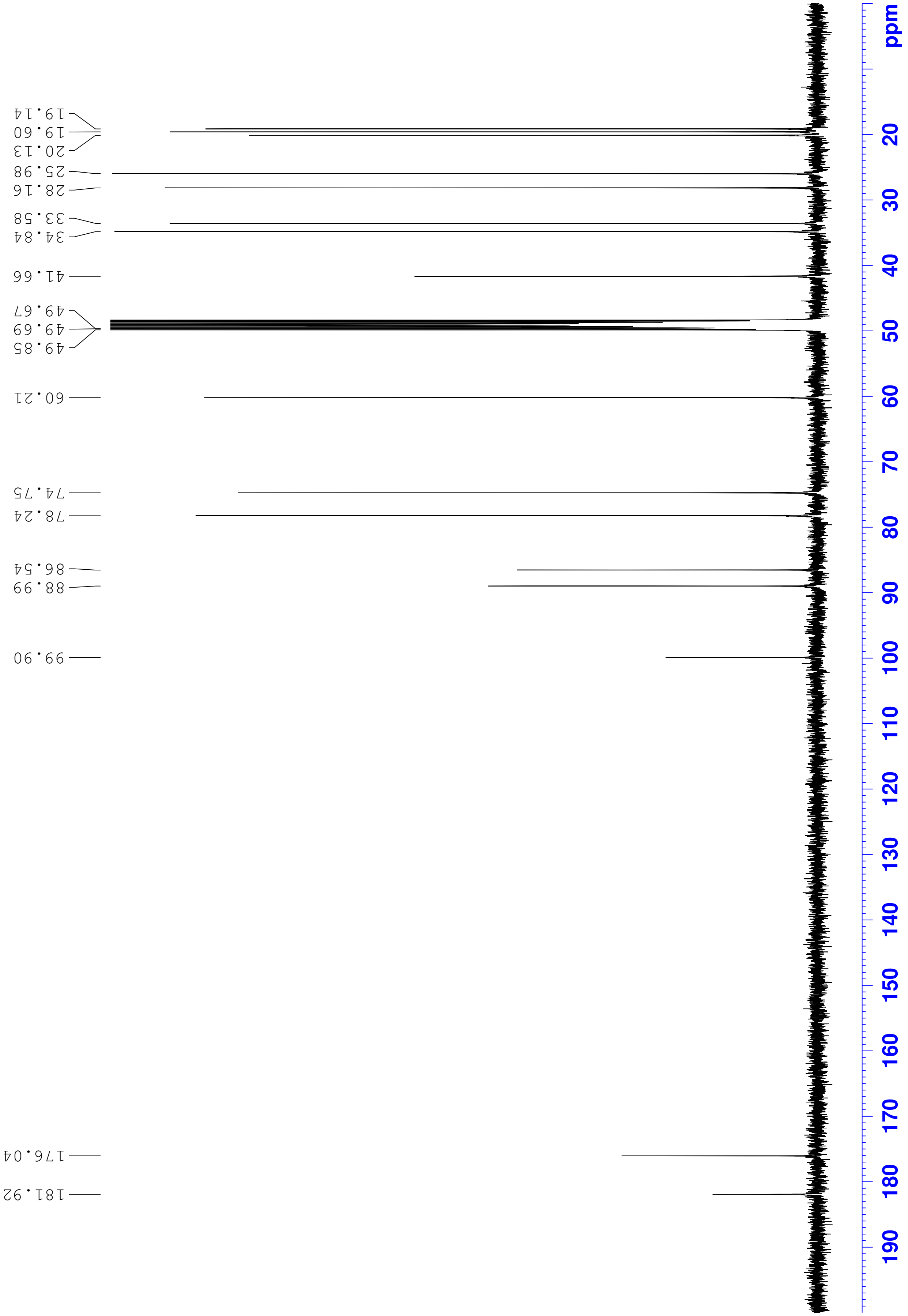

Figure S6b. ^{13}C NMR spectrum (100 MHz, methanol-d_4) of abyssomicin D (**6**)

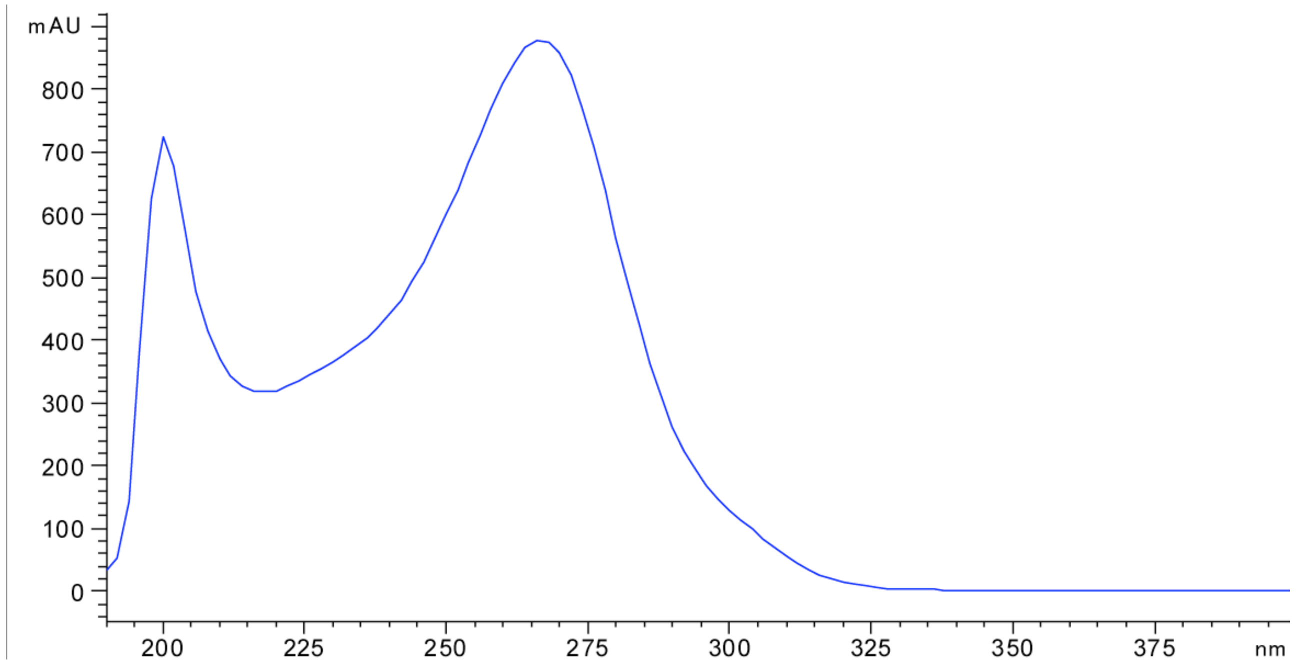

Figure S6c. UV-vis spectrum of abyssomicin D (**6**)

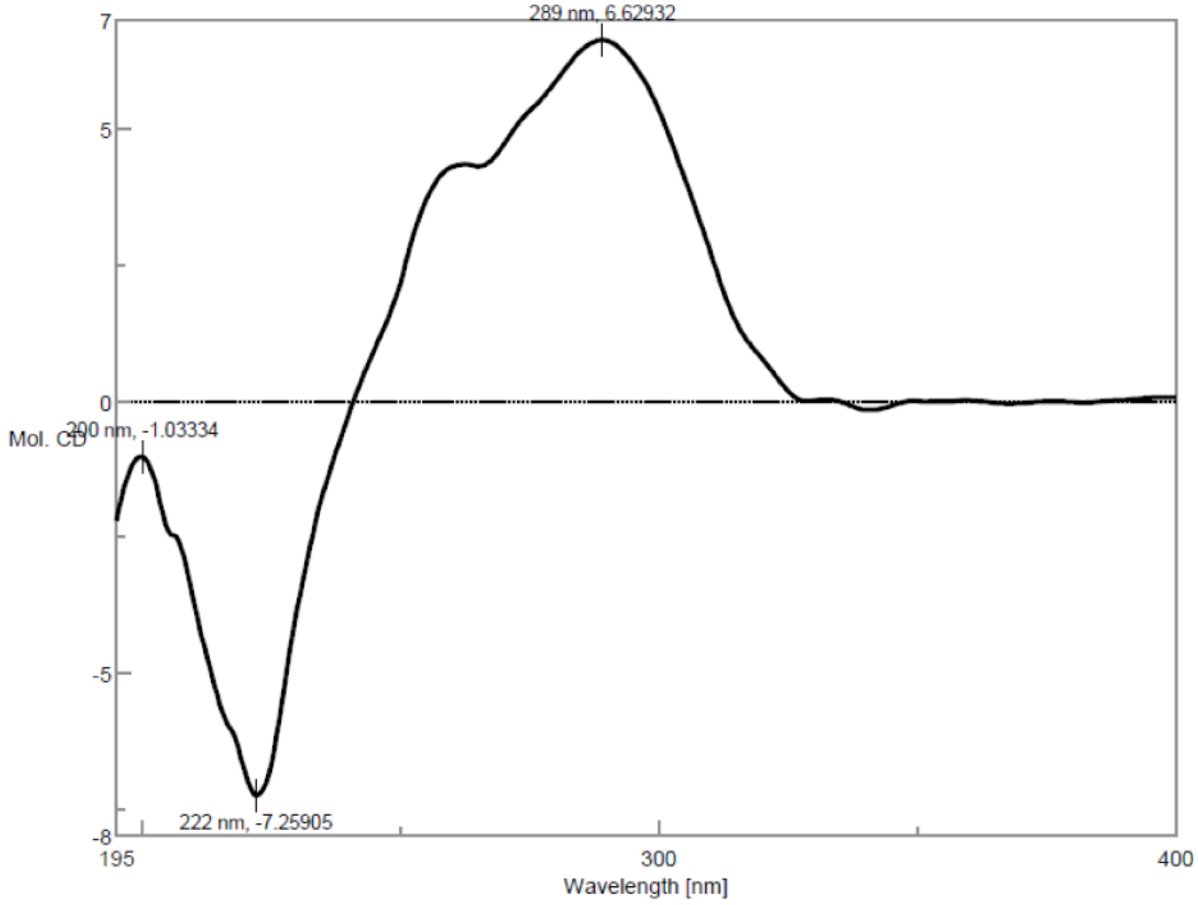

Figure S6d. CD spectrum of abyssomicin D (**6**) in MeOH

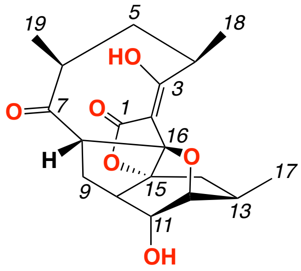

abyssomicin D (**6**)

Table S6. NMR data (400 MHz, methanol-d_4) for abyssomicin D (**6**)
]

Pos.	δ_H, mult (*J* in Hz)	δ_C	COSY	^{1}H-^{13}C HMBC	ROESY
1		176.0			
2		99.9			
3		182.0			
4	2.49, m	41.7	5a/b, 18	2, 3, 5, 6, 18	18
5a	2.87, m	34.8	4, 5b, 6	4, 6, 7, 18	5b
5b	1.70, m		4, 5a	3, 4, 6, 7, 19	5a, 18, 19
6	2.29, m	48.0	5a, 19	4, 5, 7, 8, 19	18
7		213.1			
8	2.36, s	49.7		15, 16	
9a	2.23, m	28.2	9b, 10	7, 8, 10, 11	9b, 10
9b	1.64, m		9a, 10	8, 10, 11, 15, 16	9a, 10, 11
10	3.60, dd (*9.6, 8.2*)	60.2	9a/b	7, 8, 9, 16	9a/b, 11
11	4.18, d (*4.1*)	74.7	12	9, 13, 15	9b, 12
12	3.66, d (*4.0*)	78.2	11	11, 14, 17	11, 13, 17
13	2.58, m	26.0	14a, 17	14, 15, 17	14a/b, 17
14a	2.38, m	33.6	13, 14b	10, 12, 13, 15, 16, 17	13, 14b
14b	1.08, d (*5.6*)		14a	13, 15, 16, 17	13, 14a
15		89.0			
16		86.5			
17	1.05, d (*6.9*)	19.1	13	12, 13, 14	12, 13
18	1.39, d (*7.0*)	20.1	4	3, 4, 5	4, 5b, 6
19	1.11, d (*7.4*)	19.6	6	5, 6, 7	

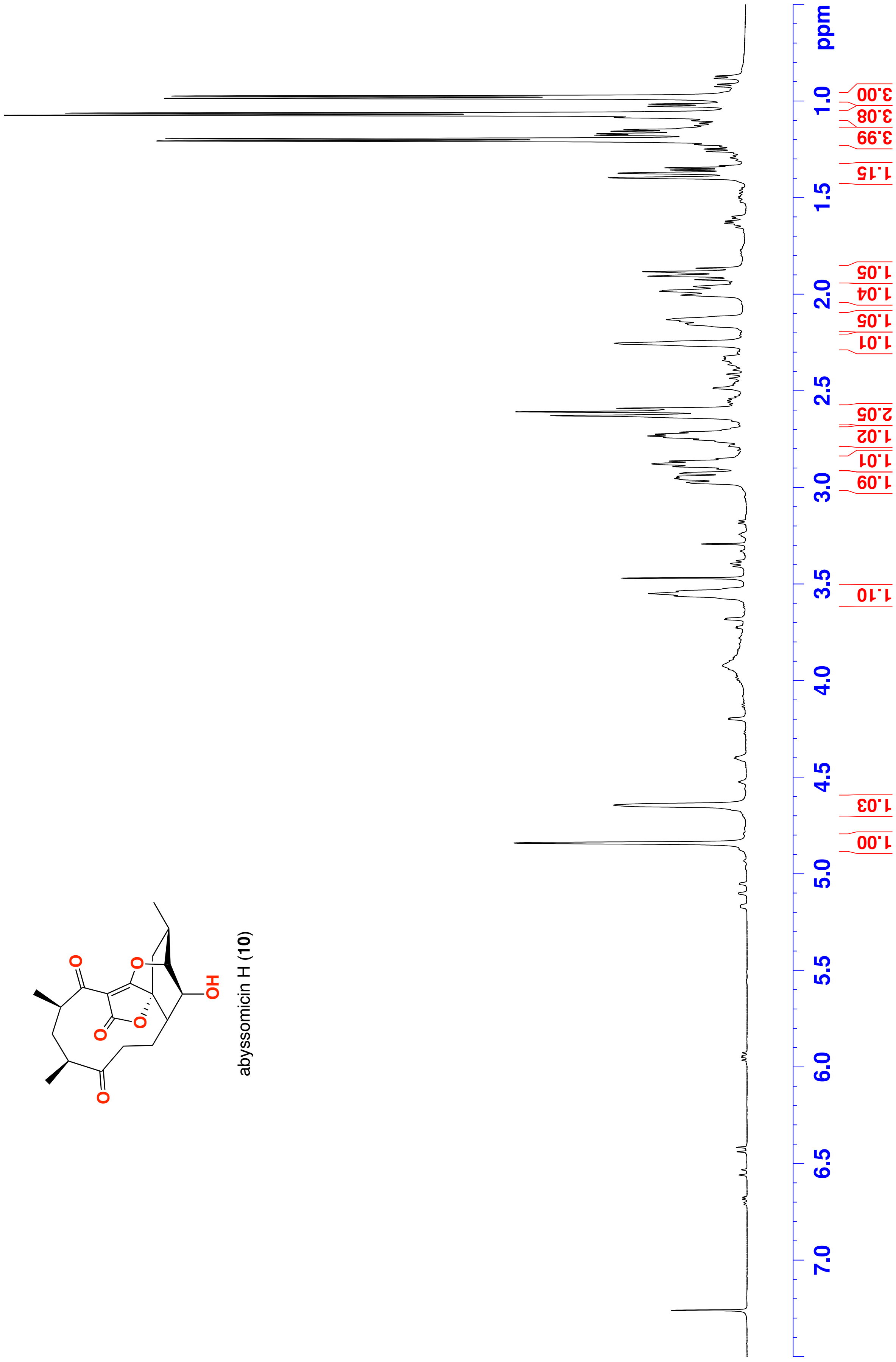

Figure S7a. ^{1}H NMR spectrum (600 MHz, $CDCl_3$) of abyssomicin H (**10**)

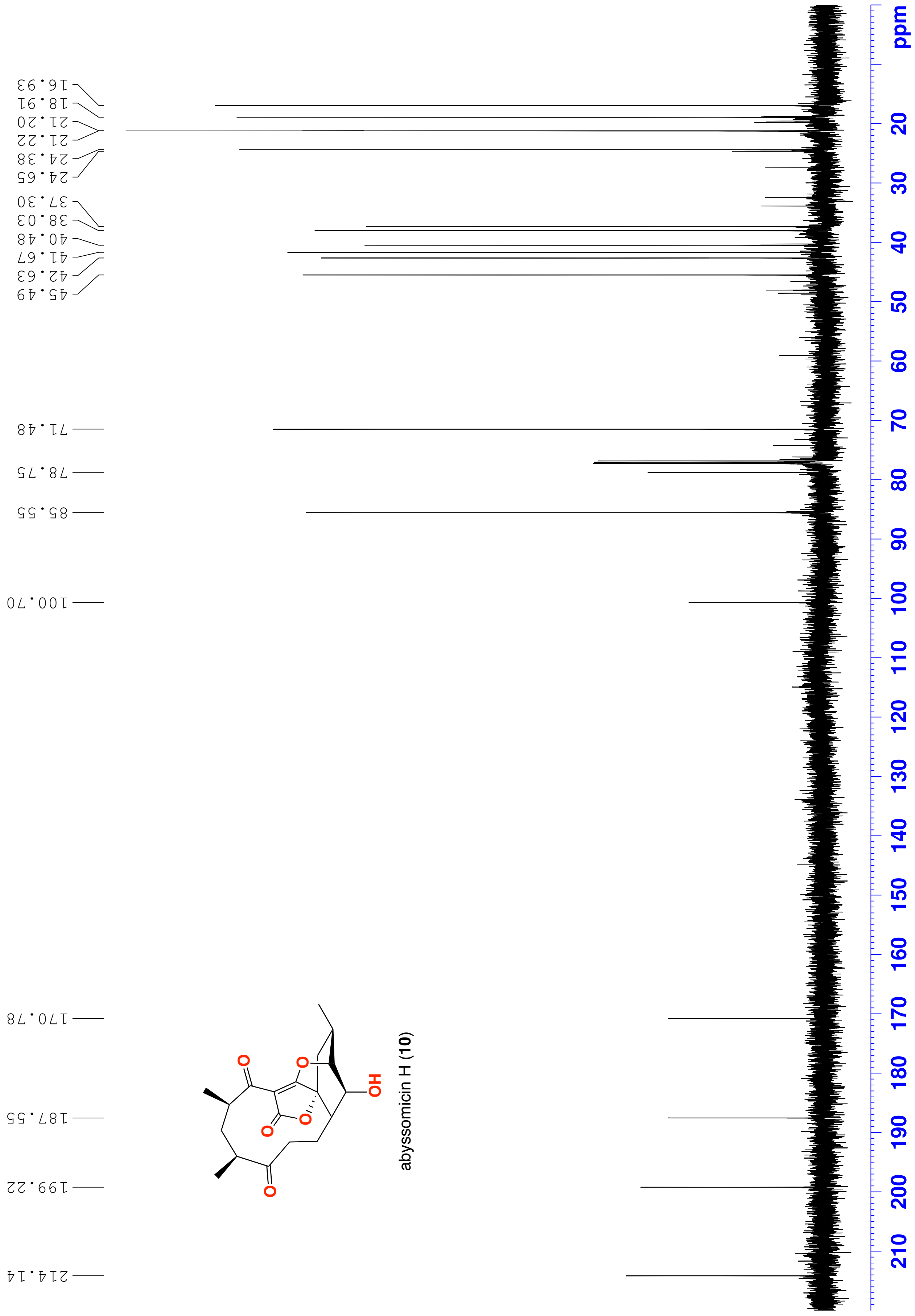

Figure S7b. ^{13}C NMR spectrum (150 MHz, $CDCl_3$) of abyssomicin H (**10**)

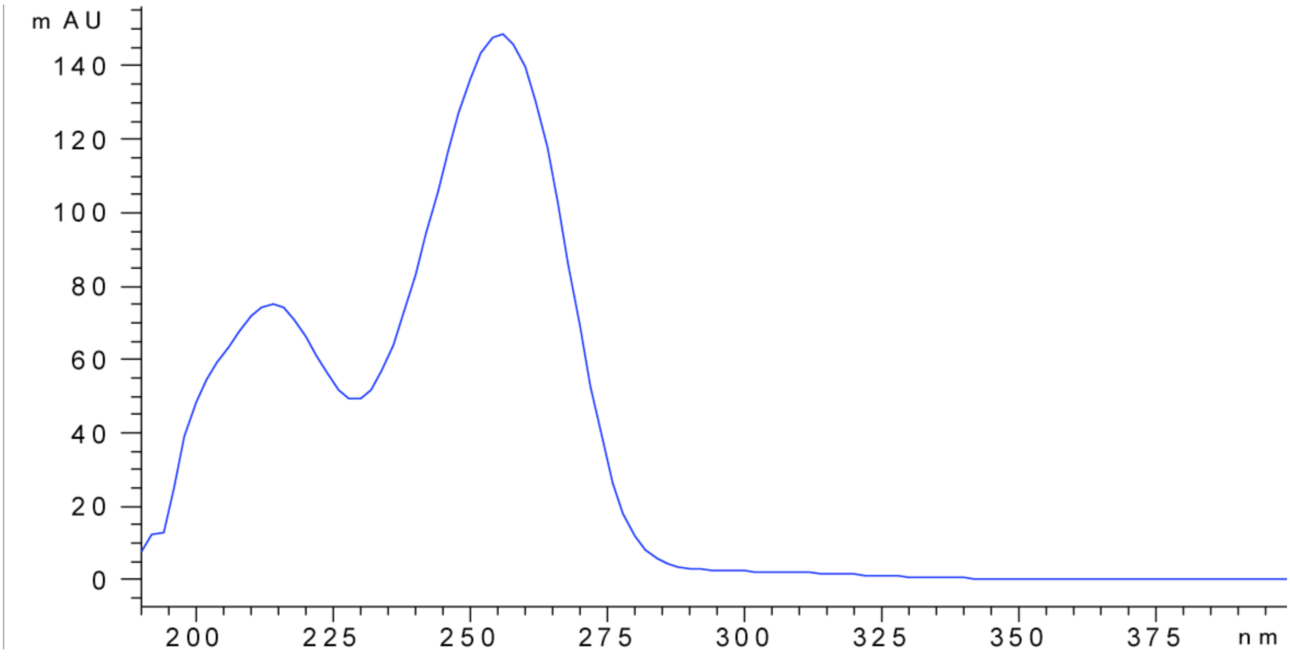

Figure S7c. UV-vis spectrum of abyssomicin H (**10**)

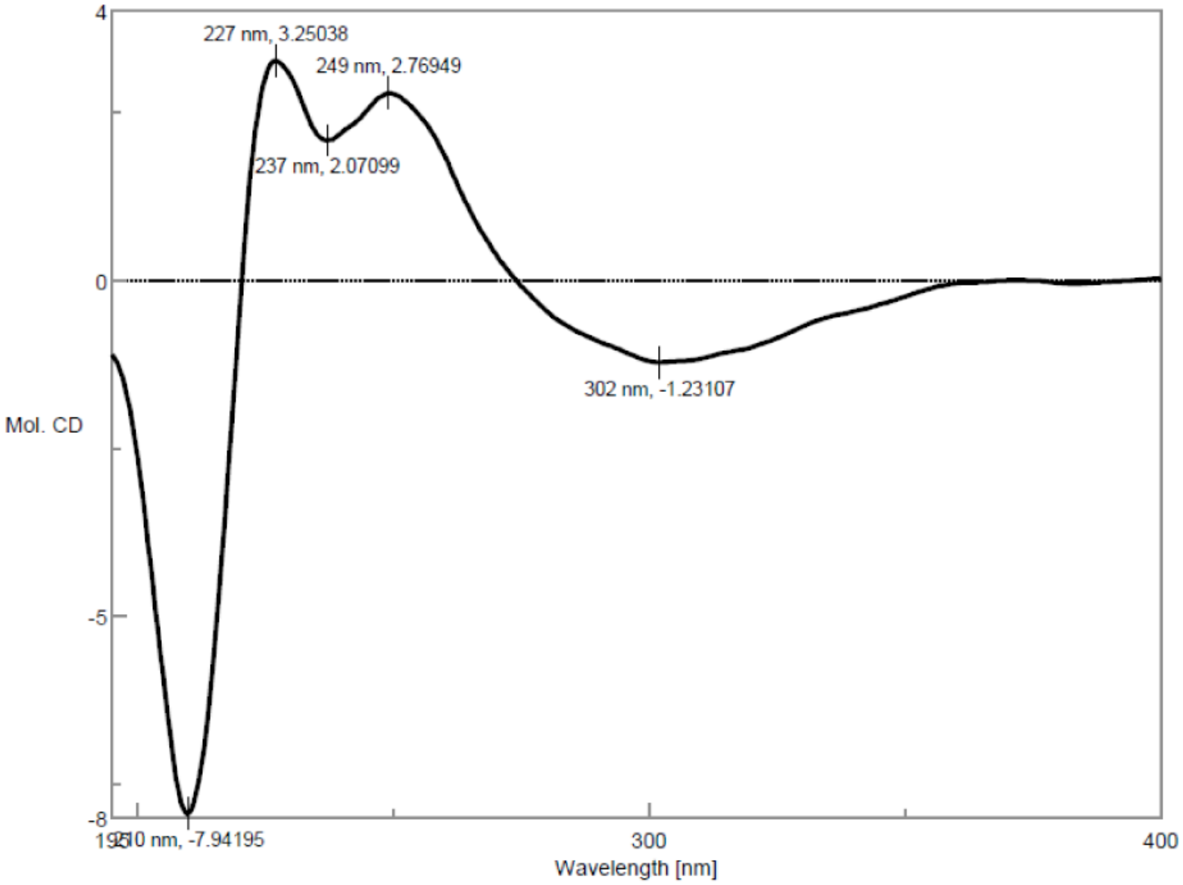

Figure S7d. CD spectrum of abyssomicin H (**7**) in MeOH

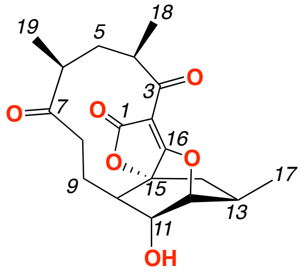

abyssomicin H (**10**)

Table S7. NMR data (600 MHz, $CDCl_3$) for abyssomicin H (**10**)

Pos.	δ_H, mult (*J* in Hz)	δ_C	COSY	^{1}H-^{13}C HMBC
1		170.8		
2		100.7		
3		199.2		
4	3.55, m	41.7	5a, 18	3, 5
5a	1.90, ddd (*14.1, 10.9, 10.9*)	40.5	4, 5b, 6	3, 4, 7
5b	1.39, d (*14.1*)		5a	3, 6, 7, 18
6	2.88, m	45.5	5a, 19	5, 7, 19
7		214.1		
8a	2.95, m	37.3	8b, 9b	7, 9
8b	2.63, m		8a, 9a	7, 10, 15
9a	2.15, dd (*11.1, 4.4*)	21.1	8b, 9b, 10	7, 8, 11
9b	1.98, m		8a, 9a	15
10	2.25, s	42.6	9a, 11	8, 9, 11, 15, 16
11	4.65, s	71.5	10, 12	9, 12
12	4.84, s	85.5	11	10, 11, 16
13	2.74, m	24.4	14a, 17	14, 17
14a	2.61, m	38.0	13, 14b	10, 13, 15, 16, 17
14b	1.16, dd (*12.3, 4.9*)		14a	13, 15, 16, 17
15		78.8		
16		187.6		
17	1.20, d (*7.0*)	18.9	13	12, 13, 14
18	1.07, d (*6.5*)	16.9	4	3, 4, 5
19	0.98, d (*7.0*)	21.2	6	5, 6, 7

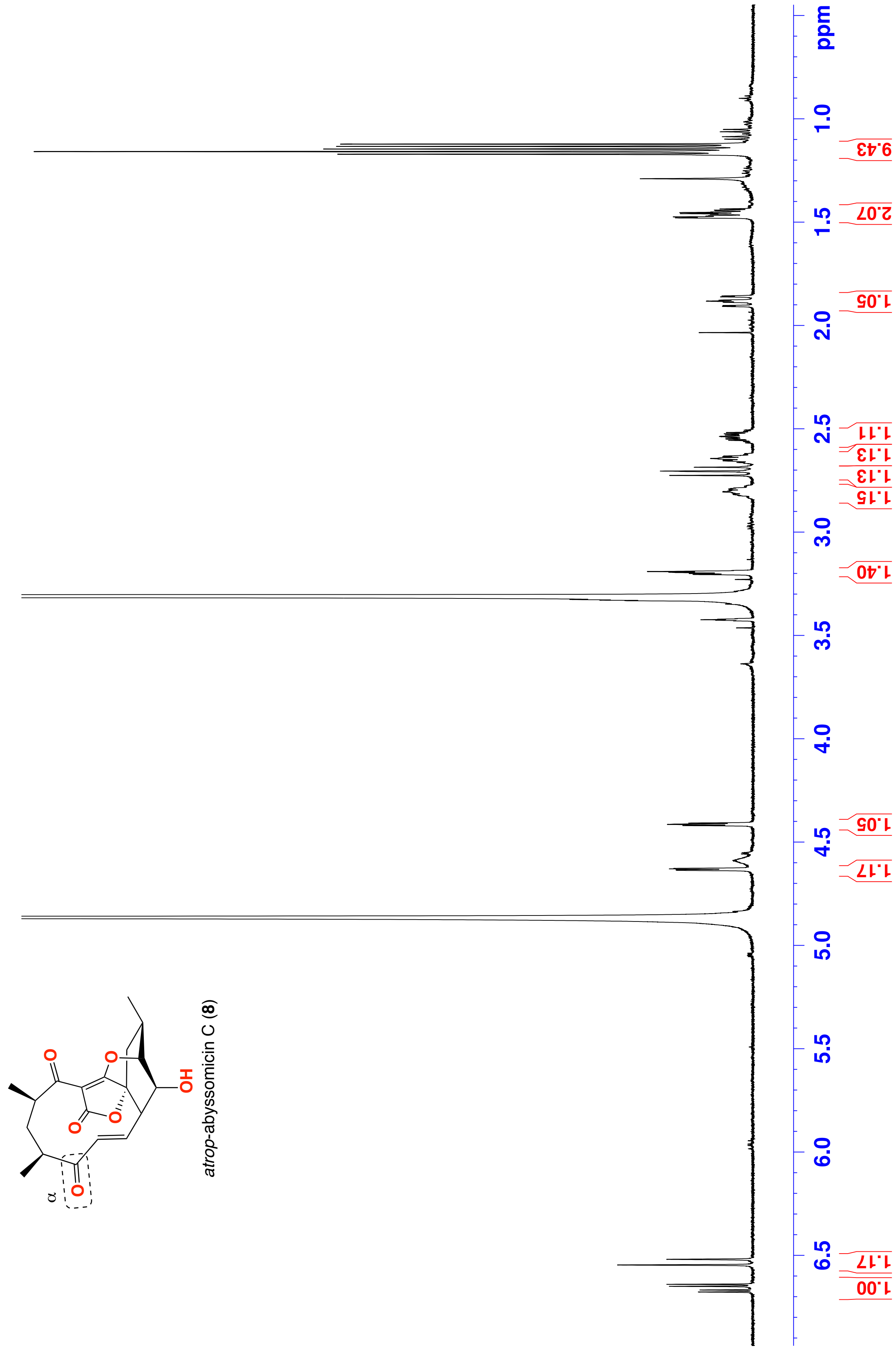

Figure S8a. ^{1}H NMR spectrum (600 MHz, methanol-d_4) of *atrop*-abyssomicin C (**8**)

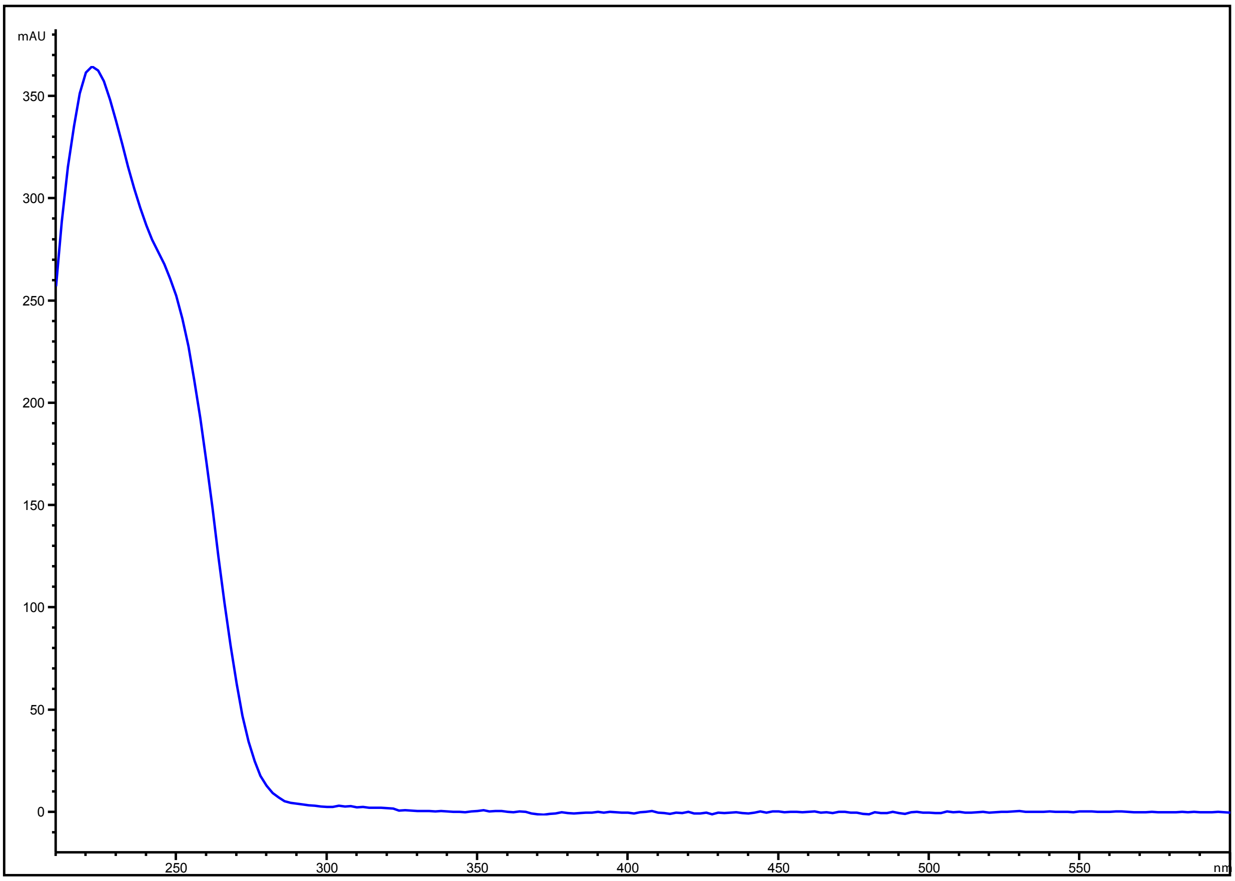

Figure S8b. UV-vis spectrum of *atrop*-abyssomicin C (**8**)

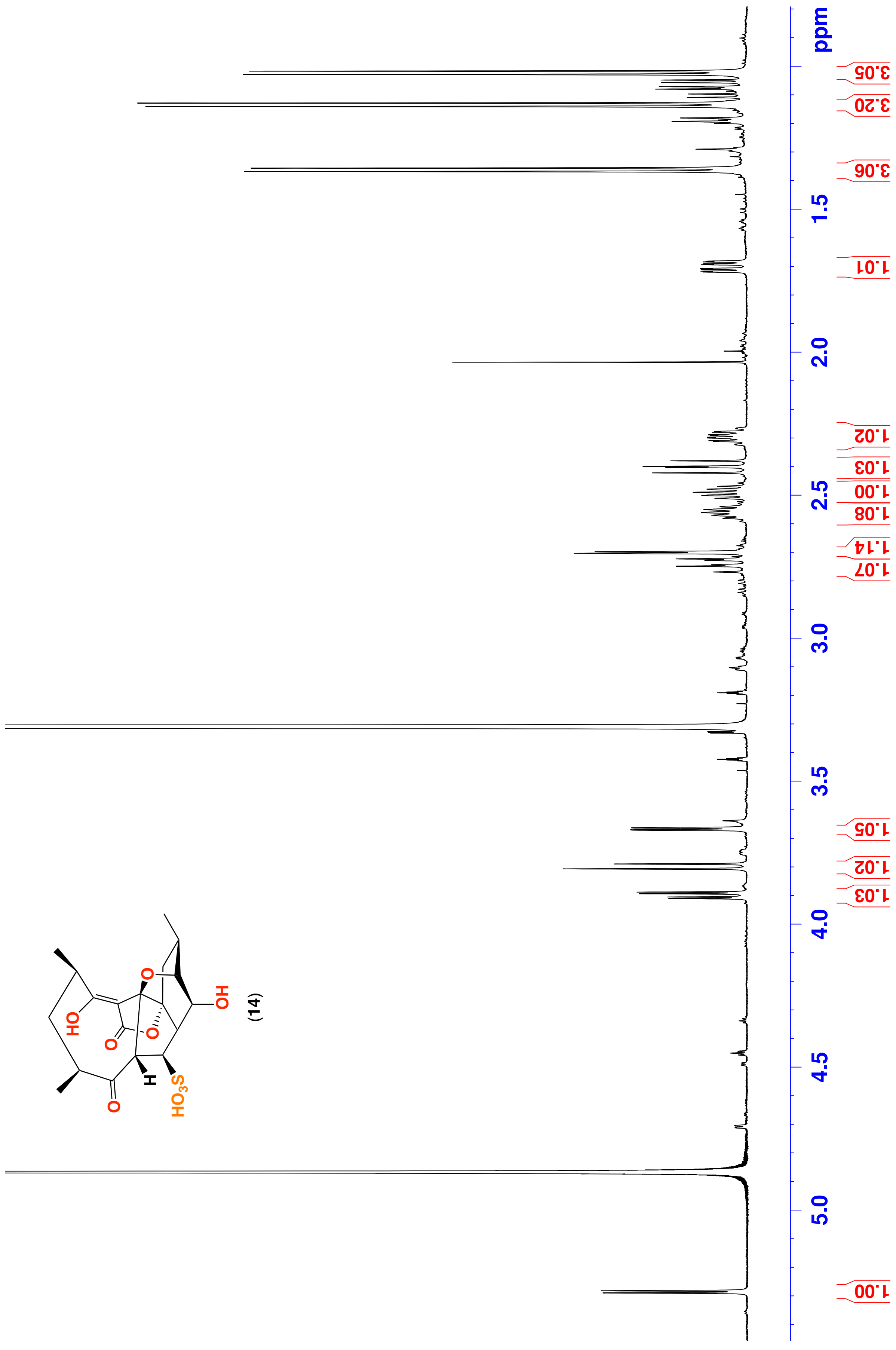

Figure S9a. ^{1}H NMR spectrum (600 MHz, methanol-d_4) of sulfonic acid (**14**)

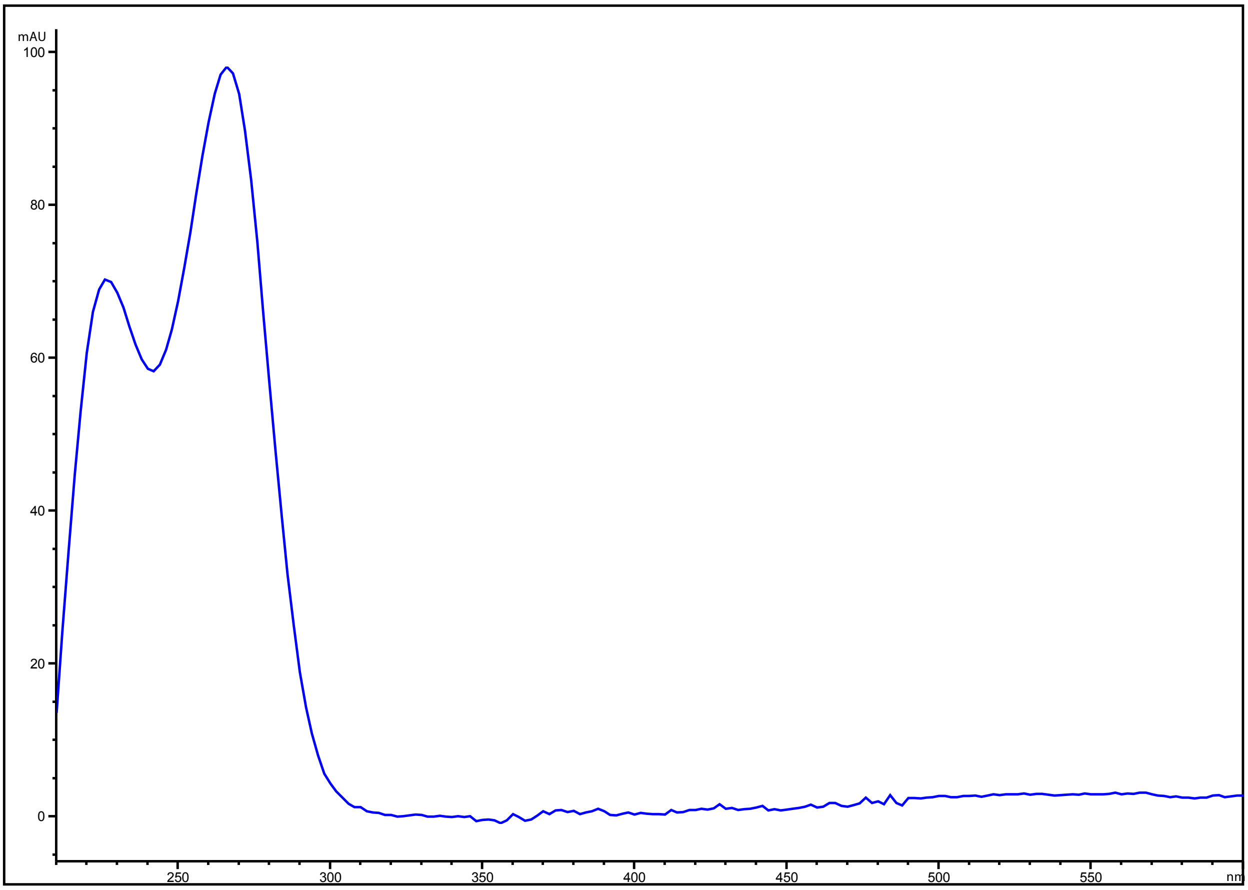

Figure S9b. UV-vis spectrum of sulfonic acid (**14**)

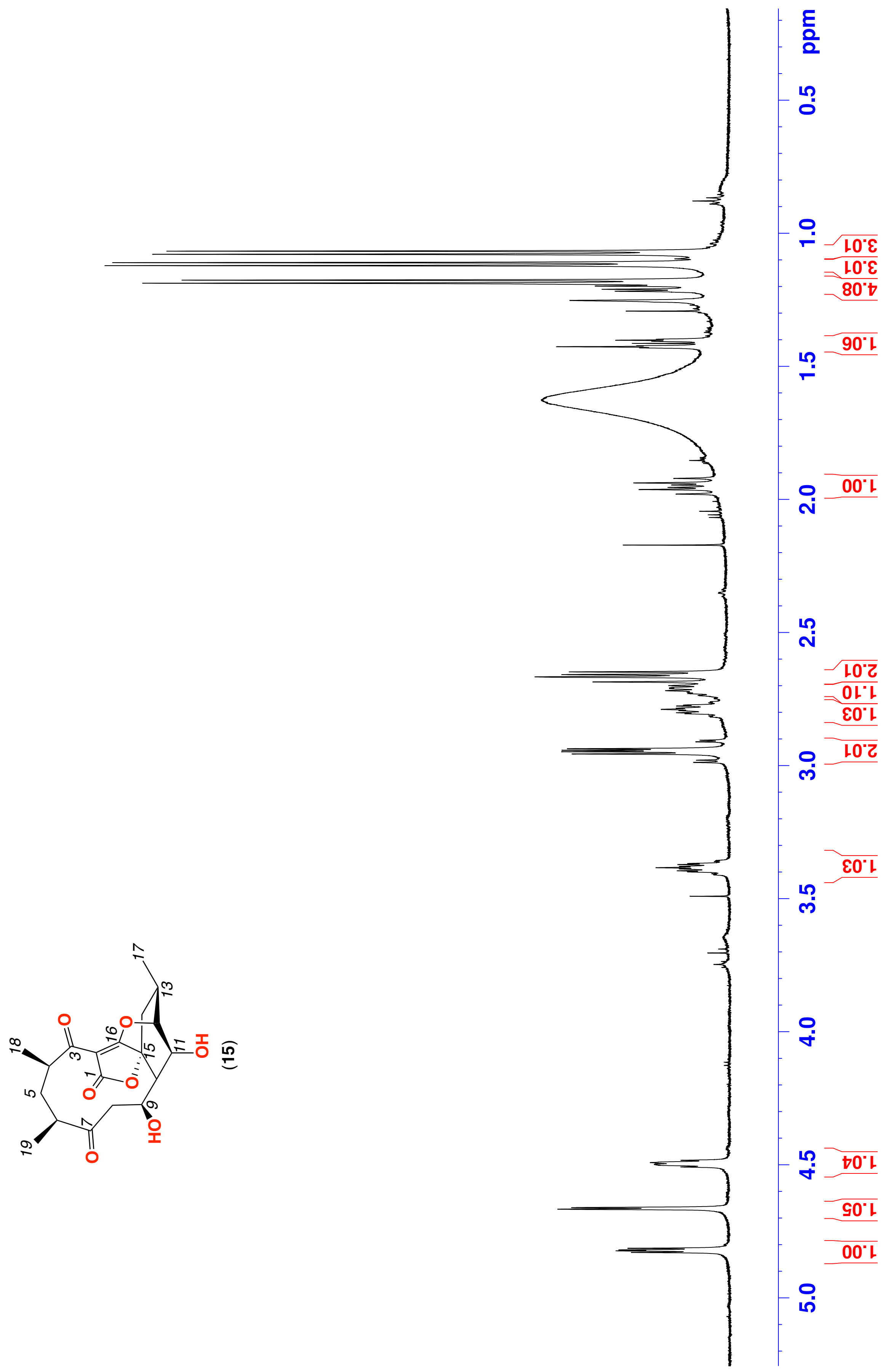

Figure S10a. ^{1}H NMR spectrum (600 MHz, $CDCl_3$) of abyssomicin **15**

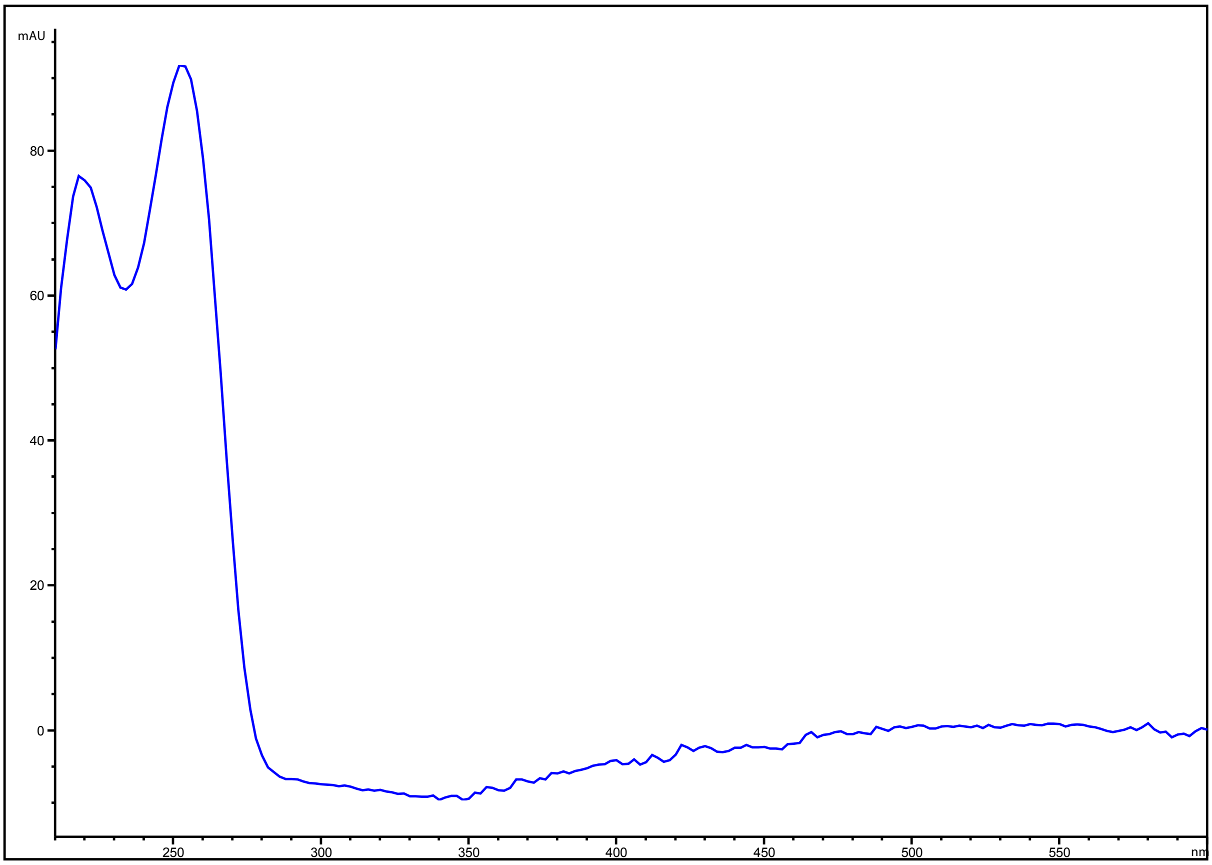

Figure S10b. UV-vis spectrum of abyssomicin **15**

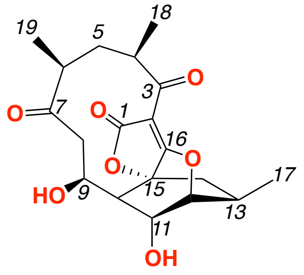

(**15**)

Table S10. NMR data (600 MHz, $CDCl_3$) for abyssomicin **15**

Pos.	δ_H, mult (*J* in Hz)	δ_C	COSY	^1H-^{13}C HMBC
1		[a]		
2		[a]		
3		197.7		
4	3.38, m	42.4	5a, 5b, 18	3, 5
5a	1.95, ddd (*14.9, 10.3, 10.3*)	39.5	4, 5b, 6	3, 6, 7
5b	1.40, ddd (*14.9, 2.4, 2.3*)		4, 5a, 6	3, 6, 7, 18, 19
6	2.79, m	46.6	5a, 5b, 19	4, 19
7		211.1		
8a	2.97, dd (*19.0, 5.0*)	44.4	8b, 9	7, 9, 10
8b	2.93, dd (*19.0, 3.6*)		8a, 9	7, 9, 10
9	4.50, m	64.8	8, 10	7, 10, 11
10	2.66, d (*5.5*)	51.1	9, 11	8, 9, 11, 14, 15, 16
11	4.82, dd (*5.5, 3.6*)	67.1	10, 12	9, 12, 13
12	4.66, d (*3.6*)	83.3	11	10, 11, 14, 16, 17
13	2.70, m	24.7	14a, 14b, 17	14, 17
14a	2.67, m	37.8	13, 14b	10, 13, 15, 16
14b	1.21, dd (*11.6, 4.4*)		13, 14a	10, 12, 13, 15, 17
15		76.8		
16		184.2		
17	1.19, d (*7.0*)	18.8	13	12, 13, 14
18	1.12, d (*6.8*)	16.8	4	3, 4, 5
19	1.08, d (*7.2*)	20.4	6	5, 6, 7

[a] Not observed

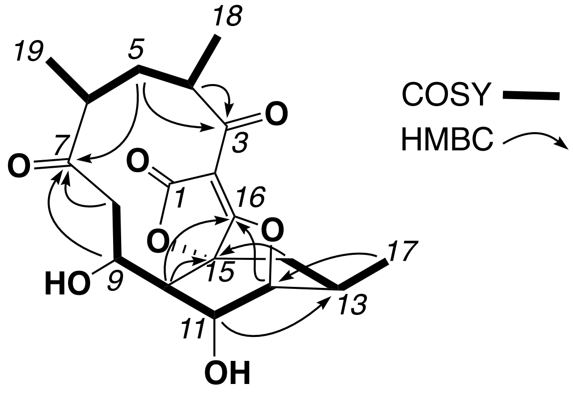

(**15**)

Figure S10c. Selected 2D NMR correlations for abyssomicin **15**

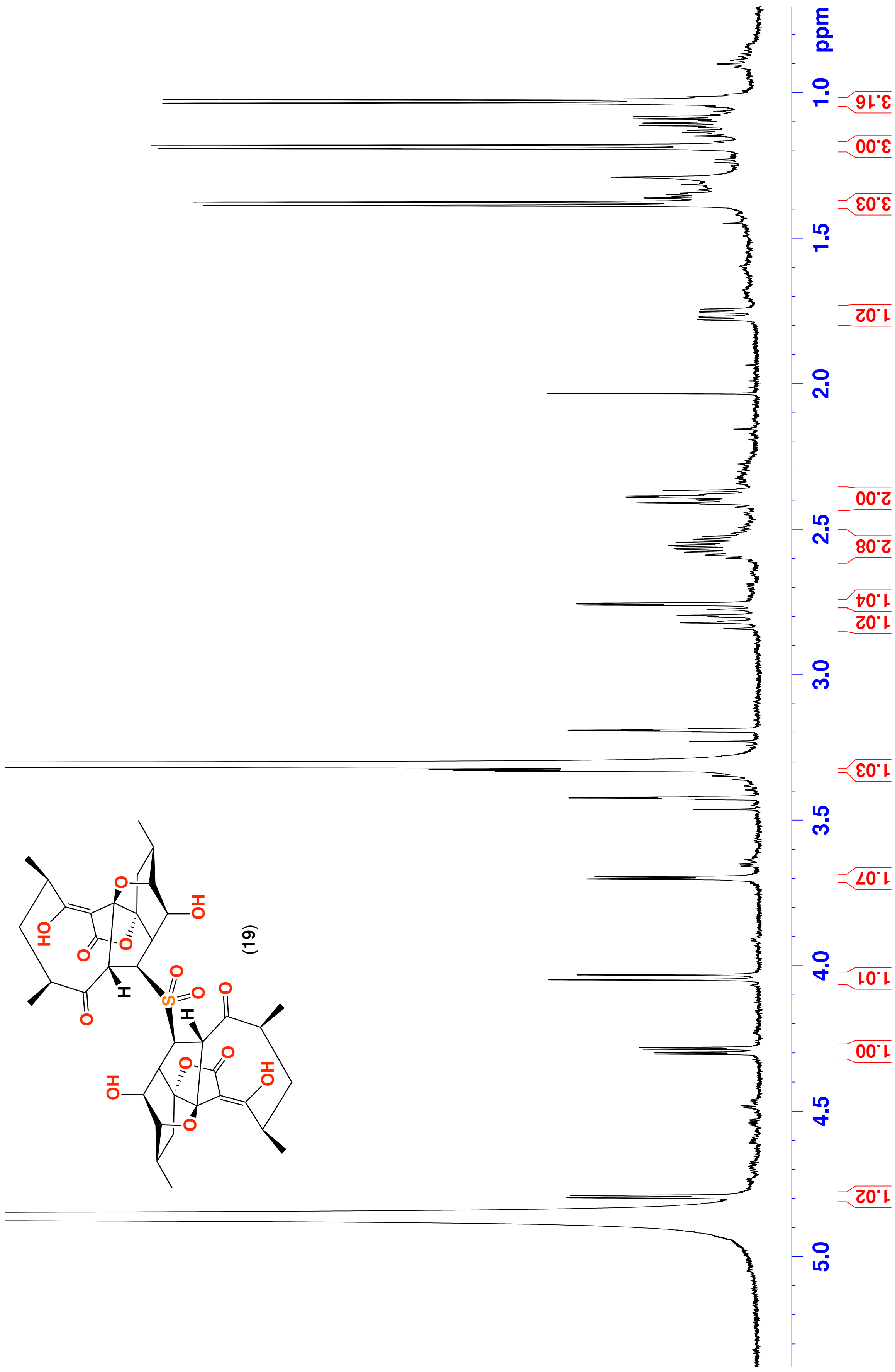

Figure S11a. ^{1}H NMR spectrum (600 MHz, methanol-d_4) of sulfone (**19**)

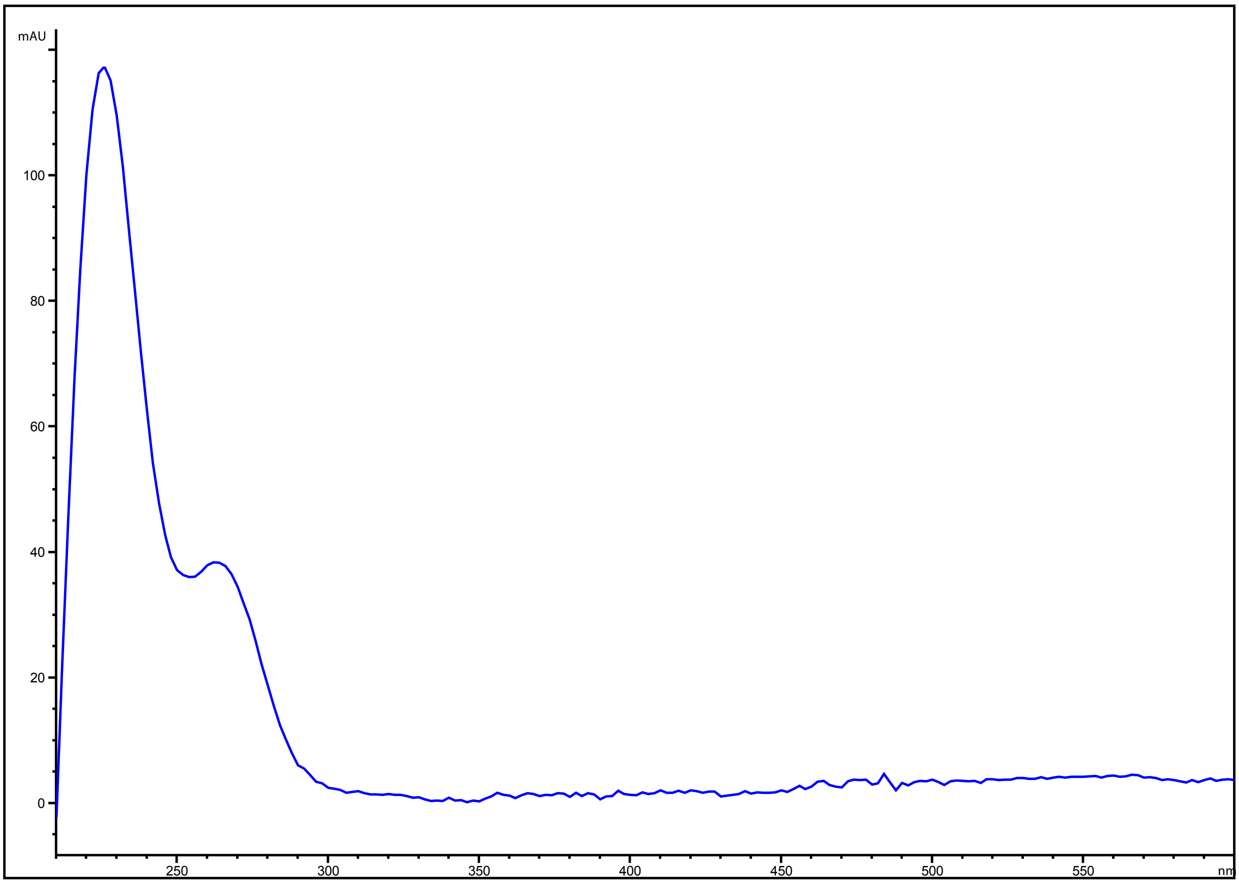

Figure S11b. UV-vis spectrum of sulfone (**19**)

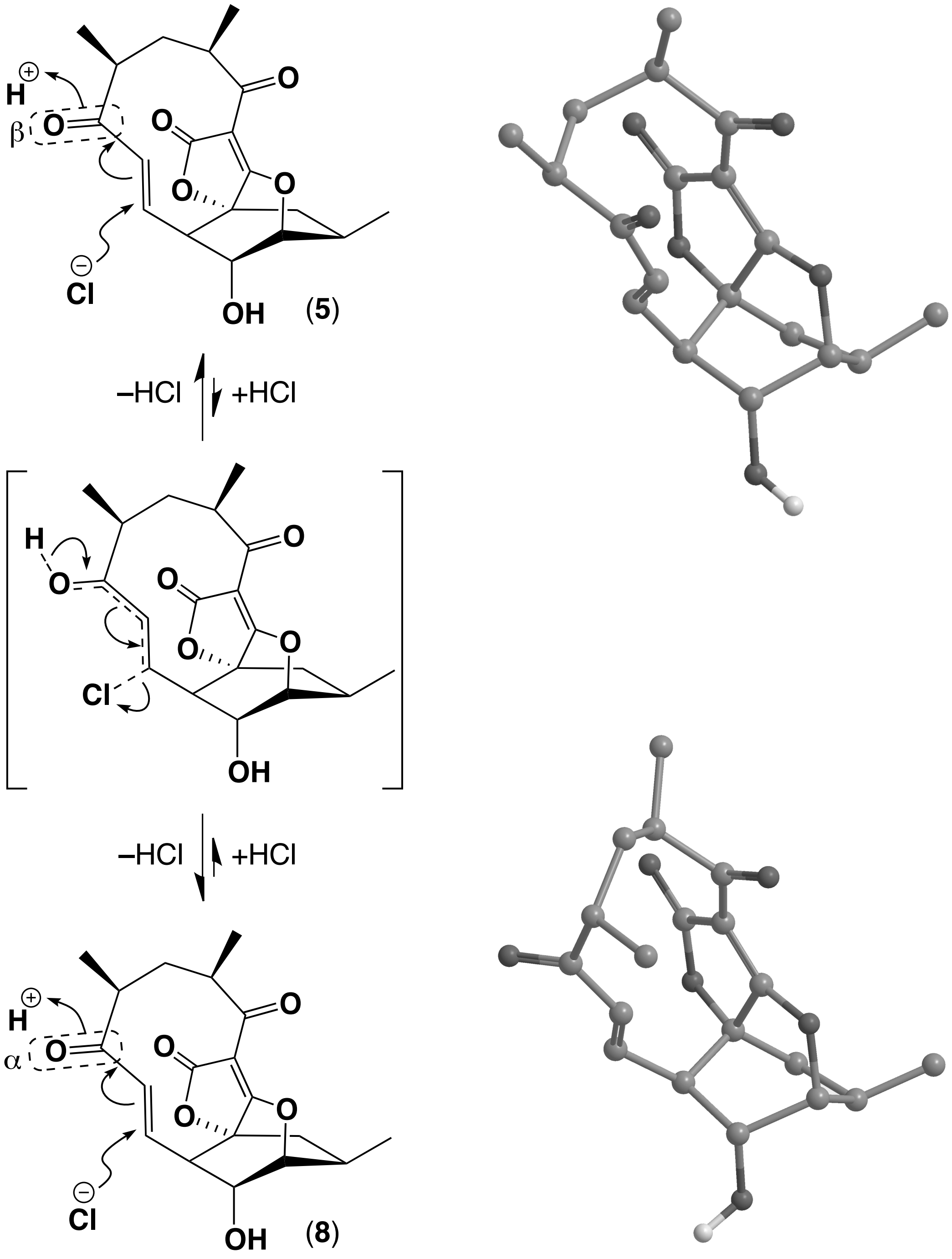

Figure S12a. Acid-catalyzed Michael addition equilibration of atropisomers **5** and **8**, inclusive of energy minimized (MM2) models

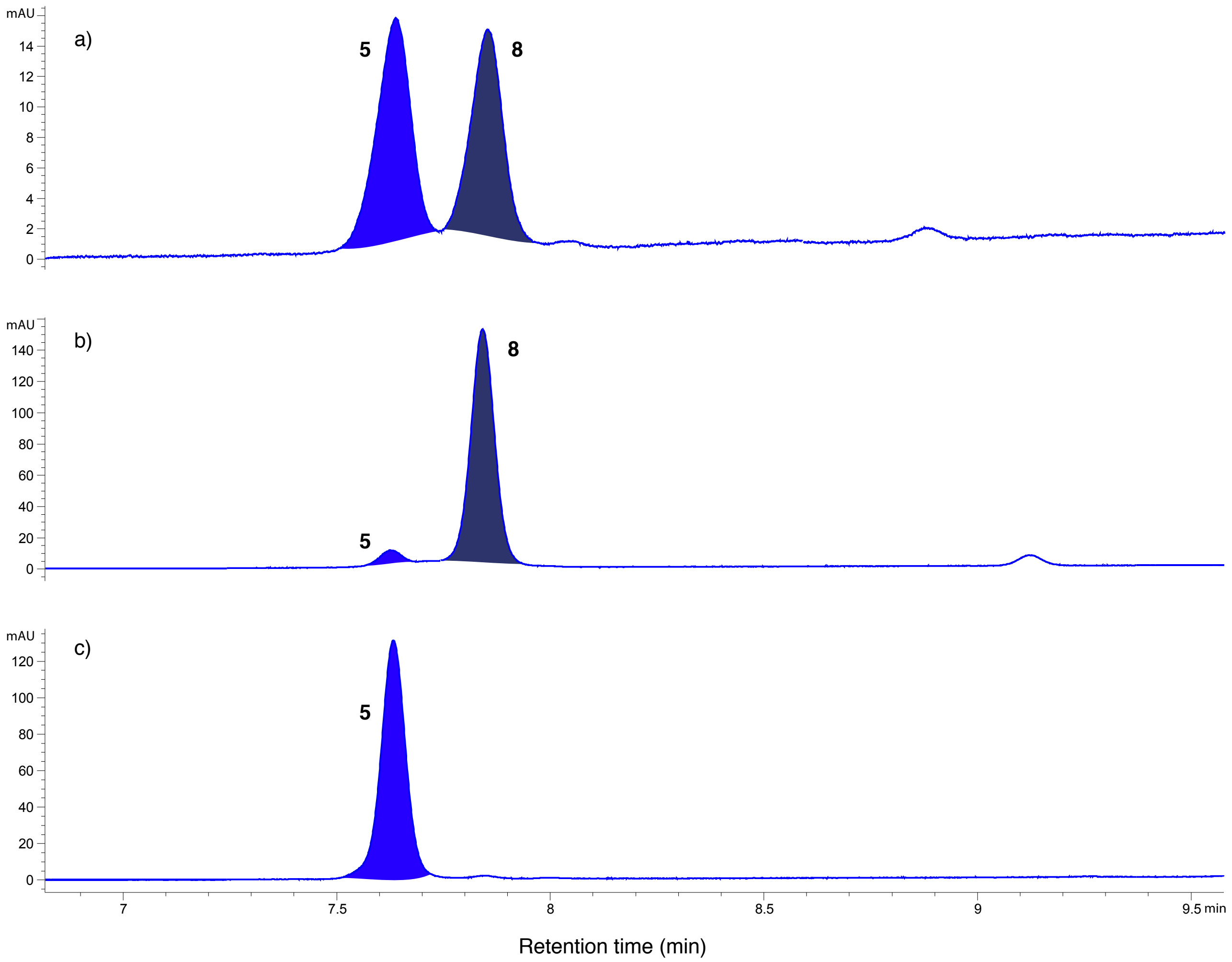

Figure S12b. HPLC (Agilent Zorbax SB-CN, 5 µm, 4.6 × 150 mm, 1.0 mL/min gradient elution from 10–100% MeCN/H_2O over 15 min with isocratic 0.05% formic acid modifier) analysis of (a) abyssomicin C (**5**) treated with 0.2 eq. HCl/Et_2O in $CHCl_3$ for 2 h at 23 °C; (b) *atrop*-abyssomicin C (**8**); c) abyssomicin C (**5**).

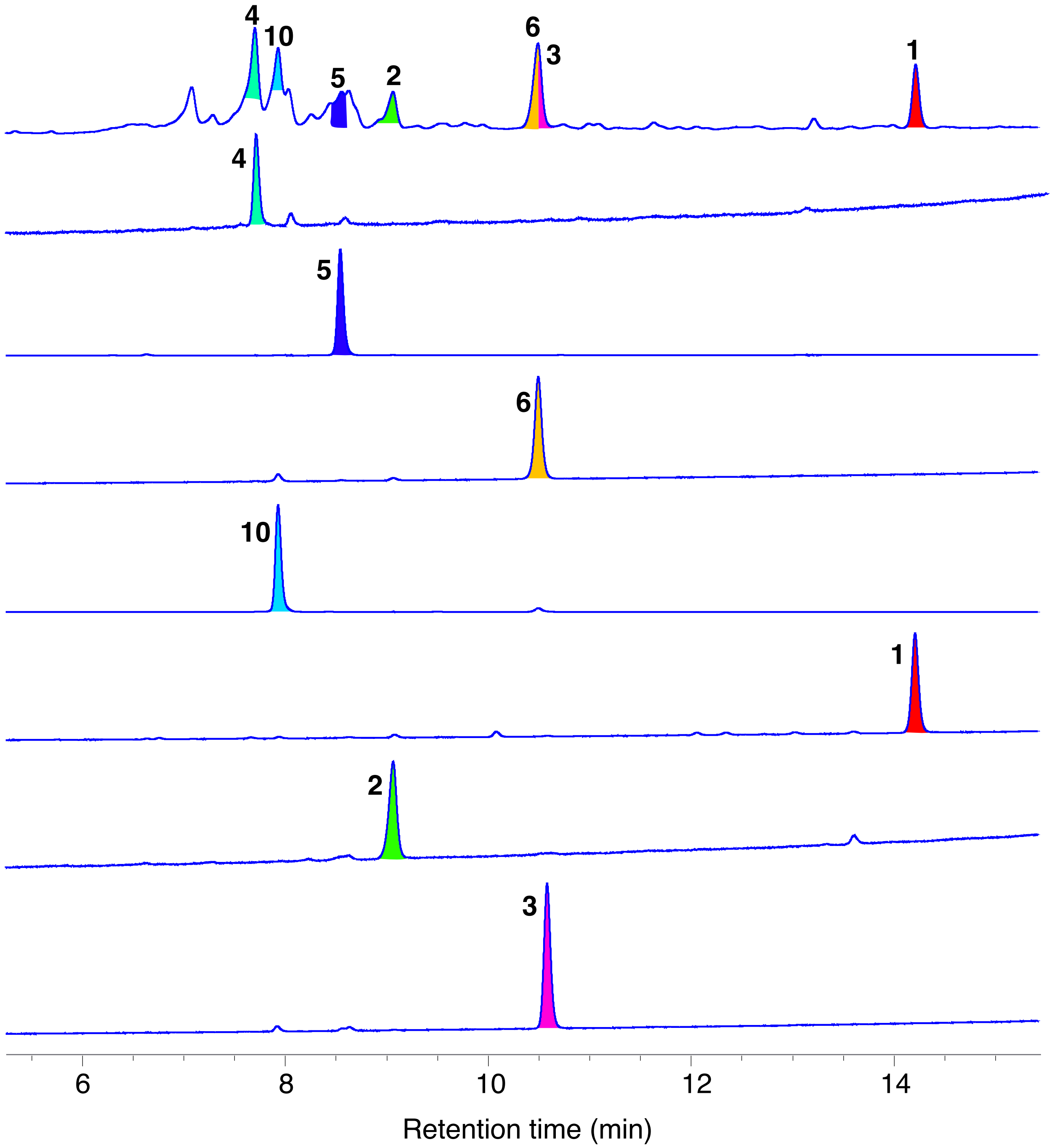

Figure S13. HPLC (Agilent Zorbax SB-C_8, 5 µm, 4.6 × 150 mm, 1.0 mL/min gradient elution from 10–100% MeCN/H_2O over 15 min with isocratic 0.05% formic acid modifier) analysis of crude extracts and purified abyssomicins **1–6**, **10**

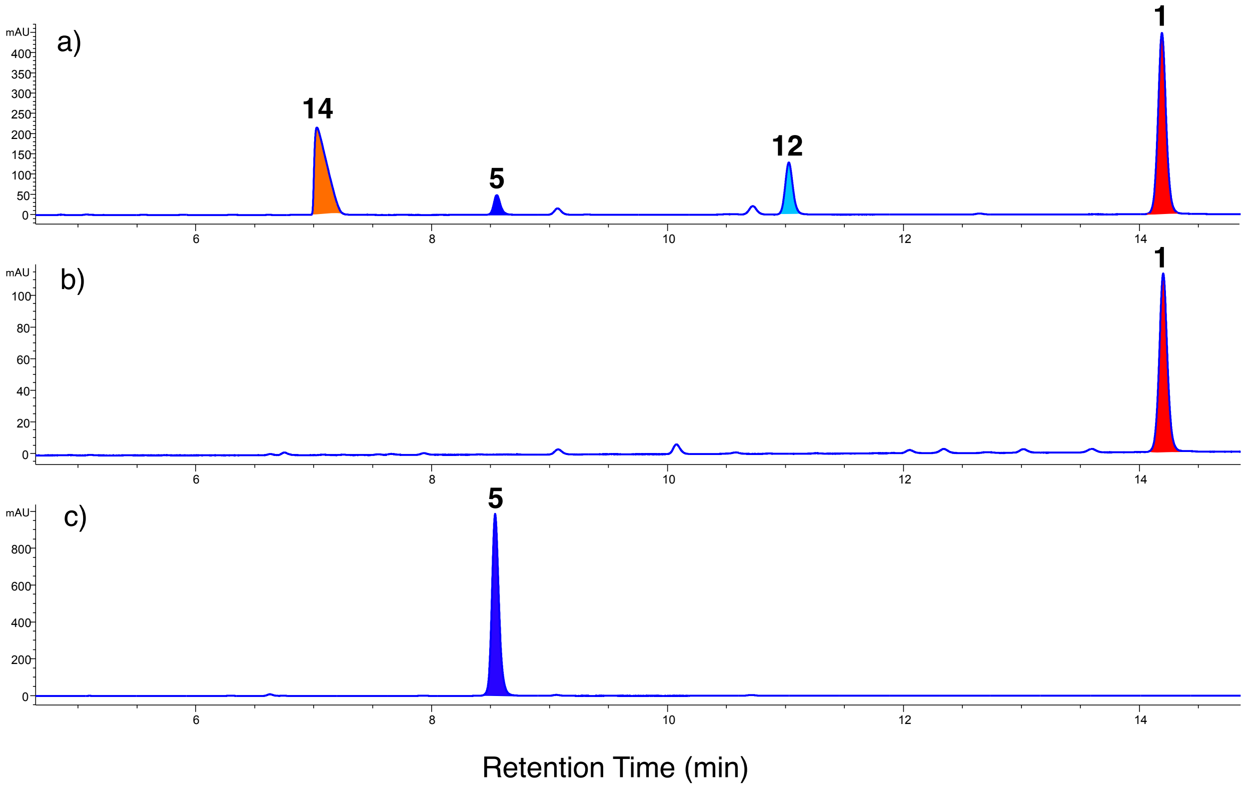

Figure S14. HPLC (Agilent Zorbax SB-C_8, 5 μm, 4.6 × 150 mm, 1.0 mL/min gradient elution from 10–100% MeCN/H_2O over 15 min with isocratic 0.05% formic acid modifier) analysis of (a) abyssomicin (**5**) treated with 0.1 M Na_2S in 50% H_2O/MeCN for 2 h at 23 °C; (b) abyssomicin J (**1**); c) abyssomicin C (**5**)

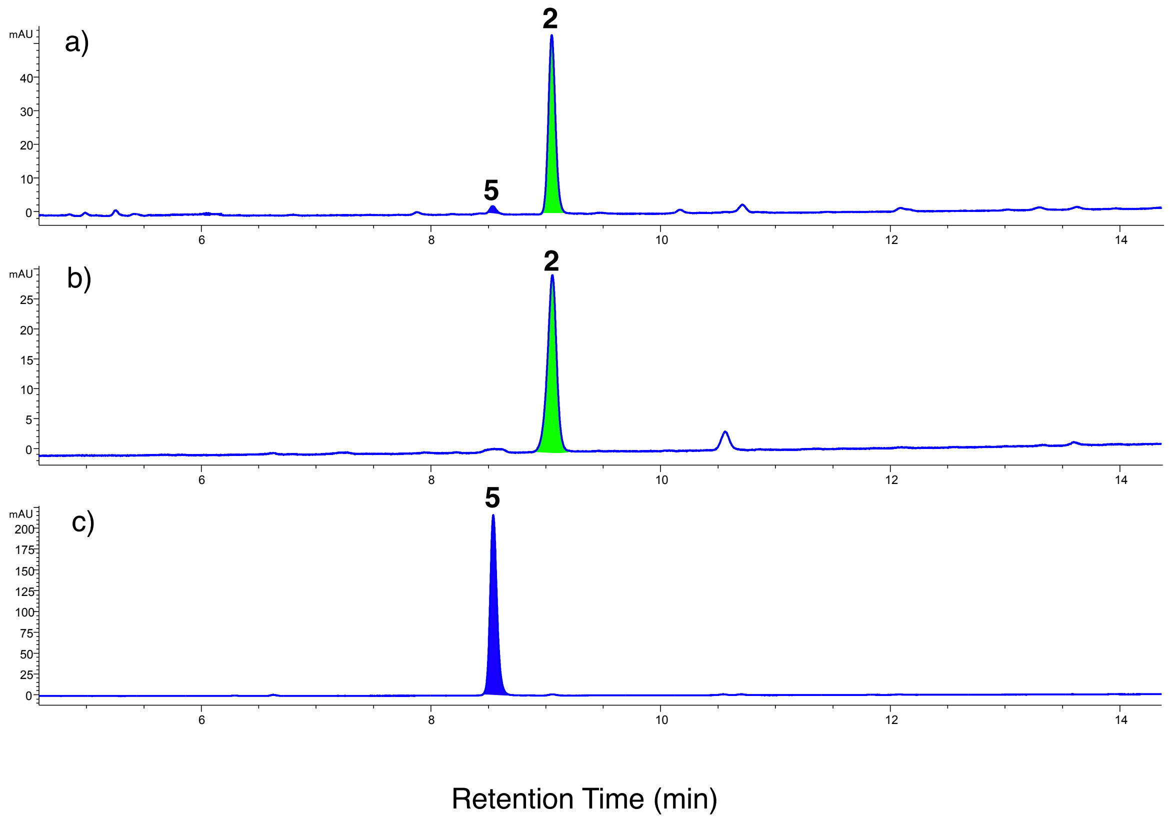

Figure S15. HPLC (Agilent Zorbax SB-C_8, 5 μm, 4.6 × 150 mm, 1.0 mL/min gradient elution from 10–100% MeCN/H_2O over 15 min with isocratic 0.05% formic acid modifier) analysis of (a) abyssomicin C (**5**) treated with 0.05 M NaOH in 50% H_2O/MeCN for 3 h at 23 °C; (b) abyssomicin K (**2**); c) abyssomicin C (**5**)

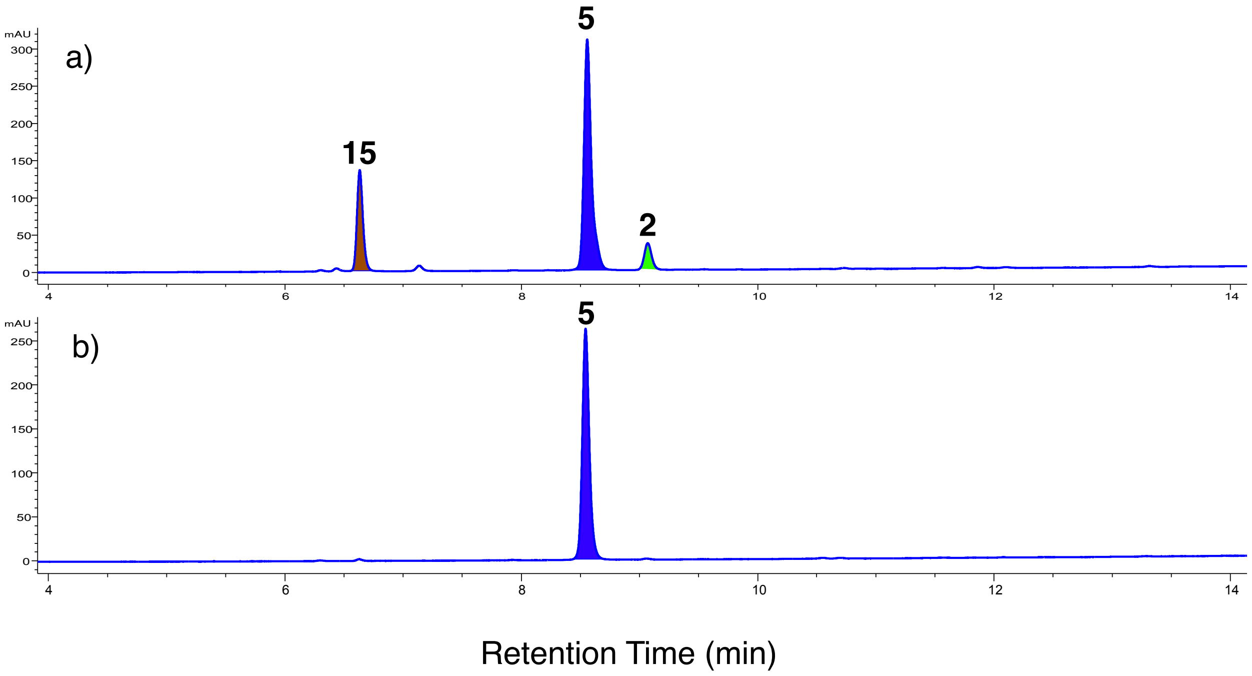

Figure S16. HPLC (Agilent Zorbax SB-C_8, 5 µm, 4.6 × 150 mm, 1.0 mL/min gradient elution from 10–100% $MeCN/H_2O$ over 15 min with isocratic 0.05% formic acid modifier) analysis of (a) abyssomicin C (**5**) treated with 0.5 M TFA in 50% $H_2O/MeCN$ for 12 h at 40 °C; (b) abyssomicin C (**5**)

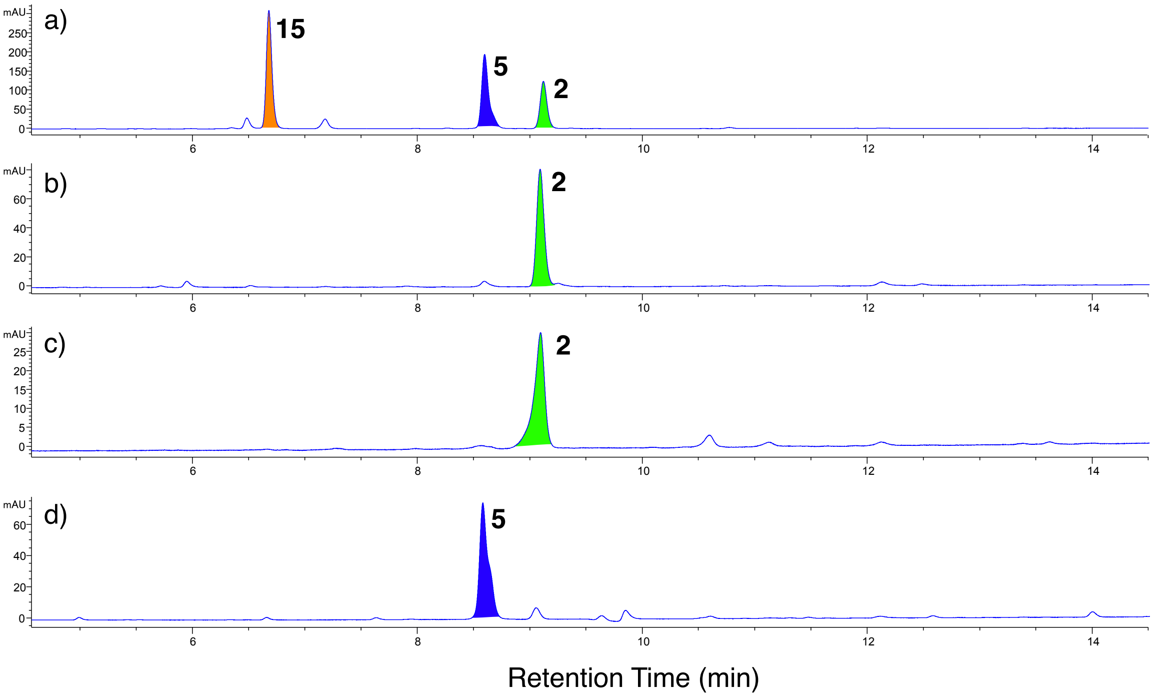

Figure S17. HPLC (Agilent Zorbax SB-C_8, 5 µm, 4.6 × 150 mm, 1.0 mL/min gradient elution from 10–100% $MeCN/H_2O$ over 15 min with isocratic 0.05% formic acid modifier) analysis of (a) abyssomicin C (**5**) treated with 0.5 M TFA in 50% $MeCN/H_2O$ for 48 h at 40 °C; (b) Mixture from (a) treated with 0.05 M NaOH for 3 h at 23 °C; c) abyssomicin K (**2**); d) abyssomicin C (**5**).

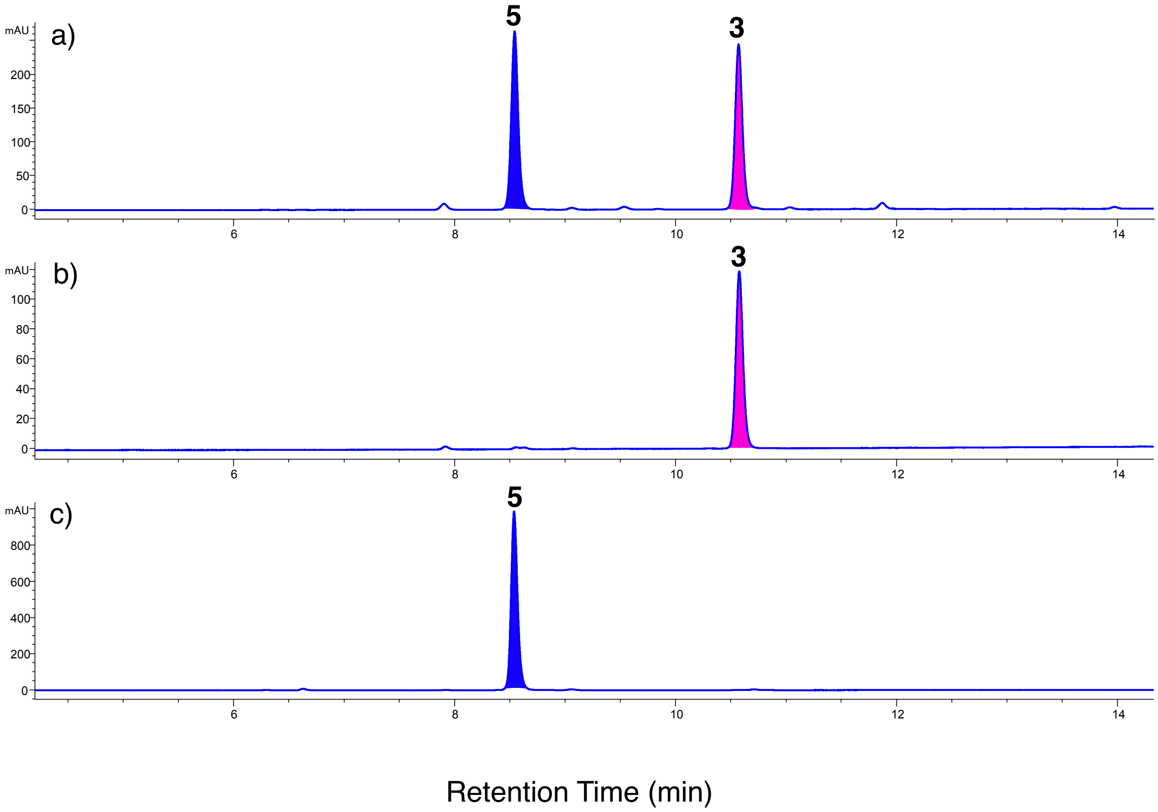

Figure S18. HPLC (Agilent Zorbax SB-C_8, 5 μm, 4.6 × 150 mm, 1.0 mL/min gradient elution from 10–100% MeCN/H_2O over 15 min with isocratic 0.05% formic acid modifier) analysis of (a) abyssomicin C (**5**) treated with 0.5 M TFA in MeOH for 72 h at 40 °C; (b) abyssomicin L (**3**); c) abyssomicin C (**5**)

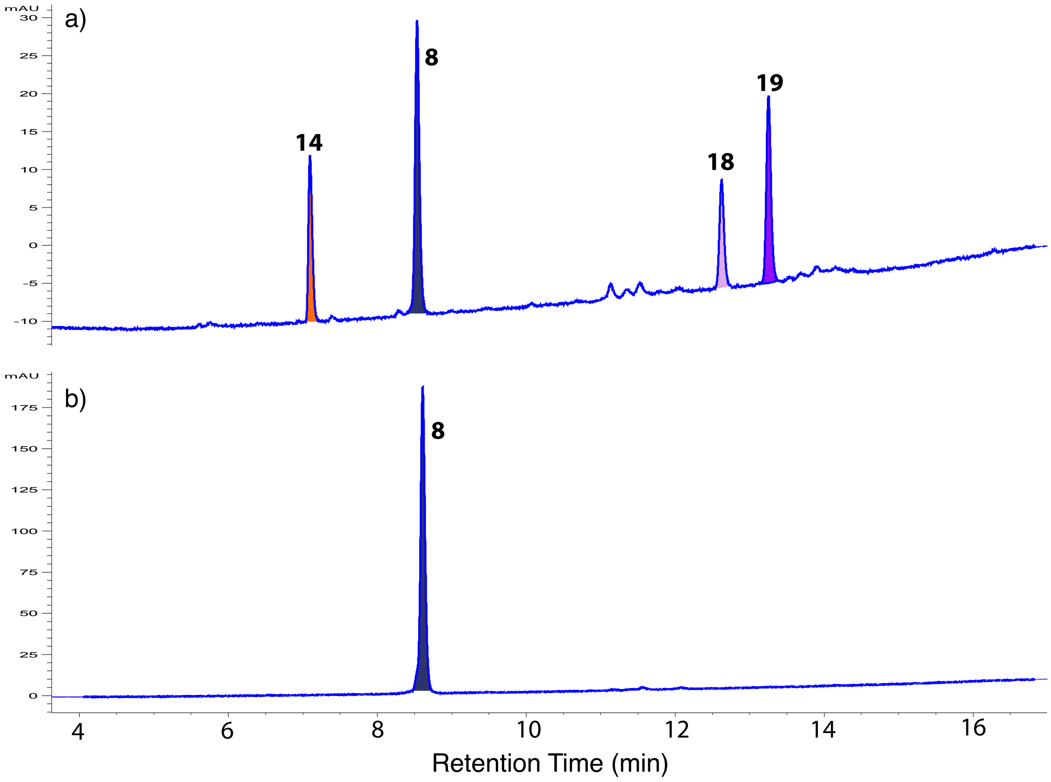

Figure S19. HPLC (Agilent Zorbax SB-C_8, 5 µm, 4.6 × 150 mm, 1.0 mL/min gradient elution from 10–100% MeCN/H_2O over 15 min with isocratic 0.05% formic acid modifier) analysis of (a) abyssomicin J (**1**) treated with 2 eq. Oxone in 50% H_2O/MeCN for 1 h at 23 °C; (b) *atrop*-abyssomicin C (**8**).

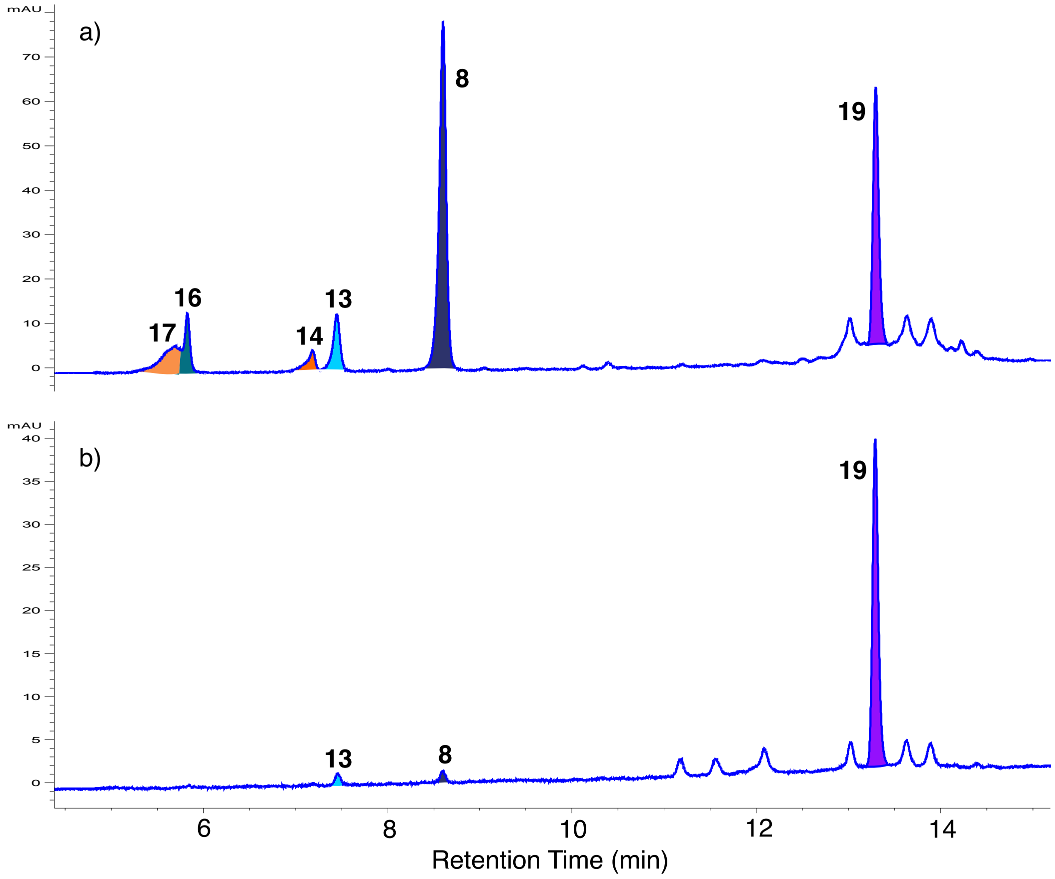

Figure S20. HPLC (Agilent Zorbax SB-C_8, 5 µm, 4.6 × 150 mm, 1.0 mL/min gradient elution from 10–100% MeCN/H_2O over 15 min with isocratic 0.05% formic acid modifier) analysis of (a) sulfone (**19**) after heating for 1 h at 40 °C in MeCN; (b) sulfone (**19**).

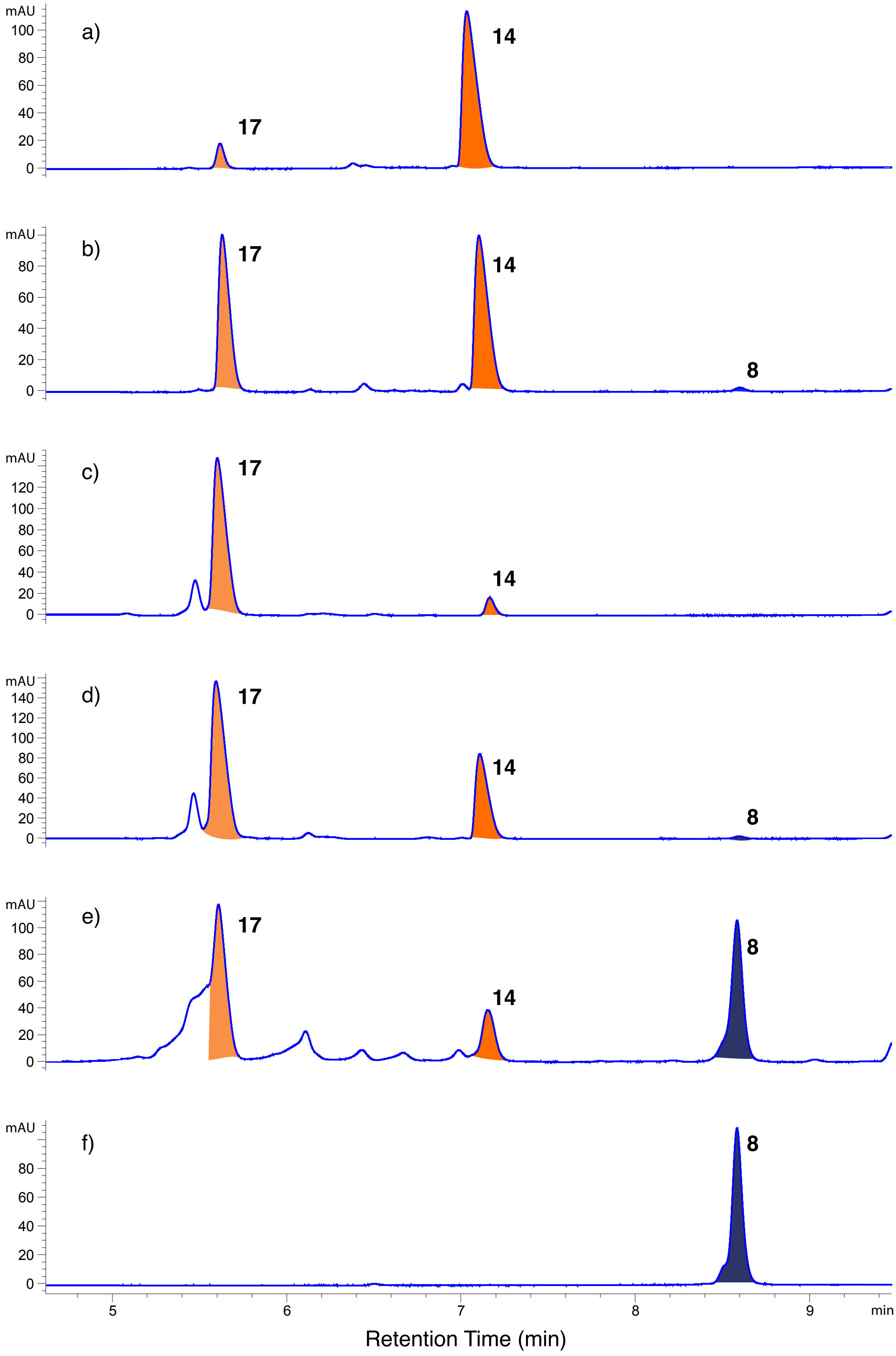

Figure S21. HPLC (Agilent Zorbax SB-C_8, 5 µm, 4.6 × 150 mm, 1.0 mL/min gradient elution from 10–100% MeCN/H_2O over 15 min with isocratic 0.05% formic acid modifier) analysis of (a) HPLC purified abyssomicin **14** after standing for 10 min at room temperature in MeCN; (b) abyssomicin **14** after heating for 1 h at 40 °C in MeCN; (c) HPLC purified abyssomicin **17** after standing for 10 min at room temperature in MeCN; (d) abyssomicin **17** after heating for 1 h at 40 °C in MeCN; (e) abyssomicin **14** after heating for 12 h at 70 °C in MeCN; (f) *atrop*-abyssomicin C (**8**).

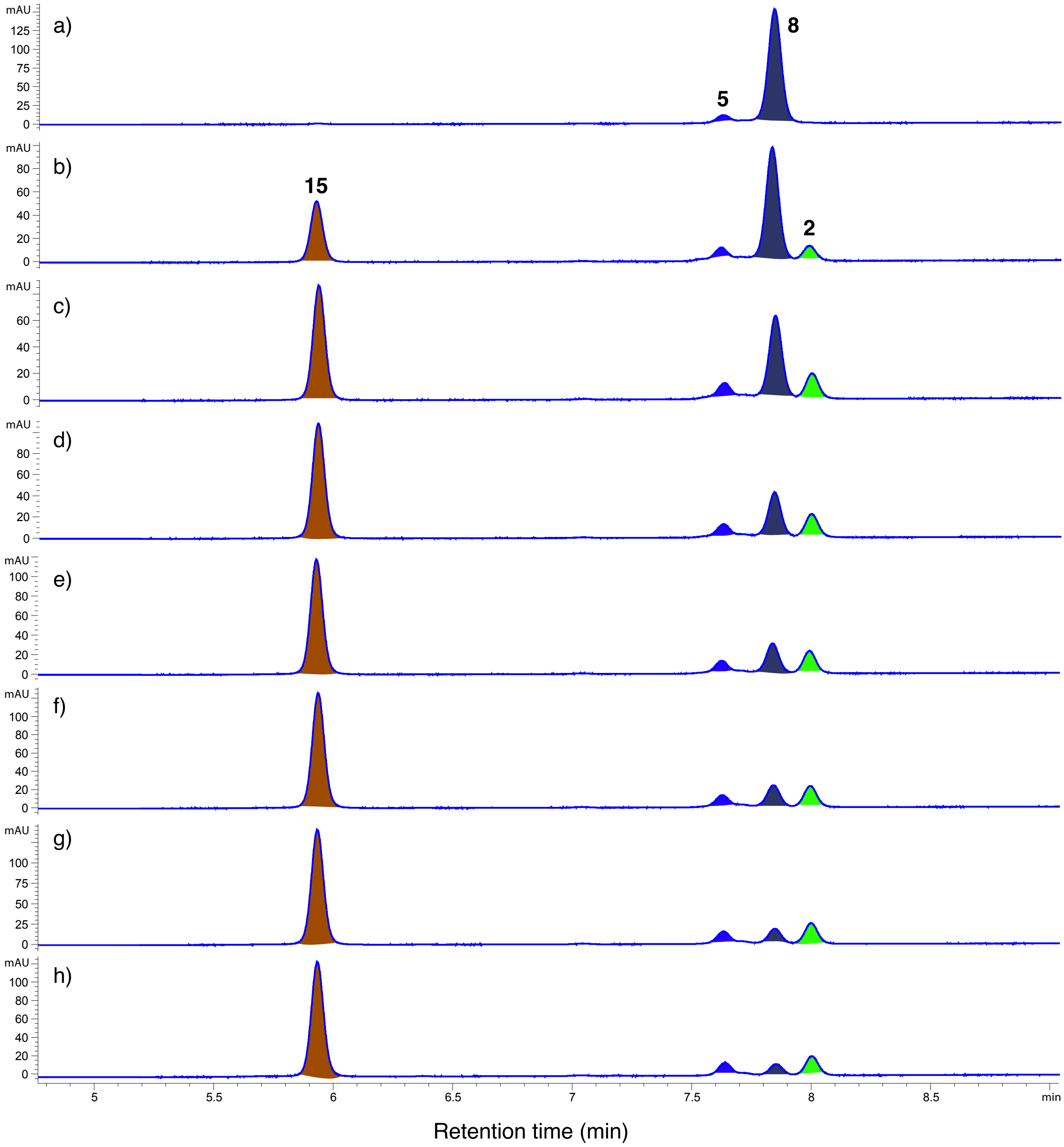

Figure S22. HPLC (Agilent Zorbax SB-CN, 5 μm, 4.6 × 150 mm, 1.0 mL/min gradient elution from 10–100% MeCN/H_2O over 15 min with isocratic 0.05% formic acid modifier) analysis of *atrop*-abyssomicin C (**8**) after standing in 10% MeCN/H_2O + 0.1 M TFA at 40 °C for (a) 5 min; (b) 1.5 h; (c) 3 h; (d) 4.5 h; (e) 6 h; (f) 7.5 h; (g) 11 h; (h) 18 h.

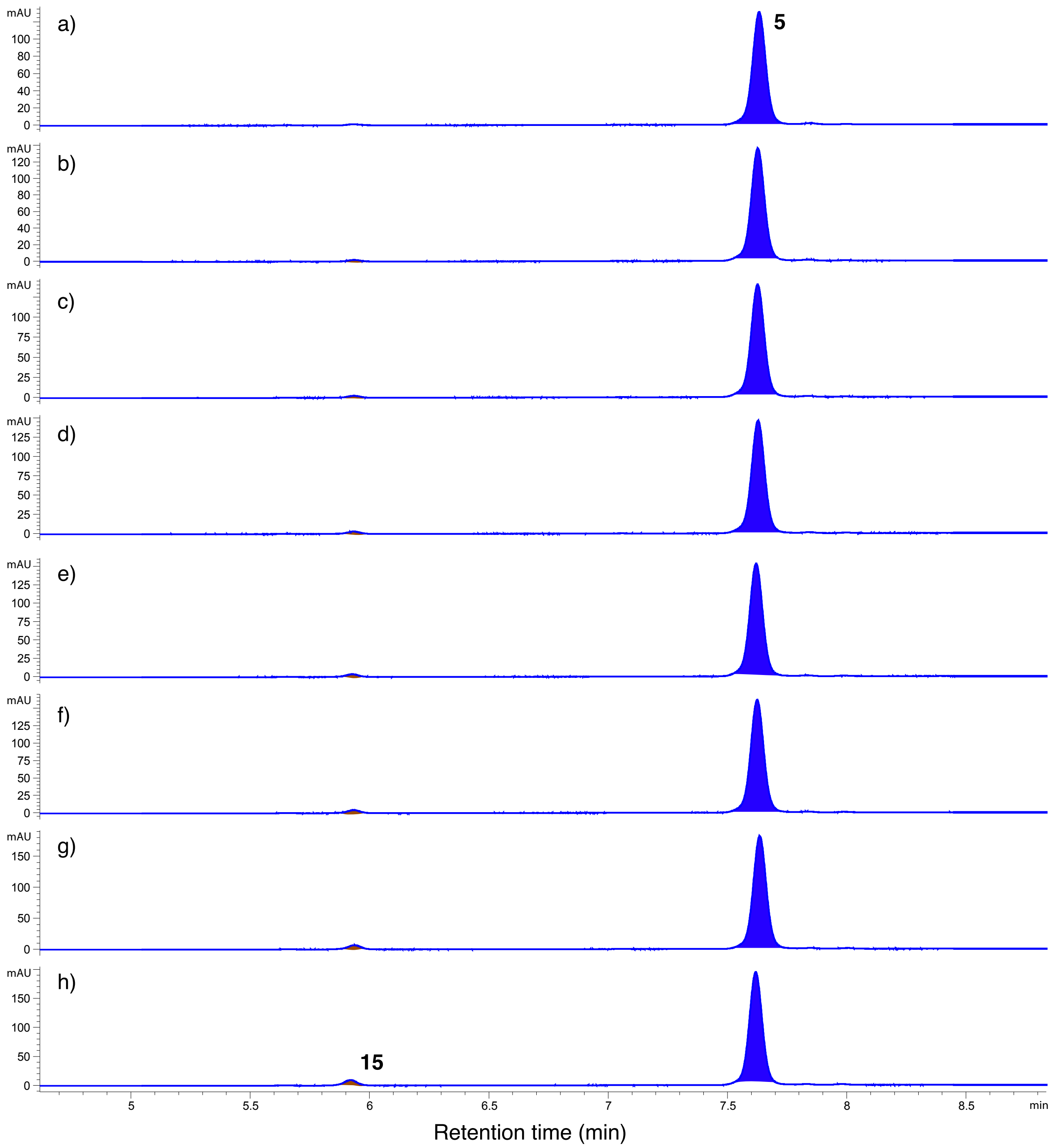

Figure S23. HPLC (Agilent Zorbax SB-CN, 5 μm, 4.6 × 150 mm, 1.0 mL/min gradient elution from 10–100% MeCN/H_2O over 15 min with isocratic 0.05% formic acid modifier) analysis of abyssomicin C (**5**) after standing in 10% MeCN/H_2O + 0.1 M TFA at 40 °C for (a) 5 min; (b) 1.5 h; (c) 3 h; (d) 4.5 h; (e) 6 h; (f) 7.5 h; (g) 11 h; (h) 18 h.

Table S11 Antimicrobial activities of abyssomicins **1**–**6** and **10**

Organism (Strain)	Minimum inhibitory concentration (μg/mL) J (**1**)	K (**2**)	L (**3**)	B (**4**)	C (**5**)	D (**6**)	H (**10**)	Control
Bacille Calmette-Guérin (Pasteur 1173P2)	3.125	25	100	>100	6.25	>100	25	0.05[a]
Mycobacterium tuberculosis H37Rv (ATCC 27294)	50	>100	>100	>50	25	>50	>50	0.025[a]
Staphylococcus aureus (ATCC 6538)	12.5	>100	>100	>100	50	>100	>100	1 [b]
Bacillus subtilis (ATCC 6633)	50	>100	>100	>100	100	>100	>100	0.5[b]
Pseudomonas aeruginosa (PAO1)	>100	>100	>100	>100	>100	>100	>100	1[c]
Candida albicans (SC 5314)	>100	>100	>100	>100	>100	>100	>100	0.016[d]

[a] isoniazid [b] vancomycin [c] ciprofloxacin [d] ketoconazole

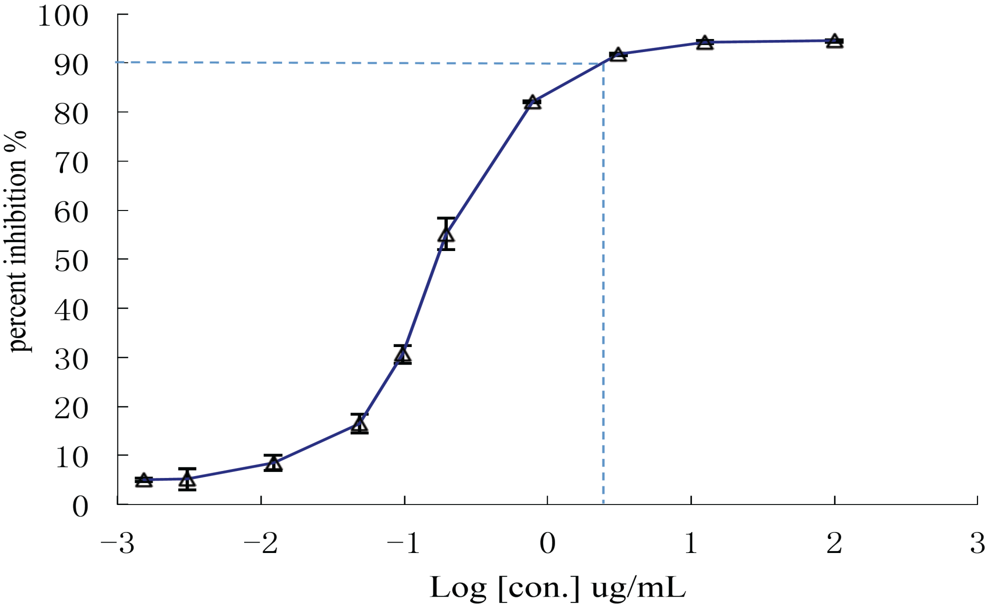

Figure S24. Dose-dependent inhibition of BCG by abyssomicin J (**1**)

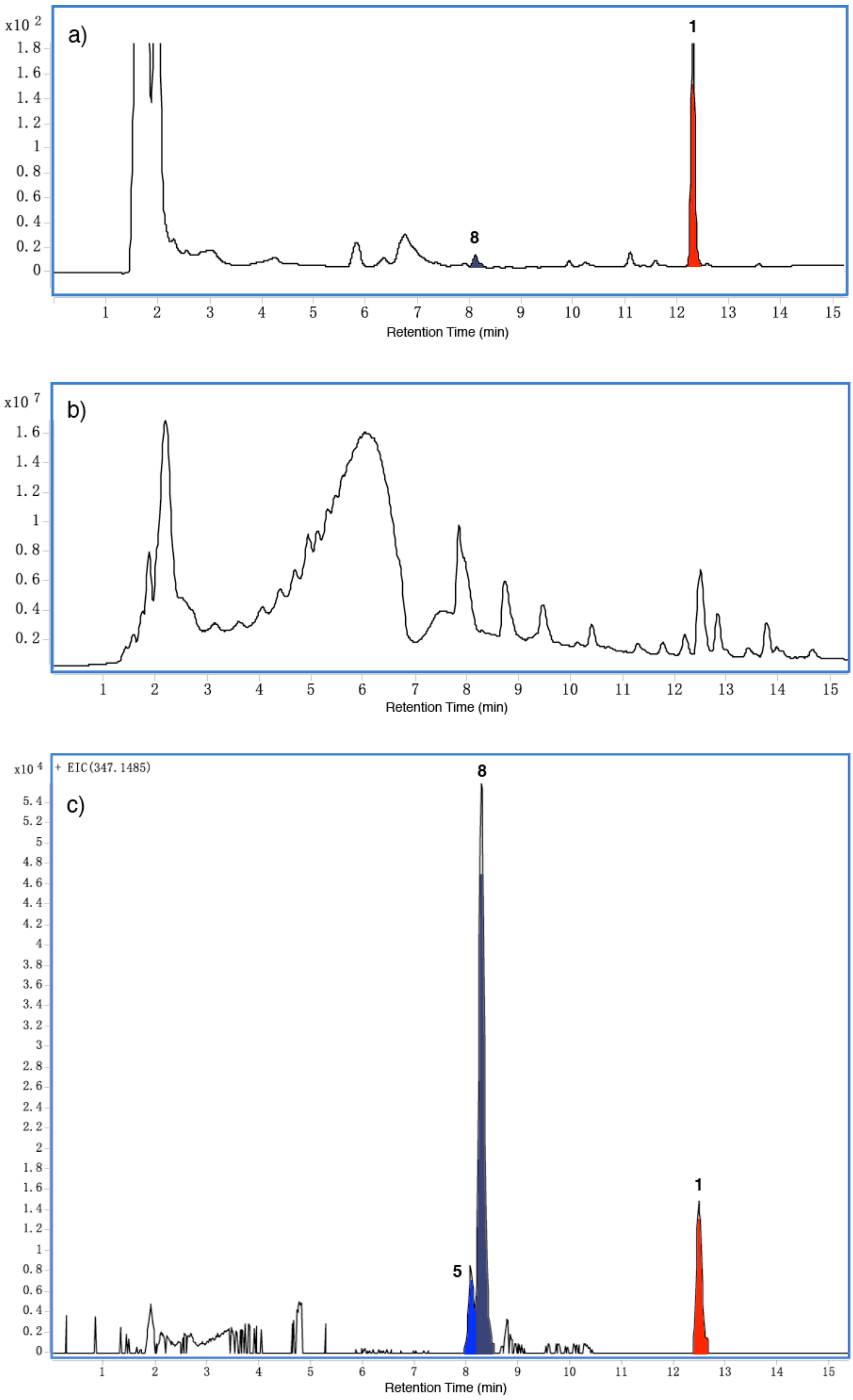

Figure S25. HPLC-DAD-HRESI(+)MS (Agilent Zorbax SB-CN, 5 µm, 4.6 × 150 mm, 1.0 mL/min gradient elution from 10–100% MeCN/H_2O over 15 min with isocratic 0.05% formic acid modifier) analysis of abyssomicin J (**1**) exposed to BCG for 1 h at 37 °C. (a) HPLC trace at 254 nm; (b) total positive ion chromatogram; (c) extracted positive ion chromatogram for 347.1485 (corresponding to $[M+H]^+$ for **8**).
